# Association Between Socioeconomic Status and Atrial Fibrillation: A Two‐Sample Mendelian Randomization Study

**DOI:** 10.1111/anec.70227

**Published:** 2026-08-03

**Authors:** Shangcai Xie, Xia Zhao, Menghui Mao

**Affiliations:** ^1^ Department of Cardiology The Second Affiliated Hospital of Jiaxing University Jiaxing Zhejiang China

**Keywords:** atrial fibrillation, educational attainment, household income, mendelian randomization, socioeconomic status, Townsend deprivation index

## Abstract

**Background:**

Lower socioeconomic status (SES), reflected by greater socioeconomic deprivation, lower household income, and lower educational attainment (EA), has been associated with atrial fibrillation (AF). This study aimed to examine the causal influence of SES on AF risk.

**Methods:**

Single‐nucleotide polymorphisms (SNPs) associated with Townsend deprivation index (TDI) or household income from the UK Biobank (*N* = 462,464 and *N* = 397,751, respectively) and EA from the Social Science Genetic Association Consortium (*N* = 3,037,499) were selected as instrumental variables. AF‐associated SNPs were extracted from FinnGen data freeze 8 (*N* = 208,594) and a meta‐analysis (*N* = 1,030,836) by Nielsen et al. Two‐sample Mendelian randomization (MR) was used to estimate causal relationships, with validity assessed via sensitivity analyses.

**Results:**

TDI showed no association with AF risk in either FinnGen (odds ratio (OR) = 0.972, 95% confidence interval (CI): 0.607–1.556, *p* = 9.00E‐01) or Nielsen et al. (OR = 0.805, 95% CI: 0.470–1.381, *p* = 4.30E‐01). Similarly, genetically proxied household income was not associated with AF risk (OR = 1.233, 95% CI: 0.808–1.881, *p* = 3.30E‐01; OR = 0.941, 95% CI: 0.755–1.173, *p* = 5.90E‐01). However, higher genetically proxied EA was associated with lower AF risk in FinnGen (OR = 0.745, 95% CI: 0.653–0.849, *p* = 1.00E‐05) and Nielsen et al. (OR = 0.849, 95% CI: 0.784–0.920, *p* = 5.90E‐05).

**Conclusion:**

Our MR study found that higher genetically proxied EA was associated with lower AF risk, whereas no evidence of association was found for genetically proxied TDI or household income.

## Graphical abstract

Mendelian randomization revealed that lower educational attainment increases atrial fibrillation risk, whereas Townsend deprivation index and household income showed no causal association. These findings highlight education as a key socioeconomic factor influencing atrial fibrillation susceptibility.

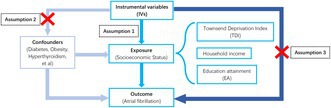

## 1 Introduction

Atrial fibrillation (AF) refers to the most prevalent cardiac arrhythmia in clinical practice. Patients show a high risk of death, heart failure, hospitalization, and thromboembolic events (Benjamin et al. 1998; Patel et al. 2014). AF affects more than 33 million individuals worldwide (Chung et al. 2020), with research indicating its increasing frequency and incidence globally (Lip et al. 2012). In Europe and North America, AF affects approximately 2% and 3% of the population as of 2014 (Zoni‐Berisso et al. 2014), respectively. The number of cases diagnosed with AF has risen due to improved detection of silent AF, aging, and predisposing factors (Hindricks et al. 2021).

Socioeconomic status (SES) is a multidimensional economic and sociological construct reflecting an individual's or family's access to economic resources and relative social position in comparison with that of others (Oakes and Rossi 2003). Common indicators of SES include the Townsend Deprivation Index (TDI) (Townsend et al. 2023), educational attainment, and household income (Turčínková and Stávková 2011). Previous observational studies have reported that greater socioeconomic deprivation, lower household income, and lower educational attainment (EA) are associated with an increased risk of AF (Lunde et al. 2020; McCauley and Darbar 2018; Ramkumar et al. 2019). However, observational studies are susceptible to residual confounding and other biases, while randomized controlled trials that directly assign socioeconomic circumstances are generally impractical and may be ethically problematic. Therefore, whether these associations reflect causal effects of SES on AF remains uncertain.

Mendelian randomization (MR) refers to the genetics‐based method of assessing the causal influence of an exposure on an outcome when instrumental variables (IVs) are involved (Davies et al. 2018). The design decreases reverse causality and confounding compared with typical epidemiological methodologies.

In this study, we performed two‐sample MR to determine the potential causal relationship between SES and AF using different large‐scale genome‐wide association study (GWAS) data.

## 2 Methods

### 2.1 Study Design

**FIGURE 1:**
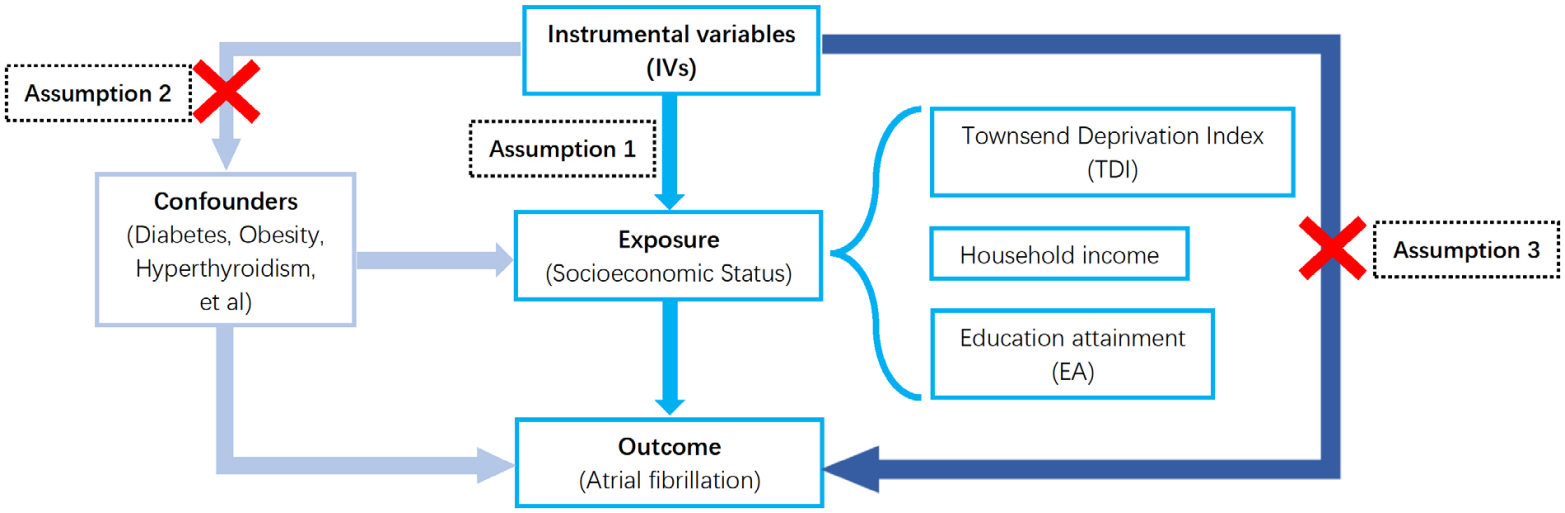
Two‐sample MR directed acyclic graph of the three key assumptions; (1) the IVs are associated with the SES; (2) the IVs are not associated with confounders; and (3) the IVs influence AF only through the SES.

This two‐sample MR was carried out in strict compliance with the most recent recommendations for MR analysis, *Strengthening the Reporting of Observational Studies in Epidemiology Using Mendelian Randomization: The STROBE‐MR Statement* (Skrivankova et al. 2021) cleotide polymorphisms (SNPs) were obtained, and based on three key assumptions: (1) “Relevance”: the genetic variants being used as an instrument for exposure (SES) are associated with SES (*p* < 5.00E‐08). (2) “Independence”: genetic variants and outcomes have no confounders (AF). (3) “Exclusion restriction”: no independent pathway exists between genetic variants and AF other than through the SES (Davies et al. 2018; Lawlor et al. 2008) (Figure 1).

### 2.2 IVs For Exposures

GWAS summary statistics for TDI (*N* = 462,464) and household income (*N* = 397,751) were obtained from the UK Biobank (UKB) through the MRC IEU OpenGWAS Project (GWAS IDs: ukb‐b‐10,011 and ukb‐b‐7408, respectively) (Lyon et al. 2021; Ben et al. 2020). The GWAS analyses were adjusted for sex and genotyping array when BOLT‐LMM v2.3 was used and additionally adjusted for the first 10 principal components when PLINK v2.00 was used. The SNP–exposure association estimates were expressed in SD units; therefore, the MR estimates were interpreted per one‐standard‐deviation (SD) increase in the corresponding exposure.

TDI (UKB Data‐Field 189) is a continuous, area‐level measure of material deprivation assigned according to the census output area corresponding to each participant's residential postcode. It is derived from census‐based indicators including unemployment, non‐car ownership, non‐home ownership, and household overcrowding. A higher TDI value indicates greater socioeconomic deprivation, whereas a lower value indicates relative socioeconomic advantage. In UKB, the SD of TDI was approximately 3.09 units; therefore, the corresponding MR odds ratios represent the change in AF odds per one‐SD increase, equivalent to an approximately 3.09‐unit increase in the original TDI score. Household income (UKB Data‐Field 738) was assessed using the touchscreen question, “What is the average total income before tax received by your household?” Responses were recorded as an ordered categorical variable: 1, less than £18,000; 2, £18,000–£30,999; 3, £31,000–£51,999; 4, £52,000–£100,000; and 5, greater than £100,000. “Do not know” and “prefer not to answer” responses were treated as missing in the source GWAS. Higher values therefore indicate higher household income. The corresponding MR odds ratios were interpreted per one‐SD increase in the standardized household‐income phenotype. Further details are available in the UK Biobank showcase.

EA‐associated single‐nucleotide polymorphisms (SNPs) were obtained from the Social Science Genetic Association Consortium (SSGAC). The source GWAS was a recent meta‐analysis (Okbay et al. 2022) that combined association results from 69 previously analyzed cohorts excluding UKB and 23andMe (*N* = 324,162) (Lee et al. 2018) with updated results from UKB (*N* = 441,121) and 23andMe (*N* = 2,272,216). The final GWAS included 3,037,499 individuals of European genetic ancestry. The GWAS analyses included adjustment for sex, year of birth, the interaction between sex and year of birth, genetic principal components, and additional cohort‐specific covariates, as appropriate (Table 1). In the UKB component, participants answered the following questions related to their EA: (1) “Which of the following qualifications do you have? You can select more than one” (qualifications included a college or university degree, Advanced/Advanced Subsidiary Levels or equivalent, National Vocational Qualification, Higher National Diploma, Higher National Certificate, or equivalent qualifications); and (2) for individuals without a college degree, “At which age did you complete your continuous full‐time education?” (Okbay et al. 2016) Educational qualifications reported across the participating cohorts were harmonized and converted into corresponding years of schooling using established procedures. EA was coded from 7 to a maximum of 20 years, with higher values indicating greater educational attainment. The EA phenotype was standardized to unit variance in the source GWAS; therefore, the corresponding MR odds ratios represent the change in AF odds per one‐SD increase in genetically proxied EA rather than per one‐year increase. All IVs were related to the exposure at a genome‐wide significance level (*p*‐value < 5.00E‐08) with linkage disequilibrium (LD) R^2^ < 0.001 at a 10,000‐kb window, which confirmed the independence of the selected genetic variations.

**TABLE 1:** GWAS summary data sources of exposures and outcomes.

Phenotype (Trait)	Data source (Consortium)	Sample size (Case/Contro)	Ancestry	Covariates	Link
Exposures					
TDI	UK Biobank (MRC‐IEU)	462,464	European	For BOLT‐LMM(v2.3): sex and genotyping array; for PLINK(v2.00): sex, genotyping array and the first 10 principal components(PCs).	https://gwas.mrcieu.ac.uk/datasets/ukb‐b‐10011/
HI	UK Biobank (MRC‐IEU)	397,751	European		https://gwas.mrcieu.ac.uk/datasets/ukb‐b‐7408/
EA	SSGAC	3,037,499	European	Common:sex, age, age^2^, age^3^, and their interactions with sex; additional covariates: for UKB: the top 40 PCs, and batch dummies; for GS and STR: the top 20 PCs.	https://thessgac.com/papers/
Outcome					
AF	Nielsen et al.	1,030,836 (60,620/970,216)	European	For HUNT: birth year, sex, genotype batch, and PCs 1–4 as implemented in SAIGE; for deCODE: allele counts from direct genotyping or expected genotype counts from imputation; for MGI: age, sex, and PCs 1–4; for DiscovEHR: sex, age, age2, and the first 4 PCs of ancestry; for UKB: birth year, sex, genotype batch, and PCs 1–4 as implemented in SAIGE.	https://gwas.mrcieu.ac.uk/datasets/ebi‐a‐GCST006414/ http://csg.sph.umich.edu/willer/public/afib2018/
AF	FinnGen	208,594 (40,594/168,000)	European	sex, age, 10 PCs, genotyping batch.	https://storage.googleapis.com/finngen‐public‐data‐r8/summary_stats/finngen_R8_I9_AF.gz

We further calculated the F‐statistics for each SNP to evaluate weak IVs:

(1) $$F=\frac{\left(N-k-1\right)\times R^{2}}{k\times \left(1-R^{2}\right)}$$

where *F* is *F*‐statistics, *N* refers to the sample size of the GWAS for the association between SNPs and the corresponding exposure, k indicates the numbers of IV, and *R* ^2^ is the proportion of variance in the corresponding exposure accounted for by each instrument (Papadimitriou et al. 2020). Where $R^{2}$ can be calculated via the following methods (R_1_ or R_2_):

(2) $$R_{1}^{2}=\frac{2\times \mathrm{EAF}\times \left(1-\mathrm{EAF}\right)\times \beta^{2}}{2\times \mathrm{EAF}\times \left(1-\mathrm{EA}F\right)\times \beta^{2}+2\times \mathrm{EAF}\times \left(1-\mathrm{EAF}\right)\times N\times \mathrm{SE}{\left(\beta \right)}^{2}}$$

(3) $$R_{2}^{2}=2\times \left(1-\mathrm{MAF}\right)\times \mathrm{MAF}\times \frac{\beta }{\mathrm{SD}}$$

(4) $$\mathrm{SD}=\mathrm{SE}\times \sqrt{N}$$

where EAF means the effect allele frequency, beta represents the expected effect of genetics on the corresponding exposure, β denotes the effect size of a SNP on exposure, SE(*β*)^2^ corresponds to the standard error of the genetic effect, MAF indicates the minor allele frequency, and SD means standard deviation (Shim et al. 2015).

Genetic variation with *F* < 10 indicates a weak IV and may bias the results to some extent. To further emphasize Assumption 2, we performed a related second phenotype screening (defaulted *R* ^2^ > 0.8, *p* < 1.00E‐05 and genome build by GRCh37) similar to those conducted on diabetes (Benjamin et al. 1994), obesity (body mass index) (Wang et al. 2004), hypertension (systolic and diastolic blood pressures) (Krahn et al. 1995; Kannel et al. 1982), coronary artery disease (Liberthson et al. 1976), hyperthyroidism (Woeber 1992), smoking (Groh et al. 2019; Staerk et al. 2017), alcohols (Djousse et al. 2004), and so on, which can serve as confounders, utilizing PhenoScanner version 2. Similarly, we manually deleted the SNPs that were strongly associated with the outcome (*p* < 5.00E‐08) to emphasize Assumption 3. Afterward, we excluded palindromic SNPs with intermediate allele frequencies.

**FIGURE 2:**
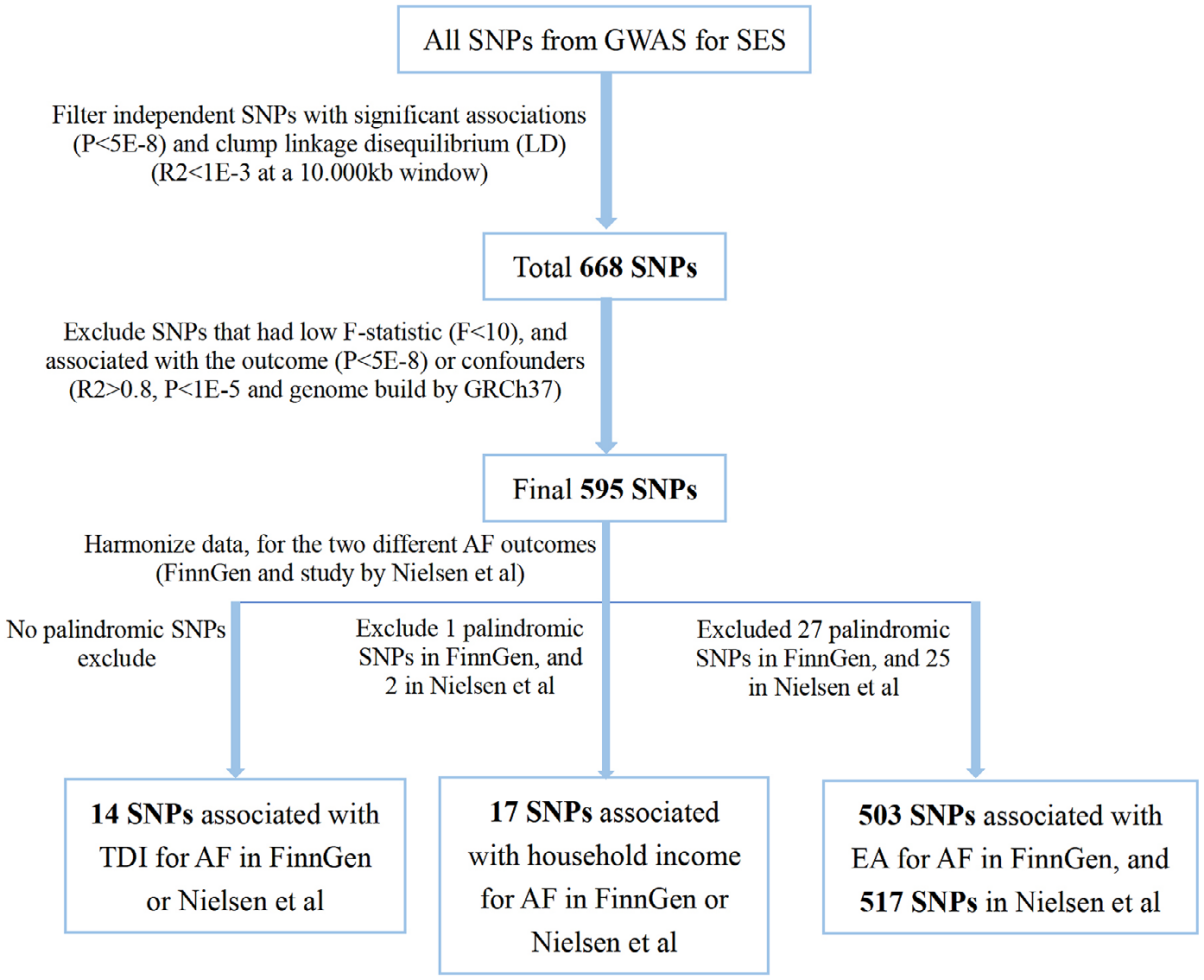
The schematic diagram of instrumental variables selection. SNPs, single‐nucleotide polymorphisms; AF, atrial fibrillation.

Finally, 14 TDI‐associated and 27 household‐income‐associated SNPs were retained as IVs for both the FinnGen and Nielsen et al. analyses. For EA, 503 and 517 SNPs were available after harmonization with FinnGen and Nielsen et al., respectively. All retained variants were genome‐wide significant, mutually independent after LD clumping, and had F‐statistics greater than 10. The complete rsID‐level information and corresponding SNP‐association estimates are provided in Tables S1–S6, while SNPs identified as potential outliers by MR‐PRESSO are reported in Table 3. Figure 2 depicts the entire instrument‐selection process.

### 2.3 Outcome Data Sources

To address potential sample overlap and assess the reproducibility of the findings, the associations of the selected instrumental variables with AF were extracted from two large summary‐level GWAS datasets. First, the meta‐analysis by Nielsen et al. (GWAS ID: ebi‐a‐GCST006414), the largest AF GWAS available at the time of our analysis, included 60,620 AF cases and 970,216 controls (Nielsen et al. 2018). This GWAS included 395,739 UKB participants. Therefore, the potential UKB‐derived overlap with the UKB‐based TDI and household‐income exposure datasets could involve up to 395,739 participants, corresponding to 38.39% of the total Nielsen et al. sample. Second, FinnGen Data Freeze 8 included 40,594 AF cases and 168,000 controls and was adjusted for sex, age, the first 10 principal components, and genotyping batch (Mitja et al. 2022). FinnGen did not include UKB participants and therefore had no sample overlap with the UKB‐based TDI and household‐income exposure datasets.

Regarding EA, the SSGAC GWAS comprised participants from UKB (*N* = 441,121), 23andMe (*N* = 2,272,216), and 69 additional cohorts (*N* = 324,162) (Okbay et al. 2022; Lee et al. 2018). Because the Nielsen et al. AF GWAS included 395,739 UKB participants, the potential UKB‐derived overlap between the SSGAC EA GWAS and the Nielsen et al. dataset could involve up to 395,739 participants, corresponding to approximately 13.03% of the SSGAC EA sample and 38.39% of the Nielsen et al. sample. FinnGen had no known cohort‐level overlap with the UKB or 23andMe components of the SSGAC GWAS. However, because cohort‐level participant information for the remaining 69 SSGAC cohorts was unavailable from the publicly accessible summary‐level data, additional overlap with the Nielsen et al. dataset and possible overlap with FinnGen could not be precisely quantified or completely excluded. Table 1 summarizes the characteristics and sources of the exposure and outcome GWAS datasets, whereas detailed information on sample overlap and the related assessments is provided in Table 3.

### 2.4 Statistical Analysis

The multiplicative random‐effects inverse‐variance weighted (IVW‐MRE) method was the primary method used in our two‐sample MR to estimate the potential causal relationship between exposures and outcomes; it combines Wald ratios (or ratio estimates) in an IVW meta‐analysis with adjustment for heterogeneity (Bowden et al. 2017). The IVW‐FE method was additionally reported as a complementary analysis (Burgess et al. 2013; Bowden et al. 2019). Furthermore, we carried out additional MR analyses to complement the IVW and test previous assumptions: (1) MR–Egger regression was used to estimate the causal effect adjusted for any directional pleiotropy (Bowden, Del Greco, et al. 2016; Schmidt and Dudbridge 2018); (2) weighted median was defined as the 50th percentile of either an unweighted or IVW empirical density function of the Wald ratio (or ratio estimates) (Bowden, Davey Smith, et al. 2016); (3) MR using a robust adjusted profile score (MR‐RAPS) enabled the method to maximize the profile likelihood of the Wald ratio (or ratio estimates) and accounted for weak instrument bias, pleiotropy, and extreme outliers (Zhao et al. 2018). All SNPs may be invalid due to pleiotropy when pleiotropy is balanced utilizing any of the aforementioned evaluation methods. In addition, we used the MR Pleiotropy RESidual Sum and Outlier (MR‐PRESSO) method, which is also an extension of IVW, to automatically identify outliers (Verbanck et al. 2018).

### 2.5 Sensitivity and Additional Analysis

In addition, we conducted thorough sensitivity and additional analyses: (1) Cochran's Q was the heterogeneity statistic for the IVW model; a Q significantly larger than its degrees of freedom indicates heterogeneity and incorrect instruments (Bowden et al. 2019; Greco et al. 2015); (2) MR–Egger analysis's intercept values were used to test for horizontal pleiotropy; (3) the leave‐one‐out method was applied to assess the robustness of findings and determine whether the causal relationship is altered by outlier SNPs alone; (4) MR‐Scatter plot: visualization of exposure results on outcomes estimated under different parameter estimation methods; (5) funnel plot: visualization of estimated heterogeneity; (6) forest plot: how each SNP's connection between exposure and outcome varies.

The statistical power calculations for this MR were performed on mRnd. The complete results are shown in Tables 2 and 3.

**TABLE 2:** Heterogeneity and pleiotropy test between the exposures and outcomes.

Exposures	Outcomes		Heterogeneity test		Pleiotropy test	
			IVW		MR‐Egger	
			Cochran's Q	*p*	Intercept	*p*
TDI	AF	Nielsen et al.	42.75	4.94E‐05	0.018	3.92E‐01
		FinnGen	11.69	5.53E‐01	−0.035	7.38E‐02
HI		Nielsen et al.	54.91	7.78E‐04	−0.011	2.26E‐01
		FinnGen	79.33	2.66E‐07	−0.025	2.10E‐01
EA		Nielsen et al.	827.46	7.76E‐07	−0.001	3.24E‐01
		FinnGen	868.87	4.66E‐22	−0.003	1.77E‐01

**TABLE 3:** MR‐PRESSO, sample overlap, and statistical power analysis.

Exposures	Outcomes		MR‐PRESSO			Known/potential sample overlap, *n* (%)	R2 (total)	F (total)	Power				
			Global test	Outliers	Distortion test				OR				
			*p*	SNP	*p*				0.70	0.75	0.80	0.85	0.90
TDI	AF	Nielsen et al.	< 1.00E‐03	rs1947083, rs253125, rs990706	5.91E‐01	395,739 (38.39%)	1.06E‐03	35.13	66%	51%	35%	22%	12%
		FinnGen	5.58E‐01	NA	NA	0 (0%)	1.06E‐03	35.13	47%	34%	28%	15%	9%
Household income		Nielsen et al.	1.00E‐03	rs10761035, rs2362523	9.25E‐01	395,739 (38.39%)	2.70E‐03	39.93	97%	88%	71%	47%	24%
		FinnGen	< 1.00E‐03	rs11191116	2.86E‐01	0 (0%)	2.68E‐03	39.61	85%	70%	50%	31%	16%
EA		Nielsen et al.	< 1.00E‐03	rs11172371, rs111821073, rs56409354, rs6502670, rs7650602	6.73E‐01	Up to 395,739 through UKB^a^	1.30E‐02	77.39	100%	100%	100%	98%	78%
		FinnGen	< 1.00E‐03	rs11191193, rs11942352, rs2764684, rs28834466, rs356903, rs4557790, rs4715244, rs5021426, rs7650602, rs78410329	6.03E‐01	Not quantifiable^b^	1.28E‐02	78.12	100%	100%	99%	89%	55%

***Note:*** For the UKB‐based TDI and household‐income GWAS, percentages were calculated relative to the corresponding AF outcome samples. Nielsen et al. included 395,739 UKB participants, accounting for 38.39% of its total sample, whereas FinnGen had no known overlap with UKB.

^a^ The SSGAC EA GWAS included 441,121 UKB participants. Therefore, the potential UKB‐derived overlap with the Nielsen et al. dataset could involve up to 395,739 participants, corresponding to 13.03% of the SSGAC EA sample and 38.39% of the Nielsen et al. sample. Additional overlap involving the other SSGAC cohorts could not be quantified from the publicly available summary‐level information.

^b^ FinnGen had no known cohort‐level overlap with the UKB or 23andMe components of the SSGAC EA GWAS. However, possible overlap with participants from the remaining 69 SSGAC cohorts could not be determined; therefore, zero overlap was not assumed for the EA–FinnGen analysis.

All analyses were performed using the packages TwoSampleMR (version 0.5.6), MR‐PRESSO (version 1.0), and PhenoScanner (version 1.0) in R (version 4.2.2). The threshold for significance was a Bonferroni‐corrected *p*‐value of 2.50E‐02 (correcting for two different outcomes). For all other analyses, a two‐sided *p*‐value of 5.00E‐02 was considered statistically significant.

## 3 Results

### 3.1 MR Estimates

**FIGURE 3:**
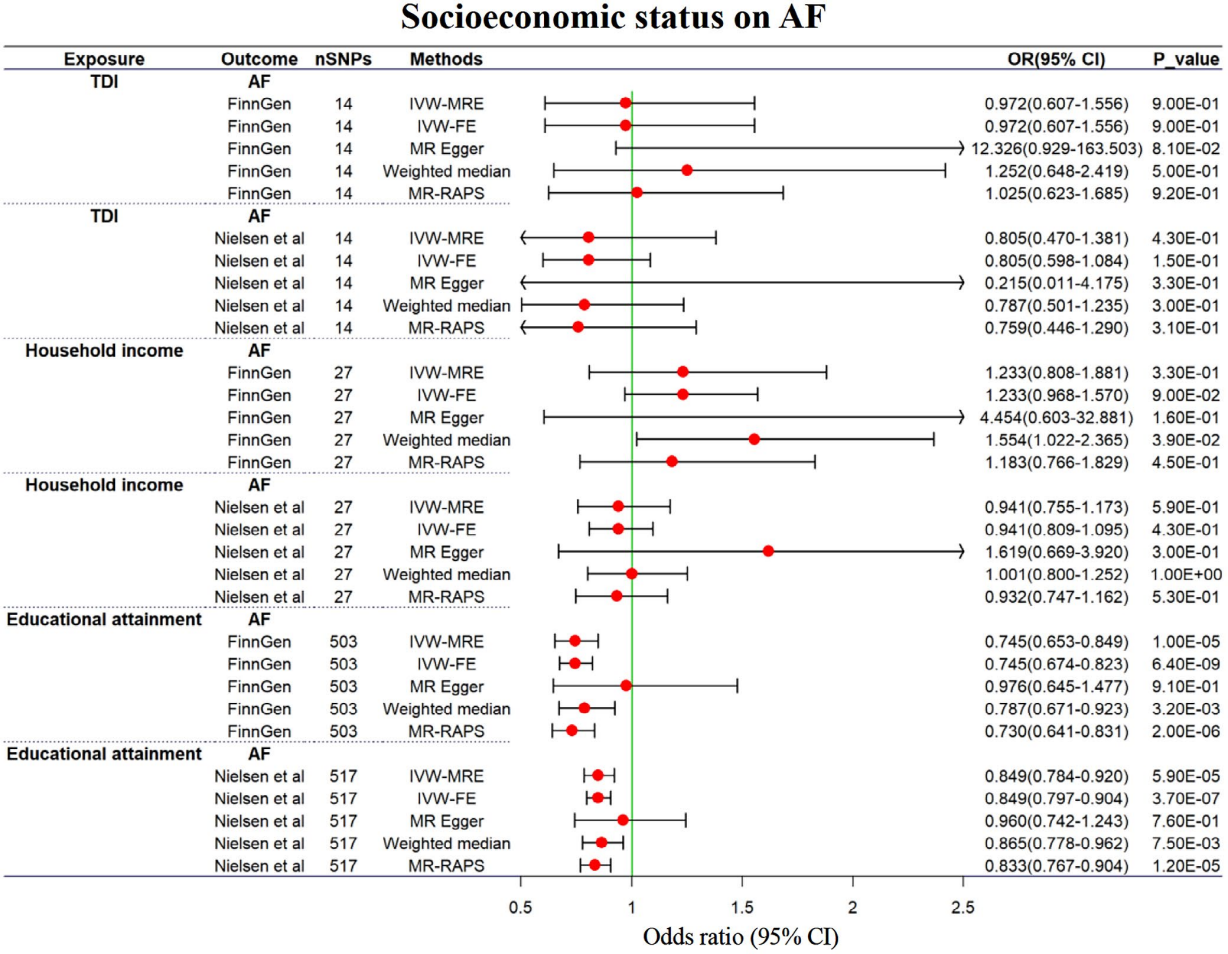
Forest plots showing the MR estimates of the associations between genetically proxied socioeconomic indicators and AF in FinnGen and the study by Nielsen et al. ORs are expressed per one‐SD increase in each genetically proxied exposure. Higher TDI values indicate greater socioeconomic deprivation, whereas higher household income and EA values indicate higher income and greater educational attainment, respectively.

Figure 3 displays the results of all causality analyses conducted in our MR study.

**FIGURE 4:**
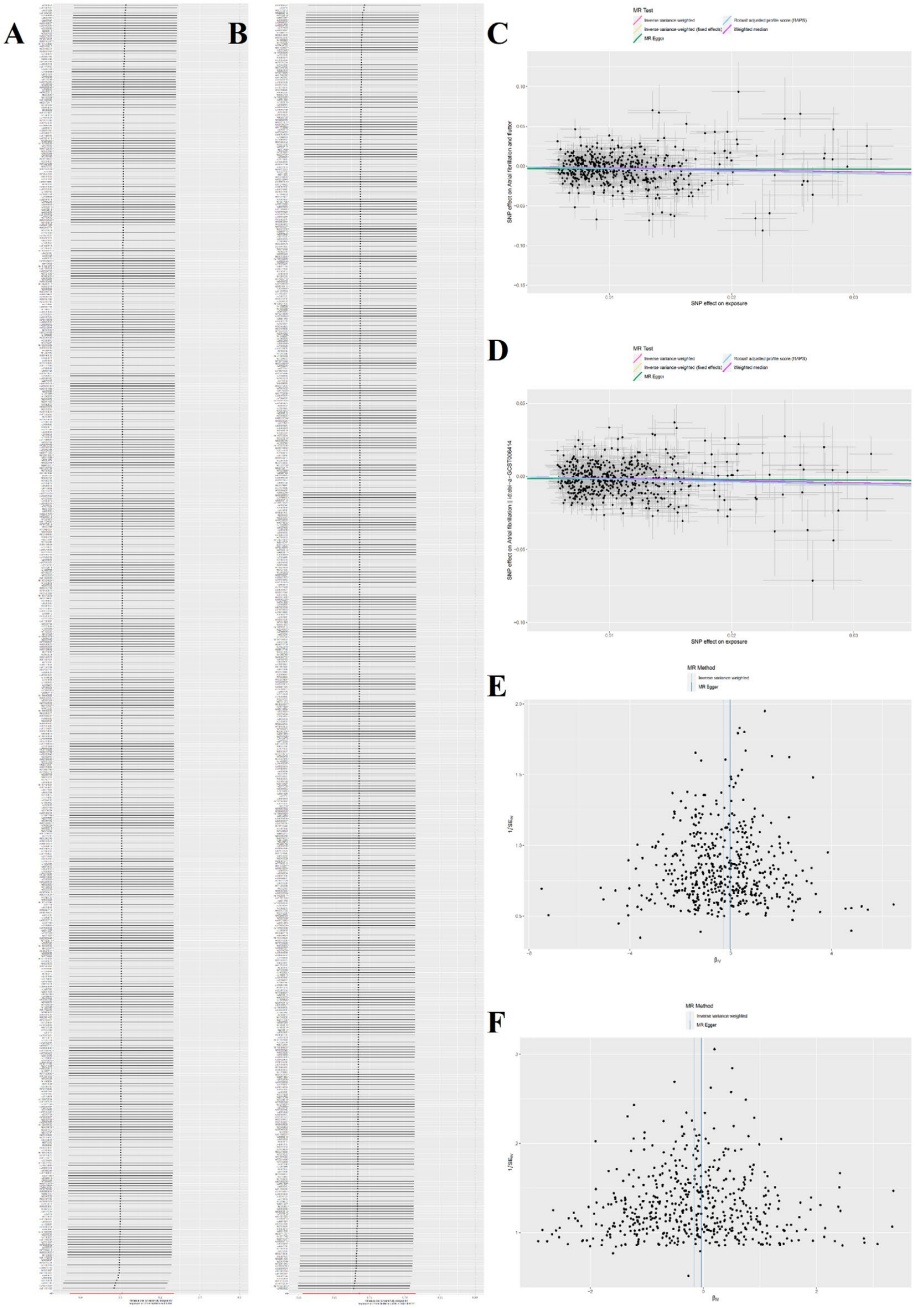
Leave‐one out plots, scatter plots, and funnel plots, to visualize the MR analysis results between the EA and AF both in FinnGen and Nielsen et al. A, B demonstrated the robustness of the results; C, D intuitively showed the causal effect; E, F visualized the heterogeneity through the symmetry of the plot.

Our MR analysis using the primary IVW method revealed no evident association between TDI and AF (in FinnGen: odds ratio [OR] = 0.972, 95% confidence interval [CI] = 0.607–1.556, *p* = 9.00E‐01; in Nielsen et al.: OR = 0.805, 95% CI = 0.470–1.381, *P* = 4.30E‐01). The results did not materially change when other methods were used (MR–Egger, weighted median, and MR–RAPS). The primary IVW‐MRE analysis also showed no evident association between household income and AF (in FinnGen: OR = 1.233, 95% CI = 0.808–1.881, *P* = 3.30E‐01; in Nielsen et al.: OR = 0.941, 95% CI = 0.755–1.173, *p* = 5.90E‐01). In FinnGen, the weighted‐median analysis showed a nominally significant positive association (OR = 1.554, 95% CI = 1.022–2.365, *p* = 3.90E‐02), whereas the IVW‐FE estimate showed a similar direction but did not reach statistical significance (OR = 1.233, 95% CI = 0.968–1.570, *p* = 9.00E‐02). However, the weighted‐median result did not meet the Bonferroni‐corrected significance threshold of *p* < 2.50E‐02 and was not replicated in the Nielsen et al. dataset. By contrast, a distinct result emerged from the EA analysis. In the primary IVW analysis, higher EA was associated with a lower risk of AF in both FinnGen (OR = 0.745, 95% CI = 0.653–0.849, *p* = 1.00E‐05) and the study by Nielsen et al. (OR = 0.849, 95% CI = 0.784–0.920, *p* = 5.90E‐05) (Figure 4C,D). Except for the nominal weighted‐median association for household income in FinnGen, the complementary MR estimates were generally consistent with the primary IVW findings.

### 3.2 Sensitivity Analyses

We also evaluated the validity and robustness of the aforementioned results via a comprehensive sensitivity analysis (Table 2). In our MR study, horizontal pleiotropy was absent because all *p* values in the MR–Egger intercept tests were > 0.05. However, according to the results of Cochran's Q test, our analysis showed heterogeneity, except for TDI and AF in FinnGen (Q = 11.69, *p* = 5.53E‐01). In addition, despite the heterogeneity of some results, the current study's MR estimations as IVW‐MRE, which may counteract the pooled heterogeneity, were not discredited, and most of the funnel plots were symmetrical (Figure 4E,F).

In addition, we performed the MR‐PRESSO method carefully, and through global testing, we discovered a list of outliers. However, after excluding these outliers on the basis of the original analysis, the results did not change considerably (Table 3). We further reviewed the leave‐one‐out plot and observed that the MR estimates were unaffected by SNPs (Figure 4A,B). More details are shown in Figures S1–S4.

### 3.3 Statistical Power

Statistical power was calculated using mRnd based on the sample size, type I error rate (α), proportion of exposure variance explained by the instruments, and prespecified effect sizes (Brion et al. 2013). The results are presented in Table 3.

## 4 Discussion

In our MR study, the primary analyses found no evidence that genetically proxied TDI or household income was associated with AF risk, whereas higher genetically proxied EA was consistently associated with lower AF risk in both outcome datasets. Given the widespread prevalence of AF, early detection and prevention among populations with lower educational attainment. However, the findings should not be interpreted as direct evidence that increasing educational attainment would necessarily reduce AF incidence or hospitalization.

SES represents a measure of one's combined economic and social status. It is typically considered a latent concept in sociology and assessed using a composite measure of occupation, income, and education or combinations of these three indicators (Baker 2014). Likewise, in other reports and papers, SES was usually represented by TDI, household income, and EA (Tyrrell et al. 2016; Campbell et al. 2021).

Importantly, SES is a multidimensional construct encompassing educational, economic, occupational, neighborhood, sociocultural, physical, and environmental dimensions. Therefore, each SES indicator captures a distinct but partially overlapping aspect of socioeconomic circumstances, and no single indicator can comprehensively represent overall SES (Braveman et al. 2005). Accordingly, SNPs associated with EA, household income, or TDI should not be interpreted as genes that directly determine education, income, or deprivation. Rather, they serve as statistical proxies for genetic liability to these complex and socially patterned phenotypes in the populations and environments represented by the source GWAS. Using income‐ or deprivation‐associated SNPs as instrumental variables is methodologically reasonable when the MR assumptions are adequately satisfied, but their interpretation requires particular caution because these traits are less biologically proximal than conventional biomedical exposures.

Household income is a household‐level measure influenced by employment, health, household composition, partner income, and broader economic conditions, whereas TDI is an area‐level measure that may additionally reflect residential selection and neighborhood context. Their genetic associations may therefore operate indirectly through multiple cognitive, non‐cognitive, behavioral, health‐related, and social pathways. Similarly, although EA is genetically correlated with cognitive ability, it is not equivalent to inherited intelligence and is also influenced by non‐cognitive characteristics, family environments, educational institutions, and sociocultural conditions (Okbay et al. 2022). Moreover, GWAS estimates for socioeconomic traits obtained from unrelated individuals may partly capture indirect parental genetic effects, assortative mating, and residual population structure (Brumpton et al. 2020; Howe et al. 2022).

A growing number of epidemiological studies, including cohort studies and meta‐analyses, have investigated the associations between SES and health, with cardiovascular diseases representing an important area of research (Kawada 2019; Schultz et al. 2018). As a frequent cardiovascular condition, AF is also the focus of attention. Most observational studies have suggested that lower SES is associated with an increased risk of AF. A nationwide Danish cohort study from January 1, 1996, to December 31, 2005, included 2,173,857 participants with a median follow‐up of 13.6 years to explore the relationship between SES and AF. After adjustment for relevant covariates, higher SES was associated with a lower risk of being diagnosed with AF among younger individuals (Lunde et al. 2020). Similar results were reported in Australian (Ramkumar et al. 2019) and Italian (Bonaccio et al. 2021) prospective cohort studies. Studies examining specific dimensions of SES further reported that higher TDI was associated with AF development (Han et al. 2022) and poorer prognosis among patients with AF (Lunde et al. 2018). In addition, lower household income and lower EA were associated with an increased risk of AF (Lunde et al. 2020; Goli et al. 2012). Consistent with these findings, patients with AF and lower income or educational attainment were more likely to experience adverse outcomes, including heart failure and myocardial infarction (LaRosa et al. 2020), and had higher mortality (Apiyasawat et al. 2022).

The more consistent association observed for EA in the present study should not necessarily be interpreted as evidence that EA is more directly genetically determined than TDI or household income. The EA GWAS had a substantially larger sample size and provided a larger number of instrumental variables, resulting in greater statistical power, whereas the TDI and household‐income analyses had fewer instruments and lower statistical power (Table 1; Table 3; Tables S1–S6). These methodological differences may partly explain the stronger and more precise EA estimates and indicate that the contrast between the three SES indicators should not be attributed solely to differences in their underlying genetic architecture. Furthermore, the association with genetically proxied EA represents a total effect that may operate through multiple behavioral, cardiometabolic, occupational, and healthcare‐related pathways rather than a purely cognitive or direct biological effect.

With respect to the specific instrument sets used in the present study, 14 TDI‐associated and 27 household‐income‐associated SNPs were retained for both outcome datasets, whereas 503 and 517 EA‐associated SNPs were available for the FinnGen and Nielsen et al. analyses, respectively. Previous functional annotation of income‐associated loci by Hill et al. (Hill et al. 2019) demonstrated links with gene expression, regulatory genomic regions, open‐chromatin states, cortical tissues, and neurotransmission‐related pathways. These findings provide biological context for income‐associated genetic variation but do not establish that each of the 27 household‐income instruments used in our study directly affects income or AF through these pathways because the instruments were selected from a separate source GWAS and were not individually functionally validated in the present analysis. More broadly, the SNPs used for TDI, household income, and EA likely represent multiple upstream pathways contributing to these complex socioeconomic phenotypes rather than direct genetic determinants of socioeconomic position. Nevertheless, exclusion of the outliers identified by MR‐PRESSO did not materially change the estimates, and the leave‐one‐out analyses showed that no individual SNP disproportionately influenced the principal findings.

With respect to household income, a previous MR study also reported no association with AF (Zheng et al. 2023), which is consistent with our primary IVW‐MRE findings. Nevertheless, the weighted‐median analysis in FinnGen yielded a nominally significant positive association between genetically proxied household income and AF risk, while the IVW‐FE estimate showed a similar positive direction. These findings should be interpreted cautiously. Substantial heterogeneity was detected among the SNP‐specific estimates; therefore, the IVW‐MRE model was considered more appropriate than the fixed‐effects model for the primary analysis. Moreover, the weighted‐median association did not survive Bonferroni correction and was neither supported by MR‐RAPS nor replicated in the Nielsen et al. dataset. Thus, the observed positive estimate may reflect estimator‐specific variation rather than a robust causal effect. Further studies using independent datasets are needed to determine whether this potential association can be replicated.

The specific mechanism between SES and the occurrence of AF remains unclear. Some of these potential causes may be explained as follows: (1) SES affects AF by allowing access to resources and therapies that promote health, and (2) early and ongoing socialization of health behaviors varies depending on the SES (Baker 2014).

Conventional observational studies may be affected by residual confounding and reverse causation, while randomized trials of socioeconomic circumstances are generally infeasible. MR can strengthen causal inference by using genetic variants as instrumental variables. Nevertheless, its validity depends on the instrumental‐variable assumptions, and residual bias cannot be completely excluded (Emdin et al. 2017).

MR analysis offers several advantages. First, to our knowledge, this study is the first to investigate the potential causal associations between SES and AF using multiple large‐scale GWAS datasets from different databases. Second, all selected IVs had F‐statistics greater than 10, reducing the likelihood of weak‐instrument bias and supporting adequate instrument strength (Tables S1–S6). Third, restricting the study population to individuals of European genetic ancestry reduced potential bias due to population stratification. Fourth, in addition to the conventional MR analyses, we performed multiple sensitivity analyses and replicated the analyses using two separate AF datasets to evaluate the robustness of our findings.

Several limitations should be acknowledged. First, EA was harmonized across participating cohorts using self‐reported educational qualifications and the age at completion of full‐time education. Therefore, measurement error, recall bias, and potential misclassification of educational attainment cannot be completely excluded. Second, the statistical power for some analyses, particularly those involving TDI and household income, was below 80%. Although all selected instruments had F‐statistics greater than 10, indicating a low likelihood of conventional weak‐instrument bias, adequate instrument strength does not fully compensate for limited statistical power. Therefore, the corresponding null findings should be interpreted cautiously. Third, the present univariable MR analyses estimated the total effect of each SES indicator separately and could not distinguish their mutually adjusted direct effects. We did not perform MVMR because the exposure GWAS datasets had different sample structures and overlapping samples, while the covariance information required to appropriately account for this overlap was unavailable from the publicly accessible summary‐level data. Moreover, only 14 TDI‐associated and 27 household‐income‐associated instruments remained, compared with more than 500 EA‐associated instruments. Given the correlations among the three SES indicators, this imbalance raised concerns regarding insufficient conditional instrument strength and unstable MVMR estimates. Consequently, reliable conditional F‐statistics and stable direct‐effect estimates could not be obtained without unverifiable assumptions. Although PhenoScanner screening and exclusion of SNPs associated with prespecified potential confounders reduced the risk of pleiotropic bias, these procedures cannot replace MVMR. Fourth, potential sample overlap existed between the exposure and outcome GWAS datasets, as detailed in Table 3. For the UKB‐based TDI and household‐income analyses, Nielsen et al. included UKB participants, whereas FinnGen had no overlap with UKB and therefore provided a non‐overlapping replication dataset. For EA, the identifiable potential overlap with Nielsen et al. was primarily attributable to the UKB component of the SSGAC GWAS. However, because the SSGAC EA GWAS also included 69 additional cohorts, additional overlap with Nielsen et al. and possible overlap with FinnGen could not be precisely quantified from the publicly available summary‐level information. We evaluated the identifiable potential UKB overlap using the online sample‐overlap tool (https://sb452.shinyapps.io/overlap/). Under the specified overlap scenario, the estimated type I error rate remained below 0.05. Nevertheless, residual overlap‐related bias, particularly in the EA analyses, cannot be completely excluded, and the corresponding findings should therefore be interpreted with appropriate caution. Fifth, the GWAS datasets predominantly included individuals of European genetic ancestry. Although this restriction reduced potential bias due to population stratification, it limits the generalizability of our findings to populations with other genetic ancestries and different sociocultural backgrounds. Finally, despite the use of PhenoScanner screening and multiple sensitivity analyses, residual horizontal pleiotropy cannot be completely excluded. Future studies using harmonized and appropriately independent datasets may apply MVMR to further disentangle the direct effects of correlated SES dimensions.

In summary, higher genetically proxied EA was associated with lower AF risk, whereas no evidence of association was found for genetically proxied TDI or household income. These findings suggest that populations with lower educational attainment may merit particular attention in future studies evaluating AF risk stratification, early detection, and preventive strategies.

## Author Contributions

S.X. and M.M. designed the study. S.X. and X.Z. analyzed the data. S.X. drafted the manuscript. X.Z. and M.M. reviewed and edited the manuscript. M.M. supervised the study. All authors read and approved the final version of the manuscript.

## Funding

The authors have nothing to report.

## Ethics Statement

As this study utilized publicly accessible databases, no ethical approval or informed consent was necessary.

## Conflicts of Interest

The authors declare no conflicts of interest.

## Supporting information


**Figure S1:** Scatter plot to visualize the effect size for each SNP on SES and AF: (A) TDI and AF in Nielsen et al.; (B) TDI and AF in FinnGen; (C) household income and AF in Nielsen et al.; (D) household income and AF in FinnGen.
**Figure S2:** MR leave‐one‐out sensitivity analysis to visualize the robustness of the results: (A) TDI and AF in Nielsen et al.; (B) TDI and AF in FinnGen; (C) household income and AF in Nielsen et al.; (D) household income and AF in FinnGen.
**Figure S3:** Funnel plot to visualize the heterogeneity: (A) TDI and AF in Nielsen et al.; (B) TDI and AF in FinnGen; (C) household income and AF in Nielsen et al.; (D) household income and AF in FinnGen.
**Figure S4:** Forest plot to visualize the causal effect between SES and AF: (A) TDI and AF in Nielsen et al.; (B) TDI and AF in FinnGen; (C) household income and AF in Nielsen et al.; (D) household income and AF in FinnGen; (E) EA and AF in Nielsen et al.; (F) EA and AF in FinnGen.

**Table S1:** Characteristics of Townsend Deprivation Index‐associated SNPs for IVs to atrial fibrillation in FinnGen. SNP, Single nucleotide polymorphism; Pos, position for SNP; Chr, chromosome; SE, standard error; EAF, effect allele frequency; *p*‐value, the value for the genetic association.
**Table S2:** Characteristics of house income‐associated SNPs for IVs to atrial fibrillation in FinnGen. SNP, Single nucleotide polymorphism; Pos, position for SNP; Chr, chromosome; SE, standard error; EAF, effect allele frequency; *p*‐value, the value for the genetic association.
**Table S3:** Characteristics of educational attainment‐associated SNPs for IVs to atrial fibrillation in FinnGen. SNP, Single nucleotide polymorphism; Pos, position for SNP; Chr, chromosome; SE, standard error; EAF, effect allele frequency; *p*‐value, the value for the genetic association.
**Table S4:** Characteristics of Townsend Deprivation Index‐associated SNPs for IVs to atrial fibrillation in Nielsen et al. SNP, Single nucleotide polymorphism; Pos, position for SNP; Chr, chromosome; SE, standard error; EAF, effect allele frequency; *p*‐value, the value for the genetic association.
**Table S5:** Characteristics of house income‐associated SNPs for IVs to atrial fibrillation in Nielsen et al. SNP, Single nucleotide polymorphism; Pos, position for SNP; Chr, chromosome; SE, standard error; EAF, effect allele frequency; *p*‐value, the value for the genetic association.
**Table S6:** Characteristics of educational attainment‐associated SNPs for IVs to atrial fibrillation in Nielsen et al. SNP, Single nucleotide polymorphism; Pos, position for SNP; Chr, chromosome; SE, standard error; EAF, effect allele frequency; *p*‐value, the value for the genetic association.

## Data Availability

The data that support the findings of this study are openly available in FinnGen, MRC‐IEU, Nielsen et al. study and SSGAC at finngen_access_results, gwas_mrcieu, Atrial Fibrillation Summary Statistics, and thessgac_datas.
