# Supplementary material for: Association Between Socioeconomic Status and Atrial Fibrillation: A Two‐Sample Mendelian Randomization Study
Source: Ann Noninvasive Electrocardiol. 2026 Aug 3;31(5):e70227. doi: 10.1111/anec.70227 (PMC13434036; doi:10.1111/anec.70227)
Supplement: Supplementary file 1 [file ANEC-31-e70227-s002.pdf]

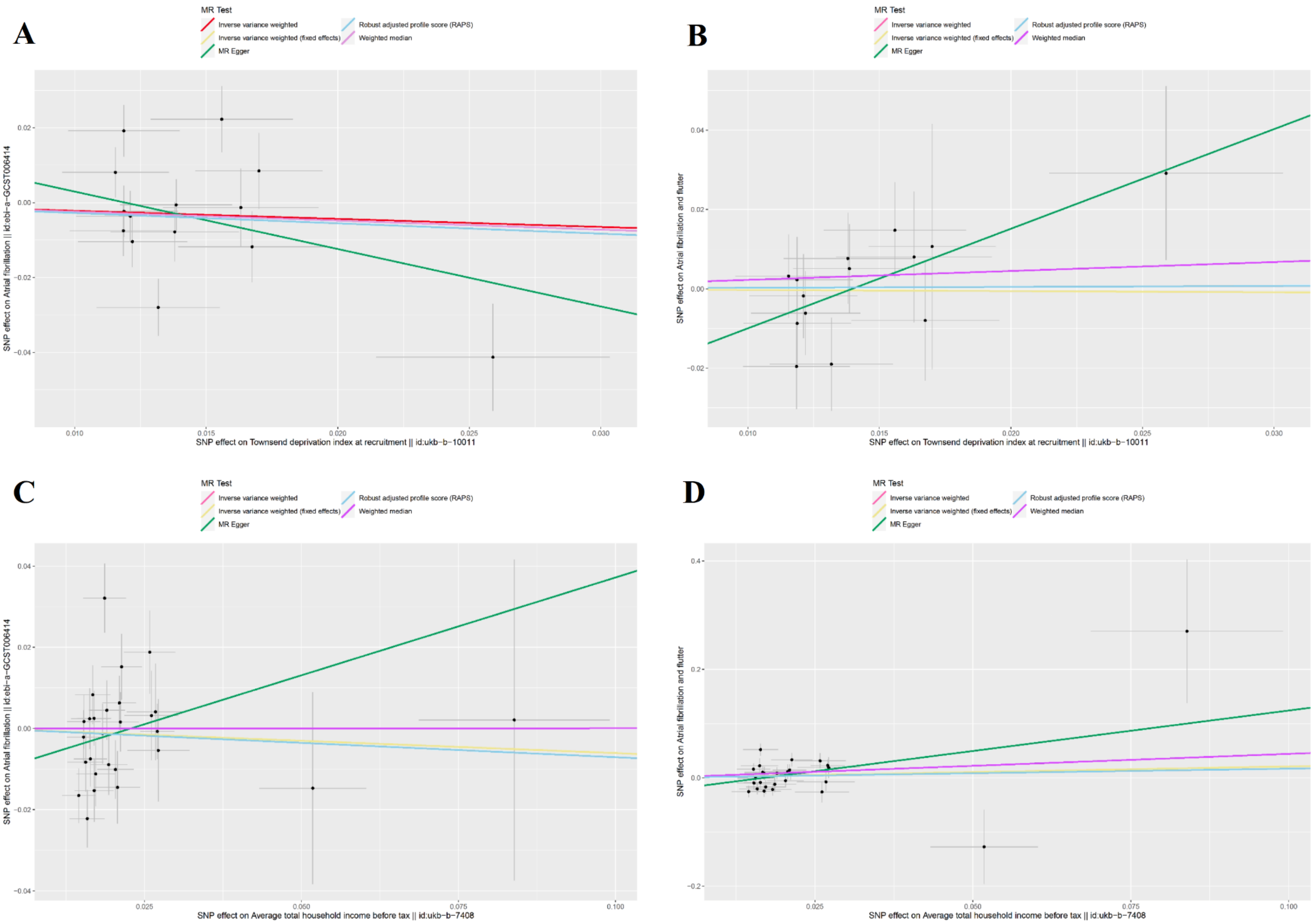

Supplementary Figure S1. Scatter plot to visualize the effect size for each SNP on SES and AF: (A) TDI and AF in Nielsen et al; (B) TDI and AF in FinnGen; (C) household income and AF in Nielsen et al; (D) household income and AF in FinnGen.

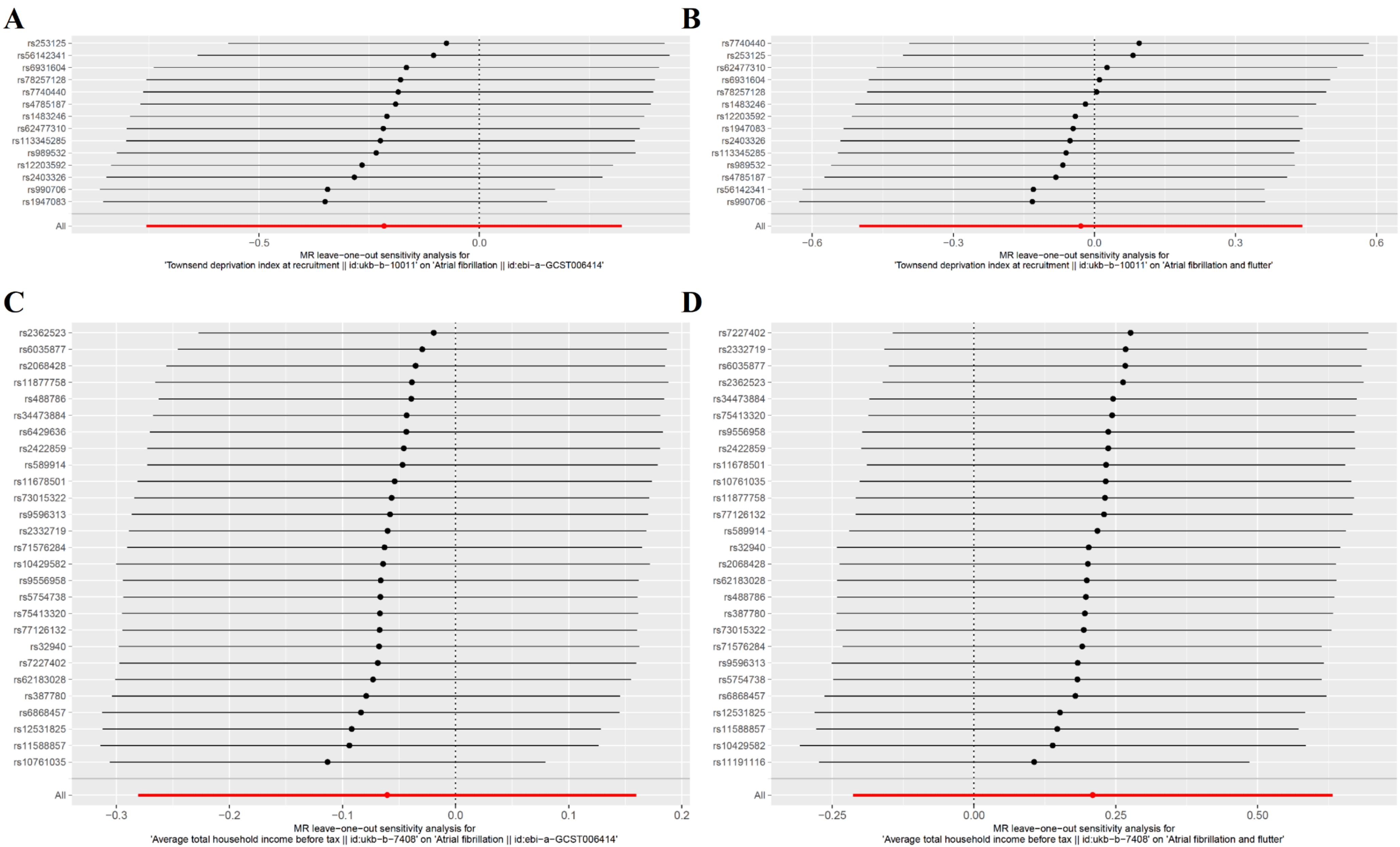

Supplementary Figure S2. MR leave-one-out sensitivity analysis to visualize the robustness of the results: (A) TDI and AF in Nielsen et al; (B) TDI and AF in FinnGen; (C) household income and AF in Nielsen et al; (D) household income and AF in FinnGen.

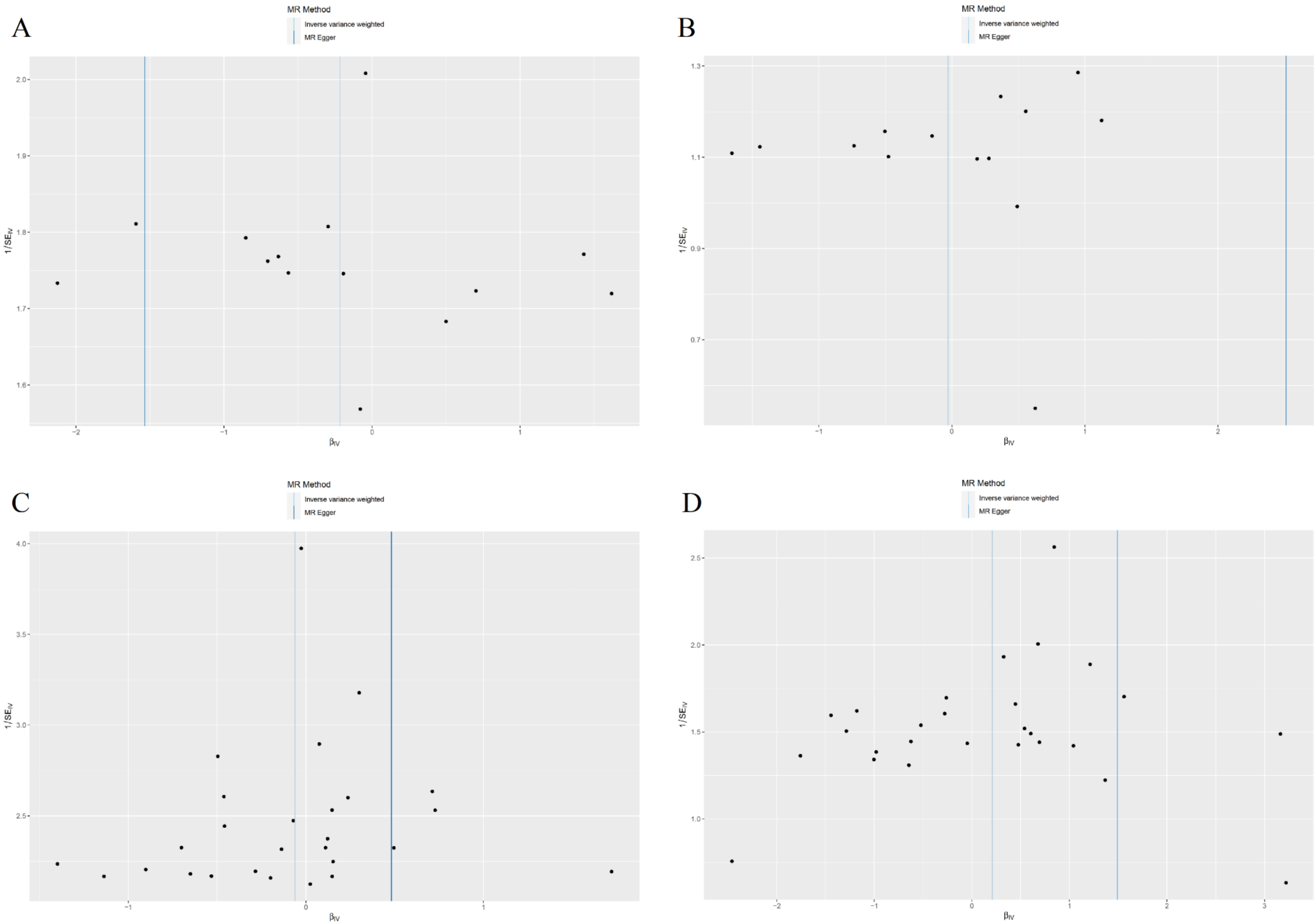

Supplementary Figure S3. Funnel plot to visualize the heterogeneity: (A) TDI and AF in Nielsen et al; (B) TDI and AF in FinnGen; (C) household income and AF in Nielsen et al; (D) household income and AF in FinnGen.

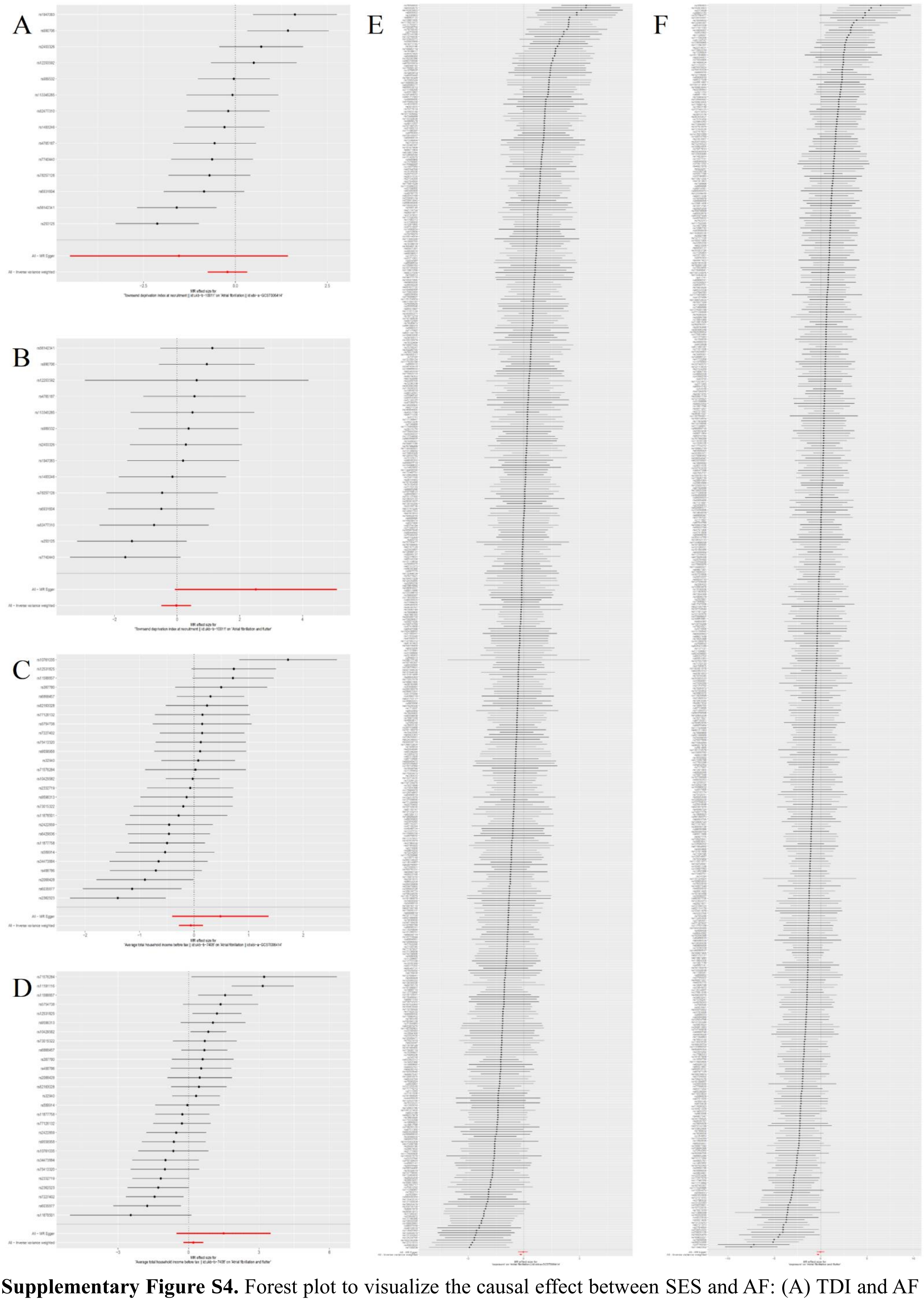

Supplementary Figure S4. Forest plot to visualize the causal effect between SES and AF: (A) TDI and AF in Nielsen et al; (B) TDI and AF in FinnGen; (C) household income and AF in Nielsen et al; (D) household income and AF in FinnGen; (E) EA and AF in Nielsen et al; (F) EA and AF in FinnGen.
