# Supplementary material for: Association Between Socioeconomic Status and Atrial Fibrillation: A Two‐Sample Mendelian Randomization Study
Source: Ann Noninvasive Electrocardiol. 2026 Aug 3;31(5):e70227. doi: 10.1111/anec.70227 (PMC13434036; doi:10.1111/anec.70227)
Supplement: Supplementary file 2 [file ANEC-31-e70227-s001.docx]

**Supplementary Table S1.** Characteristics of Townsend Deprivation Index-associated SNPs for IVs to atrial fibrillation in FinnGen

| SNP | Chr | Pos | Beta | SE | Sample  size | Effect allele | Other allele | EAF | P_value | R2 | F |
| --- | --- | --- | --- | --- | --- | --- | --- | --- | --- | --- | --- |
| rs1947083 | 1 | 67004801 | 0.0118662 | 0.0021105 | 462464 | A | G | 0.641804 | 1.90E-08 | 6.84E-05 | 31.61 |
| rs56142341 | 1 | 208145995 | -0.0258973 | 0.00443347 | 462464 | C | G | 0.062465 | 5.20E-09 | 7.38E-05 | 34.12 |
| rs989532 | 2 | 173933824 | 0.0138559 | 0.00212756 | 462464 | G | A | 0.353176 | 7.40E-11 | 9.17E-05 | 42.41 |
| rs1483246 | 2 | 186050380 | -0.0121102 | 0.00204409 | 462464 | C | T | 0.555268 | 3.10E-09 | 7.59E-05 | 35.1 |
| rs78257128 | 5 | 164545854 | -0.0167416 | 0.00280676 | 462464 | T | C | 0.154162 | 2.40E-09 | 7.69E-05 | 35.58 |
| rs2403326 | 5 | 103791044 | 0.0115465 | 0.00202703 | 462464 | G | A | 0.486226 | 1.20E-08 | 7.02E-05 | 32.45 |
| rs253125 | 5 | 106645685 | 0.013174 | 0.0023432 | 462464 | C | T | 0.749502 | 1.90E-08 | 6.83E-05 | 31.61 |
| rs7740440 | 6 | 142972917 | 0.011847 | 0.00203391 | 462464 | A | G | 0.487004 | 5.70E-09 | 7.34E-05 | 33.93 |
| rs12203592 | 6 | 396321 | 0.0170008 | 0.00240989 | 462464 | T | C | 0.219642 | 1.70E-12 | 1.08E-04 | 49.77 |
| rs6931604 | 6 | 98578215 | -0.0121903 | 0.00207309 | 462464 | T | C | 0.600998 | 4.10E-09 | 7.48E-05 | 34.58 |
| rs62477310 | 7 | 114951541 | -0.011872 | 0.00203538 | 462464 | C | T | 0.486627 | 5.40E-09 | 7.36E-05 | 34.02 |
| rs990706 | 11 | 78135704 | 0.0155869 | 0.00270141 | 462464 | T | C | 0.171143 | 7.90E-09 | 7.20E-05 | 33.29 |
| rs113345285 | 14 | 104246362 | 0.0163127 | 0.00295094 | 462464 | C | T | 0.136874 | 3.20E-08 | 6.61E-05 | 30.56 |
| rs4785187 | 16 | 49766772 | 0.0137996 | 0.00243703 | 462464 | A | G | 0.223024 | 1.50E-08 | 6.93E-05 | 32.06 |

**SNP**, Single nucleotide polymorphism; **Pos**, position for SNP; **Chr**, chromosome; **SE**, standard error; **EAF**, effect allele frequency; **P-value**, the value for the genetic association.

**Supplementary Table S2.** Characteristics of house income-associated SNPs for IVs to atrial fibrillation in FinnGen

| SNP | Chr | Pos | Beta | SE | Sample  size | Effect allele | Other allele | EAF | P_value | R2 | F |
| --- | --- | --- | --- | --- | --- | --- | --- | --- | --- | --- | --- |
| rs11588857 | 1 | 2.05E+08 | 0.02134 | 0.003246 | 397751 | A | G | 0.209469 | 4.90E-11 | 1.09E-04 | 43.22 |
| rs488786 | 1 | 20888207 | 0.020688 | 0.003568 | 397751 | T | C | 0.164058 | 6.70E-09 | 8.45E-05 | 33.63 |
| rs387780 | 2 | 32502495 | 0.01673 | 0.002827 | 397751 | C | T | 0.674498 | 3.30E-09 | 8.80E-05 | 35.01 |
| rs11678501 | 2 | 1.89E+08 | -0.05179 | 0.008516 | 397751 | C | T | 0.024727 | 1.20E-09 | 9.30E-05 | 36.98 |
| rs62183028 | 2 | 2.13E+08 | -0.01898 | 0.002861 | 397751 | T | G | 0.310562 | 3.20E-11 | 1.11E-04 | 44.02 |
| rs2332719 | 3 | 1.24E+08 | -0.0183 | 0.002953 | 397751 | G | A | 0.278917 | 5.70E-10 | 9.66E-05 | 38.42 |
| rs32940 | 5 | 1.41E+08 | 0.021143 | 0.002896 | 397751 | C | T | 0.700979 | 2.90E-13 | 1.34E-04 | 53.28 |
| rs6868457 | 5 | 60550041 | 0.02098 | 0.00266 | 397751 | C | T | 0.478238 | 3.10E-15 | 1.56E-04 | 62.2 |
| rs73015322 | 6 | 1.64E+08 | -0.02719 | 0.004954 | 397751 | T | G | 0.076734 | 4.10E-08 | 7.57E-05 | 30.12 |
| rs12531825 | 7 | 8005174 | -0.02582 | 0.004062 | 397751 | A | G | 0.122596 | 2.10E-10 | 1.02E-04 | 40.4 |
| rs77126132 | 7 | 54966738 | 0.026748 | 0.004579 | 397751 | A | G | 0.093289 | 5.20E-09 | 8.58E-05 | 34.12 |
| rs71576284 | 7 | 1.26E+08 | -0.08389 | 0.015177 | 397751 | A | C | 0.008961 | 3.30E-08 | 7.68E-05 | 30.55 |
| rs10761035 | 9 | 99236092 | 0.01864 | 0.003385 | 397751 | A | G | 0.188004 | 3.70E-08 | 7.62E-05 | 30.32 |
| rs2068428 | 9 | 1792147 | 0.016972 | 0.003095 | 397751 | T | C | 0.239041 | 4.20E-08 | 7.56E-05 | 30.07 |
| rs10429582 | 9 | 23346850 | 0.027026 | 0.002684 | 397751 | C | T | 0.416388 | 7.60E-24 | 2.55E-04 | 101.39 |
| rs34473884 | 10 | 1.34E+08 | 0.017225 | 0.003056 | 397751 | A | G | 0.248977 | 1.70E-08 | 7.99E-05 | 31.77 |
| rs11191116 | 10 | 1.04E+08 | -0.0164 | 0.002776 | 397751 | T | C | 0.351782 | 3.50E-09 | 8.77E-05 | 34.89 |
| rs589914 | 11 | 57661032 | 0.01561 | 0.002851 | 397751 | G | A | 0.684776 | 4.40E-08 | 7.54E-05 | 29.98 |
| rs9596313 | 13 | 51150873 | 0.015287 | 0.002648 | 397751 | A | G | 0.505975 | 7.80E-09 | 8.38E-05 | 33.34 |
| rs9556958 | 13 | 99100046 | -0.01534 | 0.002664 | 397751 | T | C | 0.524062 | 8.50E-09 | 8.34E-05 | 33.16 |
| rs11877758 | 18 | 35138110 | -0.02036 | 0.002867 | 397751 | G | T | 0.312055 | 1.20E-12 | 1.27E-04 | 50.43 |
| rs7227402 | 18 | 50895766 | -0.01696 | 0.002678 | 397751 | T | G | 0.423669 | 2.40E-10 | 1.01E-04 | 40.11 |
| rs75413320 | 19 | 13149854 | -0.02611 | 0.004268 | 397751 | C | T | 0.108161 | 9.50E-10 | 9.41E-05 | 37.43 |
| rs2362523 | 19 | 5002301 | 0.015867 | 0.002798 | 397751 | G | A | 0.339926 | 1.40E-08 | 8.08E-05 | 32.15 |
| rs6035877 | 20 | 21512532 | -0.01451 | 0.00265 | 397751 | C | A | 0.469244 | 4.30E-08 | 7.54E-05 | 29.99 |
| rs2422859 | 20 | 3132828 | 0.016371 | 0.002647 | 397751 | G | T | 0.471922 | 6.20E-10 | 9.61E-05 | 38.25 |
| rs5754738 | 22 | 34280249 | -0.01625 | 0.00292 | 397751 | G | A | 0.709551 | 2.70E-08 | 7.78E-05 | 30.95 |

**SNP**, Single nucleotide polymorphism; **Pos**, position for SNP; **Chr**, chromosome; **SE**, standard error; **EAF**, effect allele frequency; **P-value**, the value for the genetic association.

**Supplementary Table S3.** Characteristics of educational attainment-associated SNPs for IVs to atrial fibrillation in FinnGen

| SNP | Beta | SE | Sample  size | Effect allele | Other allele | EAF | P_value | R2 | F |
| --- | --- | --- | --- | --- | --- | --- | --- | --- | --- |
| rs114440021 | 0.01233 | 0.00165 | 3037499 | T | C | 0.111056 | 7.00E-14 | 1.84E-05 | 55.84 |
| rs899223 | 0.01332 | 0.00193 | 3037499 | C | G | 0.078673 | 5.07E-12 | 1.57E-05 | 47.63 |
| rs421154 | 0.01098 | 0.00125 | 3037499 | C | G | 0.781367 | 2.06E-18 | 2.54E-05 | 77.16 |
| rs4766975 | -0.00871 | 0.00107 | 3037499 | A | G | 0.61526 | 4.40E-16 | 2.18E-05 | 66.26 |
| rs6539284 | -0.00721 | 0.00107 | 3037499 | T | C | 0.612997 | 1.38E-11 | 1.49E-05 | 45.4 |
| rs6563363 | -0.00921 | 0.00108 | 3037499 | T | G | 0.357053 | 1.70E-17 | 2.39E-05 | 72.72 |
| rs74740938 | 0.01208 | 0.00162 | 3037499 | A | T | 0.132445 | 8.30E-14 | 1.83E-05 | 55.6 |
| rs7301232 | -0.00773 | 0.0014 | 3037499 | A | T | 0.167863 | 3.80E-08 | 1.00E-05 | 30.49 |
| rs9551887 | -0.008 | 0.00105 | 3037499 | T | C | 0.531029 | 2.88E-14 | 1.91E-05 | 58.05 |
| rs11746390 | -0.0086 | 0.00118 | 3037499 | T | C | 0.742439 | 3.28E-13 | 1.75E-05 | 53.12 |
| rs211283 | -0.00922 | 0.00123 | 3037499 | C | G | 0.233123 | 5.18E-14 | 1.85E-05 | 56.19 |
| rs11633934 | 0.00803 | 0.00105 | 3037499 | A | C | 0.549938 | 2.10E-14 | 1.93E-05 | 58.49 |
| rs57214198 | -0.0155 | 0.00236 | 3037499 | T | G | 0.055159 | 5.47E-11 | 1.42E-05 | 43.14 |
| rs16919774 | -0.00935 | 0.00128 | 3037499 | A | T | 0.794657 | 2.67E-13 | 1.76E-05 | 53.36 |
| rs12524795 | 0.00851 | 0.00105 | 3037499 | T | C | 0.438482 | 6.32E-16 | 2.16E-05 | 65.69 |
| rs7002655 | 0.00692 | 0.00105 | 3037499 | A | G | 0.446135 | 5.12E-11 | 1.43E-05 | 43.43 |
| rs10180485 | 0.01188 | 0.0016 | 3037499 | T | G | 0.882753 | 1.29E-13 | 1.81E-05 | 55.13 |
| rs7070545 | -0.00624 | 0.00108 | 3037499 | A | G | 0.639067 | 8.89E-09 | 1.10E-05 | 33.38 |
| rs7924465 | 0.01448 | 0.0018 | 3037499 | T | C | 0.909409 | 8.50E-16 | 2.13E-05 | 64.71 |
| rs2055940 | 0.00703 | 0.00112 | 3037499 | A | G | 0.307207 | 3.32E-10 | 1.30E-05 | 39.4 |
| rs11942352 | -0.00894 | 0.00132 | 3037499 | A | G | 0.814075 | 1.40E-11 | 1.51E-05 | 45.87 |
| rs12065238 | 0.01009 | 0.00139 | 3037499 | A | G | 0.179812 | 4.57E-13 | 1.73E-05 | 52.69 |
| rs12142570 | 0.00999 | 0.0014 | 3037499 | A | T | 0.818525 | 1.08E-12 | 1.68E-05 | 50.92 |
| rs12974657 | 0.00917 | 0.00113 | 3037499 | T | C | 0.677318 | 3.71E-16 | 2.17E-05 | 65.85 |
| rs1866823 | 0.01058 | 0.00105 | 3037499 | A | G | 0.54247 | 8.10E-24 | 3.34E-05 | 101.53 |
| rs4741600 | 0.0158 | 0.0011 | 3037499 | T | C | 0.344949 | 2.09E-46 | 6.79E-05 | 206.31 |
| rs11614957 | 0.01206 | 0.00105 | 3037499 | A | G | 0.450693 | 2.82E-30 | 4.34E-05 | 131.92 |
| rs2222760 | -0.00794 | 0.00116 | 3037499 | A | G | 0.27219 | 8.24E-12 | 1.54E-05 | 46.85 |
| rs12240387 | -0.00599 | 0.00109 | 3037499 | A | G | 0.356391 | 3.59E-08 | 9.94E-06 | 30.2 |
| rs252991 | 0.01026 | 0.00108 | 3037499 | A | G | 0.369464 | 2.33E-21 | 2.97E-05 | 90.25 |
| rs2025258 | 0.00668 | 0.0011 | 3037499 | T | C | 0.657299 | 1.23E-09 | 1.21E-05 | 36.88 |
| rs8058137 | 0.01466 | 0.00127 | 3037499 | A | G | 0.772806 | 8.61E-31 | 4.39E-05 | 133.25 |
| rs660010 | 0.01863 | 0.0015 | 3037499 | C | G | 0.123945 | 3.24E-35 | 5.08E-05 | 154.26 |
| rs13332638 | -0.01304 | 0.00187 | 3037499 | A | G | 0.091777 | 3.12E-12 | 1.60E-05 | 48.63 |
| rs10752262 | 0.00932 | 0.00106 | 3037499 | T | C | 0.408516 | 2.08E-18 | 2.55E-05 | 77.31 |
| rs11158200 | 0.00861 | 0.00105 | 3037499 | A | G | 0.540468 | 2.53E-16 | 2.21E-05 | 67.24 |
| rs8072494 | -0.0116 | 0.00128 | 3037499 | A | G | 0.788728 | 1.65E-19 | 2.70E-05 | 82.13 |
| rs72906124 | 0.01836 | 0.00226 | 3037499 | T | C | 0.948337 | 4.94E-16 | 2.17E-05 | 66 |
| rs7027899 | 0.01137 | 0.00193 | 3037499 | A | G | 0.80907 | 3.80E-09 | 1.14E-05 | 34.71 |
| rs11191193 | 0.0163 | 0.0011 | 3037499 | A | G | 0.672698 | 1.42E-49 | 7.23E-05 | 219.58 |
| rs14184 | -0.01343 | 0.00114 | 3037499 | C | G | 0.702756 | 6.96E-32 | 4.57E-05 | 138.78 |
| rs2195041 | 0.00659 | 0.00107 | 3037499 | A | G | 0.392886 | 6.16E-10 | 1.25E-05 | 37.93 |
| rs4391653 | -0.00816 | 0.00119 | 3037499 | T | C | 0.254173 | 6.89E-12 | 1.55E-05 | 47.02 |
| rs12569163 | -0.01233 | 0.00183 | 3037499 | T | G | 0.800092 | 1.69E-11 | 1.49E-05 | 45.4 |
| rs7603132 | 0.01729 | 0.00134 | 3037499 | A | G | 0.183585 | 2.70E-38 | 5.48E-05 | 166.49 |
| rs4751360 | 0.0091 | 0.00105 | 3037499 | T | C | 0.468987 | 4.01E-18 | 2.47E-05 | 75.11 |
| rs12609965 | 0.00706 | 0.00122 | 3037499 | A | C | 0.245504 | 8.05E-09 | 1.10E-05 | 33.49 |
| rs6534704 | -0.015 | 0.00189 | 3037499 | A | T | 0.073221 | 1.96E-15 | 2.07E-05 | 62.99 |
| rs10772644 | 0.01397 | 0.00168 | 3037499 | C | G | 0.886665 | 1.05E-16 | 2.28E-05 | 69.15 |
| rs11081529 | 0.01228 | 0.00117 | 3037499 | T | C | 0.7247 | 7.10E-26 | 3.63E-05 | 110.16 |
| rs705240 | -0.01301 | 0.00135 | 3037499 | T | C | 0.181183 | 4.56E-22 | 3.06E-05 | 92.87 |
| rs4551987 | 0.01248 | 0.00142 | 3037499 | A | G | 0.839436 | 1.62E-18 | 2.54E-05 | 77.24 |
| rs4685448 | -0.00679 | 0.00105 | 3037499 | A | G | 0.421497 | 1.20E-10 | 1.38E-05 | 41.82 |
| rs13284474 | 0.00635 | 0.00105 | 3037499 | T | C | 0.427502 | 1.72E-09 | 1.20E-05 | 36.57 |
| rs10016110 | 0.00947 | 0.00108 | 3037499 | A | G | 0.369587 | 1.44E-18 | 2.53E-05 | 76.89 |
| rs56188374 | -0.01411 | 0.00193 | 3037499 | A | G | 0.070142 | 2.72E-13 | 1.76E-05 | 53.45 |
| rs2289769 | -0.00838 | 0.00151 | 3037499 | A | G | 0.869002 | 2.83E-08 | 1.01E-05 | 30.8 |
| rs41741 | 0.00737 | 0.00111 | 3037499 | T | G | 0.663074 | 3.35E-11 | 1.45E-05 | 44.08 |
| rs6044142 | -0.00739 | 0.00106 | 3037499 | A | G | 0.584339 | 2.65E-12 | 1.60E-05 | 48.6 |
| rs5759002 | 0.00832 | 0.00105 | 3037499 | A | G | 0.507638 | 1.98E-15 | 2.07E-05 | 62.79 |
| rs9869597 | -0.00873 | 0.00128 | 3037499 | A | T | 0.782538 | 7.46E-12 | 1.53E-05 | 46.52 |
| rs35565533 | 0.00842 | 0.00119 | 3037499 | A | C | 0.744225 | 1.22E-12 | 1.65E-05 | 50.06 |
| rs67829508 | -0.0092 | 0.00136 | 3037499 | A | G | 0.175978 | 1.16E-11 | 1.51E-05 | 45.76 |
| rs111821073 | 0.01533 | 0.00145 | 3037499 | T | C | 0.149061 | 4.70E-26 | 3.68E-05 | 111.78 |
| rs62262312 | 0.01889 | 0.00142 | 3037499 | C | G | 0.150724 | 2.36E-40 | 5.83E-05 | 176.96 |
| rs1337731 | 0.00863 | 0.00111 | 3037499 | A | G | 0.340345 | 7.07E-15 | 1.99E-05 | 60.45 |
| rs2301015 | -0.01065 | 0.00112 | 3037499 | T | C | 0.341084 | 1.37E-21 | 2.98E-05 | 90.42 |
| rs4772268 | 0.00992 | 0.00111 | 3037499 | A | G | 0.332799 | 4.03E-19 | 2.63E-05 | 79.87 |
| rs7632819 | -0.01301 | 0.00122 | 3037499 | A | G | 0.76138 | 1.13E-26 | 3.74E-05 | 113.72 |
| rs13252156 | 0.00899 | 0.00107 | 3037499 | A | C | 0.416954 | 4.69E-17 | 2.32E-05 | 70.59 |
| rs1182532 | 0.01107 | 0.00146 | 3037499 | A | C | 0.145642 | 4.00E-14 | 1.89E-05 | 57.49 |
| rs11172371 | -0.0107 | 0.00112 | 3037499 | T | C | 0.313705 | 1.23E-21 | 3.00E-05 | 91.27 |
| rs10167909 | 0.00897 | 0.00127 | 3037499 | T | C | 0.197244 | 1.98E-12 | 1.64E-05 | 49.89 |
| rs118134876 | -0.03109 | 0.00219 | 3037499 | T | C | 0.053449 | 6.97E-46 | 6.63E-05 | 201.54 |
| rs9374194 | -0.00881 | 0.00133 | 3037499 | T | C | 0.808593 | 4.06E-11 | 1.44E-05 | 43.88 |
| rs567775 | 0.00697 | 0.00105 | 3037499 | T | C | 0.476794 | 2.73E-11 | 1.45E-05 | 44.06 |
| rs115124024 | 0.01783 | 0.00241 | 3037499 | A | C | 0.952649 | 1.47E-13 | 1.80E-05 | 54.74 |
| rs7109373 | 0.01041 | 0.00132 | 3037499 | A | T | 0.805929 | 2.90E-15 | 2.05E-05 | 62.19 |
| rs12699131 | 0.01382 | 0.00105 | 3037499 | A | G | 0.471158 | 1.05E-39 | 5.70E-05 | 173.24 |
| rs11861256 | 0.01004 | 0.00133 | 3037499 | A | C | 0.191839 | 4.73E-14 | 1.88E-05 | 56.99 |
| rs8020023 | 0.01445 | 0.00123 | 3037499 | A | G | 0.247182 | 6.34E-32 | 4.54E-05 | 138.01 |
| rs4141463 | -0.00864 | 0.00106 | 3037499 | T | C | 0.429104 | 3.90E-16 | 2.19E-05 | 66.44 |
| rs7296742 | -0.00855 | 0.00116 | 3037499 | A | G | 0.274145 | 2.06E-13 | 1.79E-05 | 54.33 |
| rs77584294 | 0.02591 | 0.00268 | 3037499 | A | C | 0.03725 | 3.86E-22 | 3.08E-05 | 93.47 |
| rs12908446 | 0.00726 | 0.00128 | 3037499 | T | C | 0.780828 | 1.59E-08 | 1.06E-05 | 32.17 |
| rs6747129 | -0.01568 | 0.00173 | 3037499 | A | G | 0.119495 | 1.14E-19 | 2.70E-05 | 82.15 |
| rs12339610 | 0.00904 | 0.0012 | 3037499 | T | C | 0.745827 | 4.08E-14 | 1.87E-05 | 56.75 |
| rs78410329 | 0.01783 | 0.00126 | 3037499 | T | C | 0.237404 | 1.63E-45 | 6.59E-05 | 200.24 |
| rs2303907 | 0.00852 | 0.00105 | 3037499 | T | C | 0.517847 | 4.15E-16 | 2.17E-05 | 65.84 |
| rs9540920 | 0.00584 | 0.00106 | 3037499 | A | G | 0.578703 | 3.38E-08 | 9.99E-06 | 30.35 |
| rs2974337 | 0.0118 | 0.00105 | 3037499 | T | C | 0.478134 | 2.42E-29 | 4.16E-05 | 126.29 |
| rs7977614 | -0.01115 | 0.00118 | 3037499 | A | G | 0.725085 | 2.53E-21 | 2.94E-05 | 89.29 |
| rs1464018 | -0.01361 | 0.00128 | 3037499 | T | C | 0.245288 | 2.81E-26 | 3.72E-05 | 113.06 |
| rs6721505 | 0.00736 | 0.00112 | 3037499 | A | G | 0.324669 | 4.09E-11 | 1.42E-05 | 43.18 |
| rs75756477 | 0.01406 | 0.00255 | 3037499 | T | C | 0.961287 | 3.49E-08 | 1.00E-05 | 30.4 |
| rs10745307 | -0.01069 | 0.00105 | 3037499 | A | G | 0.564829 | 2.85E-24 | 3.41E-05 | 103.65 |
| rs78116078 | 0.0093 | 0.00116 | 3037499 | C | G | 0.726332 | 1.28E-15 | 2.12E-05 | 64.28 |
| rs73210523 | 0.01184 | 0.00138 | 3037499 | A | G | 0.164906 | 9.72E-18 | 2.42E-05 | 73.61 |
| rs12828220 | -0.00975 | 0.00109 | 3037499 | T | C | 0.598352 | 2.79E-19 | 2.63E-05 | 80.01 |
| rs6547396 | 0.00772 | 0.00107 | 3037499 | T | C | 0.579674 | 4.49E-13 | 1.71E-05 | 52.06 |
| rs2371001 | -0.00929 | 0.00105 | 3037499 | A | G | 0.500601 | 7.15E-19 | 2.58E-05 | 78.28 |
| rs561655 | -0.00891 | 0.0011 | 3037499 | A | G | 0.653403 | 5.69E-16 | 2.16E-05 | 65.61 |
| rs7788620 | 0.0167 | 0.00126 | 3037499 | A | G | 0.785541 | 8.27E-40 | 5.78E-05 | 175.67 |
| rs4846724 | 0.00767 | 0.00105 | 3037499 | A | G | 0.531722 | 2.62E-13 | 1.76E-05 | 53.36 |
| rs17266097 | 0.00879 | 0.00107 | 3037499 | T | C | 0.385679 | 2.00E-16 | 2.22E-05 | 67.49 |
| rs80171383 | 0.01462 | 0.00153 | 3037499 | A | G | 0.125085 | 1.18E-21 | 3.01E-05 | 91.31 |
| rs36048136 | 0.01179 | 0.00125 | 3037499 | A | G | 0.207607 | 4.70E-21 | 2.93E-05 | 88.96 |
| rs2364972 | 0.00719 | 0.00105 | 3037499 | A | G | 0.548476 | 7.72E-12 | 1.54E-05 | 46.89 |
| rs1387148 | -0.00946 | 0.00131 | 3037499 | A | C | 0.192809 | 5.94E-13 | 1.72E-05 | 52.15 |
| rs2964252 | 0.01024 | 0.00113 | 3037499 | A | G | 0.321605 | 1.30E-19 | 2.70E-05 | 82.12 |
| rs7150195 | 0.01365 | 0.00108 | 3037499 | T | G | 0.365306 | 1.44E-36 | 5.26E-05 | 159.74 |
| rs10481106 | -0.01237 | 0.00144 | 3037499 | A | G | 0.156283 | 8.90E-18 | 2.43E-05 | 73.79 |
| rs3013170 | 0.00707 | 0.00107 | 3037499 | T | C | 0.596427 | 4.53E-11 | 1.44E-05 | 43.66 |
| rs10887465 | 0.01276 | 0.00118 | 3037499 | A | C | 0.274546 | 4.91E-27 | 3.85E-05 | 116.93 |
| rs192230289 | -0.02665 | 0.00406 | 3037499 | T | C | 0.016307 | 5.24E-11 | 1.42E-05 | 43.09 |
| rs72748123 | -0.0118 | 0.00142 | 3037499 | A | G | 0.15967 | 7.74E-17 | 2.27E-05 | 69.05 |
| rs12893970 | -0.01272 | 0.00176 | 3037499 | A | G | 0.087219 | 4.76E-13 | 1.72E-05 | 52.23 |
| rs4879832 | -0.00637 | 0.00108 | 3037499 | T | C | 0.391423 | 3.56E-09 | 1.15E-05 | 34.79 |
| rs13138842 | 0.00834 | 0.00132 | 3037499 | A | G | 0.193224 | 2.70E-10 | 1.31E-05 | 39.92 |
| rs2295230 | 0.01399 | 0.00115 | 3037499 | A | C | 0.687111 | 5.63E-34 | 4.87E-05 | 147.99 |
| rs2154209 | -0.00779 | 0.00131 | 3037499 | T | C | 0.800832 | 2.51E-09 | 1.16E-05 | 35.36 |
| rs71366589 | 0.02255 | 0.00373 | 3037499 | T | C | 0.983862 | 1.46E-09 | 1.20E-05 | 36.55 |
| rs623200 | 0.00704 | 0.00108 | 3037499 | T | C | 0.398322 | 5.79E-11 | 1.40E-05 | 42.49 |
| rs6839051 | -0.0069 | 0.00108 | 3037499 | A | C | 0.383262 | 1.44E-10 | 1.34E-05 | 40.82 |
| rs55640000 | -0.00774 | 0.00109 | 3037499 | T | G | 0.633985 | 1.38E-12 | 1.66E-05 | 50.42 |
| rs4812818 | -0.00739 | 0.00132 | 3037499 | T | G | 0.184108 | 2.23E-08 | 1.03E-05 | 31.34 |
| rs13212041 | 0.01123 | 0.00131 | 3037499 | T | C | 0.799707 | 9.06E-18 | 2.42E-05 | 73.49 |
| rs56409354 | -0.01599 | 0.00127 | 3037499 | A | G | 0.218232 | 1.64E-36 | 5.22E-05 | 158.52 |
| rs7009856 | 0.00671 | 0.00107 | 3037499 | C | G | 0.403711 | 4.12E-10 | 1.29E-05 | 39.33 |
| rs145481578 | 0.02354 | 0.00386 | 3037499 | T | C | 0.019341 | 1.09E-09 | 1.22E-05 | 37.19 |
| rs2498018 | -0.00893 | 0.00133 | 3037499 | C | G | 0.186464 | 1.92E-11 | 1.48E-05 | 45.08 |
| rs13159124 | -0.01127 | 0.00112 | 3037499 | A | G | 0.692224 | 1.01E-23 | 3.33E-05 | 101.25 |
| rs5021426 | -0.01116 | 0.00117 | 3037499 | T | C | 0.716215 | 1.66E-21 | 3.00E-05 | 90.98 |
| rs7032484 | 0.01301 | 0.00106 | 3037499 | A | G | 0.56854 | 6.76E-35 | 4.96E-05 | 150.64 |
| rs748832 | 0.01007 | 0.00108 | 3037499 | A | G | 0.637635 | 1.61E-20 | 2.86E-05 | 86.94 |
| rs42210 | -0.01177 | 0.00116 | 3037499 | C | G | 0.691685 | 3.13E-24 | 3.39E-05 | 102.95 |
| rs6731373 | -0.00872 | 0.0011 | 3037499 | A | G | 0.320296 | 2.64E-15 | 2.07E-05 | 62.84 |
| rs10739569 | -0.00723 | 0.00114 | 3037499 | C | G | 0.696458 | 2.49E-10 | 1.32E-05 | 40.22 |
| rs28381527 | -0.01999 | 0.00195 | 3037499 | A | G | 0.076948 | 9.74E-25 | 3.46E-05 | 105.09 |
| rs137121 | 0.01266 | 0.00157 | 3037499 | T | C | 0.126809 | 8.12E-16 | 2.14E-05 | 65.02 |
| rs7338804 | -0.0084 | 0.0015 | 3037499 | A | G | 0.143363 | 2.27E-08 | 1.03E-05 | 31.36 |
| rs4876775 | -0.01126 | 0.00113 | 3037499 | A | G | 0.337943 | 1.65E-23 | 3.27E-05 | 99.29 |
| rs7983020 | -0.00893 | 0.00116 | 3037499 | T | C | 0.703157 | 1.34E-14 | 1.95E-05 | 59.26 |
| rs989996 | -0.00942 | 0.00105 | 3037499 | T | C | 0.439205 | 2.98E-19 | 2.65E-05 | 80.49 |
| rs6596534 | 0.00622 | 0.00113 | 3037499 | A | G | 0.316646 | 4.11E-08 | 9.97E-06 | 30.3 |
| rs4864881 | -0.00698 | 0.00107 | 3037499 | T | C | 0.596397 | 7.74E-11 | 1.40E-05 | 42.55 |
| rs56194430 | -0.01861 | 0.00141 | 3037499 | T | C | 0.16081 | 5.72E-40 | 5.73E-05 | 174.2 |
| rs586829 | 0.00992 | 0.00108 | 3037499 | A | G | 0.634047 | 5.49E-20 | 2.78E-05 | 84.37 |
| rs7762296 | -0.00835 | 0.00114 | 3037499 | T | C | 0.312165 | 2.02E-13 | 1.77E-05 | 53.65 |
| rs73117392 | -0.02312 | 0.00228 | 3037499 | T | C | 0.948168 | 3.56E-24 | 3.39E-05 | 102.83 |
| rs11925699 | -0.00766 | 0.00105 | 3037499 | A | G | 0.463366 | 2.73E-13 | 1.75E-05 | 53.22 |
| rs10874759 | 0.01289 | 0.0011 | 3037499 | A | G | 0.652741 | 1.08E-31 | 4.52E-05 | 137.32 |
| rs7469569 | 0.00953 | 0.0011 | 3037499 | T | C | 0.346058 | 3.57E-18 | 2.47E-05 | 75.06 |
| rs17881016 | -0.01314 | 0.0012 | 3037499 | A | G | 0.742408 | 5.27E-28 | 3.95E-05 | 119.9 |
| rs10819974 | -0.00815 | 0.00123 | 3037499 | T | C | 0.222836 | 3.95E-11 | 1.45E-05 | 43.9 |
| rs62079997 | -0.00677 | 0.00106 | 3037499 | T | C | 0.543717 | 1.50E-10 | 1.34E-05 | 40.79 |
| rs11019128 | -0.00871 | 0.00108 | 3037499 | T | C | 0.359116 | 6.35E-16 | 2.14E-05 | 65.04 |
| rs9317202 | 0.00979 | 0.00114 | 3037499 | T | C | 0.698845 | 1.27E-17 | 2.43E-05 | 73.75 |
| rs12055782 | -0.01178 | 0.00116 | 3037499 | A | G | 0.725069 | 4.15E-24 | 3.40E-05 | 103.13 |
| rs57349798 | 0.0095 | 0.00106 | 3037499 | A | G | 0.39886 | 4.32E-19 | 2.64E-05 | 80.32 |
| rs7312511 | -0.00978 | 0.00176 | 3037499 | T | C | 0.104697 | 2.55E-08 | 1.02E-05 | 30.88 |
| rs419438 | -0.0071 | 0.00105 | 3037499 | T | G | 0.539021 | 1.41E-11 | 1.51E-05 | 45.72 |
| rs6534338 | 0.01047 | 0.00114 | 3037499 | T | C | 0.297644 | 4.14E-20 | 2.78E-05 | 84.35 |
| rs139872934 | 0.0138 | 0.00182 | 3037499 | T | C | 0.09421 | 3.17E-14 | 1.89E-05 | 57.49 |
| rs795540 | -0.00798 | 0.00106 | 3037499 | T | C | 0.590622 | 5.95E-14 | 1.87E-05 | 56.68 |
| rs11724690 | 0.00949 | 0.00115 | 3037499 | T | G | 0.282861 | 1.45E-16 | 2.24E-05 | 68.1 |
| rs8044562 | -0.01193 | 0.00116 | 3037499 | A | G | 0.276794 | 8.34E-25 | 3.48E-05 | 105.77 |
| rs16962221 | 0.00748 | 0.0011 | 3037499 | A | G | 0.350955 | 1.08E-11 | 1.52E-05 | 46.24 |
| rs184654 | 0.0165 | 0.00143 | 3037499 | C | G | 0.838451 | 1.13E-30 | 4.38E-05 | 133.14 |
| rs2160514 | -0.01111 | 0.00105 | 3037499 | A | C | 0.567508 | 5.90E-26 | 3.69E-05 | 111.96 |
| rs424387 | -0.00617 | 0.00107 | 3037499 | C | G | 0.598583 | 7.09E-09 | 1.09E-05 | 33.25 |
| rs13010288 | 0.0201 | 0.00157 | 3037499 | T | G | 0.123314 | 1.30E-37 | 5.40E-05 | 163.91 |
| rs9454264 | 0.00777 | 0.00105 | 3037499 | T | C | 0.468155 | 1.42E-13 | 1.80E-05 | 54.76 |
| rs13164530 | -0.01008 | 0.00155 | 3037499 | T | G | 0.134155 | 8.20E-11 | 1.39E-05 | 42.29 |
| rs114468556 | 0.02949 | 0.00261 | 3037499 | A | T | 0.03708 | 1.22E-29 | 4.20E-05 | 127.66 |
| rs11159067 | 0.00948 | 0.00111 | 3037499 | A | G | 0.657545 | 1.84E-17 | 2.40E-05 | 72.94 |
| rs72915557 | -0.02057 | 0.00221 | 3037499 | A | G | 0.051971 | 1.16E-20 | 2.85E-05 | 86.63 |
| rs34719425 | 0.00951 | 0.00122 | 3037499 | T | C | 0.25097 | 8.07E-15 | 2.00E-05 | 60.76 |
| rs4390219 | -0.00748 | 0.00113 | 3037499 | T | C | 0.31409 | 3.37E-11 | 1.44E-05 | 43.82 |
| rs150121450 | 0.01966 | 0.00304 | 3037499 | T | G | 0.028519 | 1.02E-10 | 1.38E-05 | 41.82 |
| rs1156541 | 0.00705 | 0.00125 | 3037499 | T | C | 0.763335 | 1.51E-08 | 1.05E-05 | 31.81 |
| rs12362273 | -0.00708 | 0.00123 | 3037499 | A | C | 0.771743 | 7.89E-09 | 1.09E-05 | 33.13 |
| rs1392816 | 0.00959 | 0.00108 | 3037499 | T | C | 0.371528 | 5.42E-19 | 2.60E-05 | 78.85 |
| rs10193498 | 0.01127 | 0.0012 | 3037499 | A | T | 0.730536 | 7.89E-21 | 2.90E-05 | 88.2 |
| rs117005905 | 0.01558 | 0.00162 | 3037499 | T | C | 0.115491 | 5.63E-22 | 3.04E-05 | 92.49 |
| rs11752345 | 0.00885 | 0.00128 | 3037499 | T | C | 0.205236 | 4.33E-12 | 1.57E-05 | 47.8 |
| rs62367470 | 0.00799 | 0.00137 | 3037499 | T | C | 0.1838 | 5.83E-09 | 1.12E-05 | 34.01 |
| rs9537509 | -0.00701 | 0.0012 | 3037499 | T | C | 0.744256 | 5.32E-09 | 1.12E-05 | 34.13 |
| rs10887592 | 0.00652 | 0.00118 | 3037499 | A | G | 0.256452 | 3.34E-08 | 1.01E-05 | 30.53 |
| rs4869579 | 0.00634 | 0.00105 | 3037499 | T | C | 0.515645 | 1.44E-09 | 1.20E-05 | 36.46 |
| rs1125854 | -0.00773 | 0.00108 | 3037499 | T | C | 0.361334 | 8.96E-13 | 1.69E-05 | 51.23 |
| rs9635366 | 0.0161 | 0.00136 | 3037499 | A | G | 0.211041 | 1.98E-32 | 4.61E-05 | 140.14 |
| rs3863241 | -0.00859 | 0.00105 | 3037499 | T | C | 0.536187 | 2.86E-16 | 2.20E-05 | 66.93 |
| rs6020560 | -0.00957 | 0.00105 | 3037499 | T | C | 0.51389 | 6.94E-20 | 2.73E-05 | 83.07 |
| rs590013 | 0.01107 | 0.00111 | 3037499 | T | C | 0.650293 | 2.96E-23 | 3.27E-05 | 99.46 |
| rs3796432 | 0.00684 | 0.00108 | 3037499 | T | G | 0.365938 | 2.67E-10 | 1.32E-05 | 40.11 |
| rs2063569 | -0.00962 | 0.00108 | 3037499 | C | G | 0.373745 | 6.08E-19 | 2.61E-05 | 79.34 |
| rs903981 | -0.00715 | 0.00106 | 3037499 | T | G | 0.431414 | 1.73E-11 | 1.50E-05 | 45.5 |
| rs56306348 | 0.00748 | 0.00108 | 3037499 | A | G | 0.624761 | 3.78E-12 | 1.58E-05 | 47.97 |
| rs7172133 | 0.00742 | 0.00113 | 3037499 | A | C | 0.688605 | 4.92E-11 | 1.42E-05 | 43.12 |
| rs4467547 | 0.01322 | 0.00107 | 3037499 | T | G | 0.384416 | 3.44E-35 | 5.03E-05 | 152.65 |
| rs2795022 | 0.00581 | 0.00107 | 3037499 | T | C | 0.391176 | 4.97E-08 | 9.71E-06 | 29.48 |
| rs356903 | -0.00719 | 0.00121 | 3037499 | T | C | 0.266615 | 2.46E-09 | 1.16E-05 | 35.31 |
| rs35414043 | -0.01539 | 0.00197 | 3037499 | A | G | 0.077348 | 5.58E-15 | 2.01E-05 | 61.03 |
| rs78462816 | -0.01037 | 0.00162 | 3037499 | A | G | 0.887558 | 1.42E-10 | 1.35E-05 | 40.98 |
| rs1483148 | 0.01209 | 0.00118 | 3037499 | T | G | 0.719048 | 1.28E-24 | 3.46E-05 | 104.98 |
| rs7027019 | -0.00618 | 0.00112 | 3037499 | T | C | 0.677271 | 3.26E-08 | 1.00E-05 | 30.45 |
| rs9871964 | -0.00951 | 0.00138 | 3037499 | T | C | 0.181013 | 5.42E-12 | 1.56E-05 | 47.49 |
| rs382196 | -0.00703 | 0.00108 | 3037499 | T | G | 0.360841 | 7.53E-11 | 1.39E-05 | 42.37 |
| rs28588750 | 0.01146 | 0.00109 | 3037499 | A | G | 0.654881 | 7.58E-26 | 3.64E-05 | 110.54 |
| rs12113634 | 0.00908 | 0.00109 | 3037499 | T | C | 0.65 | 9.23E-17 | 2.28E-05 | 69.39 |
| rs77187837 | 0.01293 | 0.0016 | 3037499 | T | C | 0.122713 | 5.83E-16 | 2.15E-05 | 65.31 |
| rs1128687 | -0.00813 | 0.00106 | 3037499 | T | C | 0.59247 | 1.75E-14 | 1.94E-05 | 58.83 |
| rs4883624 | -0.00689 | 0.00105 | 3037499 | T | C | 0.465953 | 5.98E-11 | 1.42E-05 | 43.06 |
| rs1866710 | -0.01449 | 0.00116 | 3037499 | A | G | 0.285987 | 1.07E-35 | 5.14E-05 | 156.03 |
| rs6898748 | -0.00661 | 0.00105 | 3037499 | T | C | 0.56024 | 3.58E-10 | 1.30E-05 | 39.63 |
| rs1291865 | -0.01031 | 0.00105 | 3037499 | T | G | 0.53785 | 6.83E-23 | 3.17E-05 | 96.41 |
| rs6428152 | -0.00805 | 0.00117 | 3037499 | A | G | 0.290037 | 5.49E-12 | 1.56E-05 | 47.34 |
| rs7668960 | 0.01587 | 0.00145 | 3037499 | T | C | 0.17028 | 5.42E-28 | 3.94E-05 | 119.79 |
| rs1006749 | 0.0063 | 0.00105 | 3037499 | A | G | 0.536064 | 2.04E-09 | 1.19E-05 | 36 |
| rs72695265 | -0.00962 | 0.00124 | 3037499 | T | C | 0.225778 | 1.13E-14 | 1.98E-05 | 60.19 |
| rs80049472 | -0.00963 | 0.00169 | 3037499 | A | G | 0.103942 | 1.17E-08 | 1.07E-05 | 32.47 |
| rs28834466 | 0.01325 | 0.00203 | 3037499 | A | G | 0.919526 | 6.56E-11 | 1.40E-05 | 42.6 |
| rs10732635 | 0.00697 | 0.00105 | 3037499 | T | G | 0.551293 | 3.77E-11 | 1.45E-05 | 44.06 |
| rs1278537 | -0.00674 | 0.00105 | 3037499 | T | G | 0.478318 | 1.24E-10 | 1.36E-05 | 41.2 |
| rs72625841 | 0.00865 | 0.00119 | 3037499 | T | G | 0.714937 | 3.78E-13 | 1.74E-05 | 52.84 |
| rs7254263 | -0.00993 | 0.00115 | 3037499 | T | C | 0.29498 | 7.90E-18 | 2.45E-05 | 74.56 |
| rs11772108 | -0.0082 | 0.00117 | 3037499 | A | T | 0.7172 | 2.44E-12 | 1.62E-05 | 49.12 |
| rs79925479 | 0.01354 | 0.00229 | 3037499 | T | G | 0.052079 | 3.64E-09 | 1.15E-05 | 34.96 |
| rs12897763 | 0.01968 | 0.00133 | 3037499 | A | G | 0.189159 | 1.73E-49 | 7.21E-05 | 218.95 |
| rs67033024 | 0.00934 | 0.00153 | 3037499 | A | G | 0.847598 | 9.77E-10 | 1.23E-05 | 37.27 |
| rs2631535 | 0.0123 | 0.00111 | 3037499 | A | G | 0.336141 | 2.44E-28 | 4.04E-05 | 122.79 |
| rs12150824 | 0.00718 | 0.00119 | 3037499 | A | G | 0.255205 | 1.62E-09 | 1.20E-05 | 36.4 |
| rs1329125 | -0.01243 | 0.00112 | 3037499 | T | C | 0.308208 | 1.06E-28 | 4.05E-05 | 123.17 |
| rs2630767 | 0.013 | 0.00105 | 3037499 | T | C | 0.474376 | 6.56E-35 | 5.05E-05 | 153.29 |
| rs12632133 | 0.01129 | 0.002 | 3037499 | A | G | 0.077903 | 1.57E-08 | 1.05E-05 | 31.87 |
| rs7657329 | -0.00856 | 0.00112 | 3037499 | A | C | 0.304512 | 2.59E-14 | 1.92E-05 | 58.41 |
| rs80352808 | -0.00751 | 0.00116 | 3037499 | A | G | 0.286803 | 1.15E-10 | 1.38E-05 | 41.91 |
| rs4667878 | 0.00757 | 0.00134 | 3037499 | A | T | 0.186126 | 1.66E-08 | 1.05E-05 | 31.91 |
| rs6466819 | -0.01031 | 0.00109 | 3037499 | A | G | 0.370696 | 2.49E-21 | 2.95E-05 | 89.47 |
| rs17503473 | 0.01194 | 0.00114 | 3037499 | T | C | 0.705266 | 1.13E-25 | 3.61E-05 | 109.7 |
| rs9922788 | 0.01028 | 0.00106 | 3037499 | A | G | 0.408331 | 2.12E-22 | 3.10E-05 | 94.05 |
| rs10865834 | -0.01049 | 0.00121 | 3037499 | A | G | 0.253896 | 3.58E-18 | 2.47E-05 | 75.16 |
| rs2884364 | -0.01595 | 0.00108 | 3037499 | A | G | 0.374299 | 1.29E-49 | 7.18E-05 | 218.11 |
| rs12155540 | 0.01172 | 0.00111 | 3037499 | T | C | 0.329889 | 6.83E-26 | 3.67E-05 | 111.48 |
| rs62114178 | 0.00685 | 0.00108 | 3037499 | A | G | 0.360333 | 2.49E-10 | 1.32E-05 | 40.23 |
| rs11664320 | 0.01759 | 0.00106 | 3037499 | T | C | 0.591346 | 2.21E-62 | 9.06E-05 | 275.37 |
| rs7356921 | -0.01482 | 0.00122 | 3037499 | T | C | 0.24595 | 9.09E-34 | 4.86E-05 | 147.56 |
| rs7905026 | -0.00748 | 0.00119 | 3037499 | A | C | 0.286203 | 3.62E-10 | 1.30E-05 | 39.51 |
| rs2026037 | 0.01545 | 0.00143 | 3037499 | T | C | 0.848753 | 4.56E-27 | 3.84E-05 | 116.73 |
| rs171697 | 0.01517 | 0.00111 | 3037499 | C | G | 0.687604 | 2.37E-42 | 6.15E-05 | 186.78 |
| rs35583726 | 0.01185 | 0.0014 | 3037499 | A | G | 0.836372 | 3.13E-17 | 2.36E-05 | 71.64 |
| rs1861786 | -0.00764 | 0.00108 | 3037499 | A | G | 0.376948 | 1.31E-12 | 1.65E-05 | 50.04 |
| rs11181124 | -0.00825 | 0.00128 | 3037499 | T | C | 0.797351 | 1.06E-10 | 1.37E-05 | 41.54 |
| rs10763979 | 0.0092 | 0.00108 | 3037499 | A | G | 0.61546 | 1.48E-17 | 2.39E-05 | 72.57 |
| rs1058790 | -0.01396 | 0.00136 | 3037499 | A | G | 0.806021 | 1.37E-24 | 3.47E-05 | 105.36 |
| rs1786313 | 0.00771 | 0.00113 | 3037499 | T | C | 0.308885 | 8.15E-12 | 1.53E-05 | 46.55 |
| rs2841425 | -0.00853 | 0.00137 | 3037499 | A | C | 0.180413 | 4.43E-10 | 1.28E-05 | 38.77 |
| rs7936656 | -0.00833 | 0.00112 | 3037499 | A | G | 0.313813 | 1.13E-13 | 1.82E-05 | 55.32 |
| rs9914251 | 0.0077 | 0.00122 | 3037499 | A | G | 0.750616 | 2.97E-10 | 1.31E-05 | 39.83 |
| rs6499084 | 0.00721 | 0.0011 | 3037499 | A | G | 0.353911 | 5.56E-11 | 1.41E-05 | 42.96 |
| rs6911986 | -0.00828 | 0.00118 | 3037499 | T | C | 0.268833 | 2.46E-12 | 1.62E-05 | 49.24 |
| rs9993133 | 0.00761 | 0.00138 | 3037499 | T | C | 0.810132 | 3.50E-08 | 1.00E-05 | 30.41 |
| rs77882218 | -0.02563 | 0.00315 | 3037499 | T | C | 0.969603 | 4.06E-16 | 2.18E-05 | 66.2 |
| rs1485272 | -0.00718 | 0.0011 | 3037499 | T | C | 0.644888 | 7.17E-11 | 1.40E-05 | 42.61 |
| rs17563464 | -0.01268 | 0.00127 | 3037499 | A | C | 0.202525 | 2.35E-23 | 3.28E-05 | 99.69 |
| rs60053512 | 0.01233 | 0.00144 | 3037499 | A | G | 0.167447 | 1.28E-17 | 2.41E-05 | 73.32 |
| rs10014934 | 0.00913 | 0.00108 | 3037499 | T | C | 0.611318 | 3.09E-17 | 2.35E-05 | 71.47 |
| rs4650228 | -0.00847 | 0.00105 | 3037499 | A | G | 0.462196 | 6.69E-16 | 2.14E-05 | 65.07 |
| rs7236339 | -0.01531 | 0.00127 | 3037499 | A | G | 0.203419 | 1.57E-33 | 4.78E-05 | 145.33 |
| rs2570492 | 0.01299 | 0.00107 | 3037499 | A | G | 0.395719 | 5.95E-34 | 4.85E-05 | 147.38 |
| rs7026972 | -0.0115 | 0.00109 | 3037499 | T | C | 0.649461 | 7.08E-26 | 3.66E-05 | 111.31 |
| rs80257979 | -0.02623 | 0.00295 | 3037499 | T | G | 0.970157 | 5.77E-19 | 2.60E-05 | 79.06 |
| rs9937449 | 0.00777 | 0.00105 | 3037499 | T | C | 0.550693 | 1.58E-13 | 1.80E-05 | 54.76 |
| rs34984805 | 0.0074 | 0.00105 | 3037499 | T | C | 0.530382 | 1.54E-12 | 1.64E-05 | 49.67 |
| rs72890842 | 0.0126 | 0.00122 | 3037499 | T | G | 0.765152 | 4.76E-25 | 3.51E-05 | 106.66 |
| rs9985296 | -0.01031 | 0.00108 | 3037499 | T | C | 0.384232 | 9.86E-22 | 3.00E-05 | 91.13 |
| rs9555651 | 0.01106 | 0.0014 | 3037499 | T | C | 0.155266 | 3.03E-15 | 2.05E-05 | 62.41 |
| rs61867294 | -0.01651 | 0.00131 | 3037499 | A | G | 0.78688 | 2.05E-36 | 5.23E-05 | 158.84 |
| rs7131691 | -0.00748 | 0.00105 | 3037499 | A | G | 0.484247 | 1.09E-12 | 1.67E-05 | 50.75 |
| rs4663617 | 0.01035 | 0.00123 | 3037499 | A | T | 0.230413 | 3.17E-17 | 2.33E-05 | 70.81 |
| rs1632822 | 0.00588 | 0.00108 | 3037499 | C | G | 0.369125 | 4.91E-08 | 9.76E-06 | 29.64 |
| rs10180845 | -0.0096 | 0.00109 | 3037499 | T | C | 0.656098 | 1.02E-18 | 2.55E-05 | 77.57 |
| rs2504846 | -0.02579 | 0.00328 | 3037499 | T | C | 0.031675 | 3.74E-15 | 2.04E-05 | 61.82 |
| rs73625120 | 0.01532 | 0.00271 | 3037499 | A | G | 0.04033 | 1.51E-08 | 1.05E-05 | 31.96 |
| rs474000 | 0.00642 | 0.00105 | 3037499 | A | G | 0.430474 | 1.10E-09 | 1.23E-05 | 37.38 |
| rs9859719 | -0.01128 | 0.00126 | 3037499 | A | G | 0.211395 | 3.67E-19 | 2.64E-05 | 80.15 |
| rs116173579 | -0.01649 | 0.00293 | 3037499 | T | C | 0.034755 | 1.74E-08 | 1.04E-05 | 31.67 |
| rs2430813 | 0.01103 | 0.00125 | 3037499 | T | C | 0.764875 | 8.62E-19 | 2.56E-05 | 77.86 |
| rs9930253 | -0.00986 | 0.00117 | 3037499 | A | T | 0.280551 | 3.12E-17 | 2.34E-05 | 71.02 |
| rs62172117 | 0.01356 | 0.0011 | 3037499 | A | G | 0.343286 | 3.35E-35 | 5.00E-05 | 151.96 |
| rs422247 | 0.00753 | 0.0012 | 3037499 | T | C | 0.739452 | 3.56E-10 | 1.30E-05 | 39.38 |
| rs7328289 | -0.00703 | 0.00127 | 3037499 | T | C | 0.794041 | 3.29E-08 | 1.01E-05 | 30.64 |
| rs565960 | 0.00875 | 0.00116 | 3037499 | T | C | 0.702325 | 3.75E-14 | 1.87E-05 | 56.9 |
| rs113389222 | -0.0117 | 0.0016 | 3037499 | T | C | 0.11409 | 2.68E-13 | 1.76E-05 | 53.47 |
| rs1738050 | -0.011 | 0.00108 | 3037499 | C | G | 0.630459 | 1.49E-24 | 3.42E-05 | 103.74 |
| rs158839 | -0.00623 | 0.00107 | 3037499 | A | C | 0.617278 | 6.42E-09 | 1.12E-05 | 33.9 |
| rs6580068 | -0.00931 | 0.00127 | 3037499 | T | C | 0.224638 | 1.96E-13 | 1.77E-05 | 53.74 |
| rs6906818 | 0.00823 | 0.00107 | 3037499 | T | C | 0.599384 | 1.26E-14 | 1.95E-05 | 59.16 |
| rs7449561 | 0.00973 | 0.00125 | 3037499 | A | G | 0.234278 | 6.83E-15 | 1.99E-05 | 60.59 |
| rs7304399 | 0.01093 | 0.00105 | 3037499 | A | G | 0.4585 | 2.16E-25 | 3.57E-05 | 108.36 |
| rs9540731 | 0.01075 | 0.00105 | 3037499 | T | C | 0.500693 | 9.93E-25 | 3.45E-05 | 104.82 |
| rs1490834 | -0.00639 | 0.00105 | 3037499 | A | G | 0.549892 | 1.15E-09 | 1.22E-05 | 37.04 |
| rs792213 | 0.00733 | 0.00106 | 3037499 | A | C | 0.41178 | 5.31E-12 | 1.57E-05 | 47.82 |
| rs300145 | -0.00726 | 0.00111 | 3037499 | T | C | 0.663736 | 5.87E-11 | 1.41E-05 | 42.78 |
| rs61964892 | -0.00785 | 0.00128 | 3037499 | T | C | 0.781999 | 8.76E-10 | 1.24E-05 | 37.61 |
| rs6801153 | -0.00856 | 0.00122 | 3037499 | T | C | 0.247351 | 2.11E-12 | 1.62E-05 | 49.23 |
| rs141503359 | -0.02835 | 0.00478 | 3037499 | T | C | 0.014259 | 2.97E-09 | 1.16E-05 | 35.18 |
| rs2174752 | -0.00862 | 0.00105 | 3037499 | T | G | 0.435186 | 2.36E-16 | 2.22E-05 | 67.4 |
| rs73055556 | 0.01112 | 0.00152 | 3037499 | A | G | 0.135587 | 2.51E-13 | 1.76E-05 | 53.52 |
| rs3869097 | -0.009 | 0.00112 | 3037499 | T | C | 0.333061 | 1.10E-15 | 2.13E-05 | 64.57 |
| rs4805761 | -0.01395 | 0.00145 | 3037499 | A | G | 0.150277 | 5.97E-22 | 3.05E-05 | 92.56 |
| rs6545559 | -0.00767 | 0.00112 | 3037499 | T | C | 0.316554 | 8.22E-12 | 1.54E-05 | 46.9 |
| rs2210174 | 0.00613 | 0.00108 | 3037499 | T | C | 0.622498 | 1.33E-08 | 1.06E-05 | 32.22 |
| rs4679353 | 0.00717 | 0.00108 | 3037499 | A | G | 0.614844 | 3.00E-11 | 1.45E-05 | 44.07 |
| rs6502670 | -0.00746 | 0.00109 | 3037499 | T | G | 0.617416 | 7.56E-12 | 1.54E-05 | 46.84 |
| rs72778512 | -0.01223 | 0.00185 | 3037499 | C | G | 0.913335 | 3.81E-11 | 1.44E-05 | 43.7 |
| rs4409028 | 0.00596 | 0.00109 | 3037499 | A | C | 0.63611 | 4.87E-08 | 9.84E-06 | 29.9 |
| rs12506222 | -0.01423 | 0.00105 | 3037499 | T | C | 0.467354 | 1.50E-41 | 6.05E-05 | 183.67 |
| rs1835388 | -0.00815 | 0.00105 | 3037499 | T | C | 0.482738 | 7.69E-15 | 1.98E-05 | 60.25 |
| rs5765717 | -0.00724 | 0.00105 | 3037499 | T | G | 0.478164 | 4.66E-12 | 1.57E-05 | 47.54 |
| rs116750901 | 0.0131 | 0.00237 | 3037499 | A | G | 0.038266 | 3.10E-08 | 1.01E-05 | 30.55 |
| rs1483814 | 0.00734 | 0.00131 | 3037499 | T | G | 0.191407 | 2.01E-08 | 1.03E-05 | 31.39 |
| rs6093705 | 0.00954 | 0.00112 | 3037499 | A | C | 0.682984 | 1.89E-17 | 2.39E-05 | 72.55 |
| rs3110127 | -0.00625 | 0.00111 | 3037499 | T | C | 0.349877 | 1.92E-08 | 1.04E-05 | 31.7 |
| rs837080 | -0.00977 | 0.00105 | 3037499 | T | C | 0.535048 | 1.07E-20 | 2.85E-05 | 86.58 |
| rs10202640 | -0.01027 | 0.00132 | 3037499 | A | G | 0.195966 | 8.23E-15 | 1.99E-05 | 60.53 |
| rs10867548 | 0.0093 | 0.00149 | 3037499 | A | G | 0.861488 | 4.33E-10 | 1.28E-05 | 38.96 |
| rs7240432 | 0.01039 | 0.00109 | 3037499 | T | G | 0.360687 | 1.74E-21 | 2.99E-05 | 90.86 |
| rs72717150 | -0.01942 | 0.00199 | 3037499 | A | G | 0.069464 | 1.68E-22 | 3.14E-05 | 95.23 |
| rs1880692 | 0.00767 | 0.00105 | 3037499 | A | G | 0.541839 | 2.78E-13 | 1.76E-05 | 53.36 |
| rs3783006 | 0.01309 | 0.00105 | 3037499 | C | G | 0.418817 | 1.69E-35 | 5.12E-05 | 155.42 |
| rs181164682 | 0.02436 | 0.00375 | 3037499 | A | T | 0.014999 | 8.16E-11 | 1.39E-05 | 42.2 |
| rs2239736 | 0.00856 | 0.00105 | 3037499 | T | C | 0.510286 | 3.04E-16 | 2.19E-05 | 66.46 |
| rs17088142 | -0.00683 | 0.00105 | 3037499 | T | G | 0.452787 | 7.51E-11 | 1.39E-05 | 42.31 |
| rs17649841 | -0.008 | 0.00123 | 3037499 | A | C | 0.227348 | 8.45E-11 | 1.39E-05 | 42.3 |
| rs74677779 | -0.01561 | 0.00257 | 3037499 | C | G | 0.956083 | 1.23E-09 | 1.21E-05 | 36.89 |
| rs9652254 | 0.00649 | 0.00106 | 3037499 | A | G | 0.602541 | 1.03E-09 | 1.23E-05 | 37.49 |
| rs6704768 | -0.01533 | 0.00106 | 3037499 | A | G | 0.541839 | 9.67E-48 | 6.89E-05 | 209.16 |
| rs11028323 | -0.00984 | 0.00105 | 3037499 | A | C | 0.514675 | 6.20E-21 | 2.89E-05 | 87.82 |
| rs17224289 | -0.01317 | 0.00161 | 3037499 | T | C | 0.117632 | 3.10E-16 | 2.20E-05 | 66.91 |
| rs6960056 | 0.01286 | 0.00105 | 3037499 | A | G | 0.450616 | 3.50E-34 | 4.94E-05 | 150 |
| rs7979979 | -0.01165 | 0.00122 | 3037499 | T | C | 0.226548 | 1.20E-21 | 3.00E-05 | 91.19 |
| rs4726070 | 0.01021 | 0.00107 | 3037499 | A | G | 0.593086 | 1.05E-21 | 3.00E-05 | 91.05 |
| rs10186870 | -0.00602 | 0.00107 | 3037499 | T | G | 0.605759 | 1.91E-08 | 1.04E-05 | 31.65 |
| rs17565975 | -0.01211 | 0.00105 | 3037499 | A | G | 0.529504 | 1.25E-30 | 4.38E-05 | 133.02 |
| rs2214631 | 0.00706 | 0.00117 | 3037499 | A | T | 0.311287 | 1.43E-09 | 1.20E-05 | 36.41 |
| rs11773992 | -0.0138 | 0.00137 | 3037499 | C | G | 0.16178 | 5.84E-24 | 3.34E-05 | 101.47 |
| rs4715244 | 0.00716 | 0.00126 | 3037499 | T | G | 0.212119 | 1.28E-08 | 1.06E-05 | 32.29 |
| rs7677621 | -0.01029 | 0.00109 | 3037499 | T | C | 0.355328 | 5.54E-21 | 2.93E-05 | 89.12 |
| rs2021871 | 0.00654 | 0.00112 | 3037499 | T | C | 0.66854 | 4.87E-09 | 1.12E-05 | 34.1 |
| rs17096452 | 0.01138 | 0.00127 | 3037499 | A | T | 0.791284 | 3.18E-19 | 2.64E-05 | 80.29 |
| rs72779695 | -0.01397 | 0.00162 | 3037499 | T | C | 0.11466 | 7.03E-18 | 2.45E-05 | 74.36 |
| rs34316 | 0.01629 | 0.00106 | 3037499 | A | C | 0.434694 | 6.58E-53 | 7.77E-05 | 236.17 |
| rs76076331 | 0.02206 | 0.00156 | 3037499 | T | C | 0.122605 | 1.54E-45 | 6.58E-05 | 199.97 |
| rs10256396 | 0.00654 | 0.00111 | 3037499 | A | C | 0.35539 | 3.34E-09 | 1.14E-05 | 34.71 |
| rs12151015 | -0.00956 | 0.00164 | 3037499 | C | G | 0.888343 | 5.60E-09 | 1.12E-05 | 33.98 |
| rs2916490 | -0.01042 | 0.00113 | 3037499 | A | G | 0.304697 | 4.04E-20 | 2.80E-05 | 85.03 |
| rs62086577 | 0.00739 | 0.00128 | 3037499 | A | G | 0.210625 | 8.10E-09 | 1.10E-05 | 33.33 |
| rs10895719 | -0.01068 | 0.00128 | 3037499 | T | C | 0.777826 | 6.65E-17 | 2.29E-05 | 69.62 |
| rs77899607 | 0.01308 | 0.00157 | 3037499 | T | C | 0.108131 | 6.50E-17 | 2.29E-05 | 69.41 |
| rs1884368 | 0.00909 | 0.00122 | 3037499 | T | C | 0.232338 | 7.73E-14 | 1.83E-05 | 55.51 |
| rs13244634 | -0.00784 | 0.00123 | 3037499 | T | C | 0.238512 | 2.10E-10 | 1.34E-05 | 40.63 |
| rs2304282 | 0.011 | 0.00106 | 3037499 | A | G | 0.407407 | 4.04E-25 | 3.55E-05 | 107.69 |
| rs7039819 | 0.00919 | 0.00106 | 3037499 | A | G | 0.58146 | 4.39E-18 | 2.47E-05 | 75.17 |
| rs10496632 | 0.01114 | 0.00116 | 3037499 | C | G | 0.288112 | 8.30E-22 | 3.04E-05 | 92.23 |
| rs11138947 | 0.00958 | 0.00117 | 3037499 | T | C | 0.711718 | 2.22E-16 | 2.21E-05 | 67.04 |
| rs4989480 | -0.00887 | 0.00151 | 3037499 | A | G | 0.856683 | 4.02E-09 | 1.14E-05 | 34.51 |
| rs4911257 | 0.00979 | 0.00107 | 3037499 | T | C | 0.606729 | 7.96E-20 | 2.76E-05 | 83.71 |
| rs12962980 | 0.01682 | 0.00136 | 3037499 | C | G | 0.187743 | 2.40E-35 | 5.04E-05 | 152.96 |
| rs10765789 | 0.01078 | 0.00108 | 3037499 | A | G | 0.369233 | 1.35E-23 | 3.28E-05 | 99.63 |
| rs2352430 | -0.00783 | 0.00124 | 3037499 | T | C | 0.77048 | 3.11E-10 | 1.31E-05 | 39.87 |
| rs61160187 | -0.01963 | 0.00107 | 3037499 | A | G | 0.608362 | 1.32E-74 | 1.11E-04 | 336.57 |
| rs1407350 | 0.00784 | 0.0011 | 3037499 | T | G | 0.646705 | 9.13E-13 | 1.67E-05 | 50.8 |
| rs6930903 | 0.01587 | 0.00133 | 3037499 | A | G | 0.738374 | 4.73E-33 | 4.69E-05 | 142.38 |
| rs2867932 | 0.00715 | 0.00107 | 3037499 | A | G | 0.38956 | 2.65E-11 | 1.47E-05 | 44.65 |
| rs35414759 | -0.01094 | 0.00135 | 3037499 | T | C | 0.830274 | 4.69E-16 | 2.16E-05 | 65.67 |
| rs117494884 | -0.01456 | 0.00225 | 3037499 | T | C | 0.054512 | 1.04E-10 | 1.38E-05 | 41.88 |
| rs17669337 | -0.01008 | 0.00106 | 3037499 | T | C | 0.405174 | 2.46E-21 | 2.98E-05 | 90.43 |
| rs1452366 | -0.01191 | 0.0011 | 3037499 | T | G | 0.352618 | 1.58E-27 | 3.86E-05 | 117.23 |
| rs72672601 | -0.00953 | 0.00106 | 3037499 | A | G | 0.578873 | 2.02E-19 | 2.66E-05 | 80.83 |
| rs1378893 | 0.00908 | 0.00123 | 3037499 | A | G | 0.22627 | 1.52E-13 | 1.79E-05 | 54.5 |
| rs60307735 | 0.00923 | 0.00111 | 3037499 | T | C | 0.329489 | 6.83E-17 | 2.28E-05 | 69.14 |
| rs4850810 | -0.01315 | 0.00105 | 3037499 | T | G | 0.519957 | 6.68E-36 | 5.16E-05 | 156.85 |
| rs34305371 | 0.03143 | 0.00176 | 3037499 | A | G | 0.085787 | 1.77E-71 | 1.05E-04 | 318.91 |
| rs12126231 | 0.01174 | 0.00108 | 3037499 | A | G | 0.615984 | 9.70E-28 | 3.89E-05 | 118.16 |
| rs35951883 | -0.00805 | 0.00127 | 3037499 | A | G | 0.790345 | 2.33E-10 | 1.32E-05 | 40.18 |
| rs28438078 | -0.00964 | 0.00143 | 3037499 | A | G | 0.142393 | 1.72E-11 | 1.50E-05 | 45.44 |
| rs55975662 | 0.01532 | 0.00149 | 3037499 | A | G | 0.84612 | 1.09E-24 | 3.48E-05 | 105.72 |
| rs1871745 | 0.01019 | 0.00129 | 3037499 | A | G | 0.765938 | 2.19E-15 | 2.05E-05 | 62.4 |
| rs1516036 | 0.00707 | 0.00105 | 3037499 | A | G | 0.546905 | 1.57E-11 | 1.49E-05 | 45.34 |
| rs2216053 | -0.00727 | 0.00119 | 3037499 | T | G | 0.736742 | 1.06E-09 | 1.23E-05 | 37.32 |
| rs77128898 | -0.0242 | 0.00295 | 3037499 | T | C | 0.030952 | 2.09E-16 | 2.22E-05 | 67.3 |
| rs13122283 | 0.00695 | 0.00106 | 3037499 | A | G | 0.403203 | 5.08E-11 | 1.42E-05 | 42.99 |
| rs4127499 | 0.01096 | 0.0011 | 3037499 | A | G | 0.324962 | 2.90E-23 | 3.27E-05 | 99.27 |
| rs12431579 | -0.00609 | 0.00109 | 3037499 | A | G | 0.639837 | 2.25E-08 | 1.03E-05 | 31.22 |
| rs58694847 | -0.01174 | 0.00119 | 3037499 | C | G | 0.275593 | 7.84E-23 | 3.20E-05 | 97.33 |
| rs73874322 | -0.01431 | 0.00217 | 3037499 | C | G | 0.056637 | 4.21E-11 | 1.43E-05 | 43.49 |
| rs6782698 | 0.01383 | 0.00121 | 3037499 | A | G | 0.758007 | 4.81E-30 | 4.30E-05 | 130.64 |
| rs6715321 | -0.00979 | 0.00106 | 3037499 | T | C | 0.439205 | 2.20E-20 | 2.81E-05 | 85.3 |
| rs56355837 | -0.00842 | 0.00111 | 3037499 | A | C | 0.685864 | 2.94E-14 | 1.89E-05 | 57.54 |
| rs72993796 | 0.01485 | 0.00163 | 3037499 | T | C | 0.891946 | 8.58E-20 | 2.73E-05 | 83 |
| rs10063055 | -0.01249 | 0.00123 | 3037499 | T | C | 0.229689 | 3.26E-24 | 3.39E-05 | 103.11 |
| rs62199988 | -0.00862 | 0.00107 | 3037499 | C | G | 0.622174 | 7.87E-16 | 2.14E-05 | 64.9 |
| rs669952 | 0.0078 | 0.00134 | 3037499 | A | G | 0.181583 | 6.31E-09 | 1.12E-05 | 33.88 |
| rs708912 | 0.01037 | 0.00129 | 3037499 | T | C | 0.780074 | 9.00E-16 | 2.13E-05 | 64.62 |
| rs77926719 | 0.01097 | 0.0016 | 3037499 | A | G | 0.106606 | 7.25E-12 | 1.55E-05 | 47.01 |
| rs72725852 | 0.00663 | 0.00112 | 3037499 | T | C | 0.316184 | 3.28E-09 | 1.15E-05 | 35.04 |
| rs72829857 | -0.01705 | 0.00121 | 3037499 | A | G | 0.759871 | 7.31E-45 | 6.54E-05 | 198.55 |
| rs10853981 | -0.00868 | 0.00111 | 3037499 | A | G | 0.325146 | 6.64E-15 | 2.01E-05 | 61.15 |
| rs1462852 | -0.00729 | 0.00111 | 3037499 | A | T | 0.646951 | 5.27E-11 | 1.42E-05 | 43.13 |
| rs10740572 | -0.00844 | 0.00126 | 3037499 | A | T | 0.777795 | 1.92E-11 | 1.48E-05 | 44.87 |
| rs11643516 | -0.00812 | 0.00106 | 3037499 | T | C | 0.43166 | 2.22E-14 | 1.93E-05 | 58.68 |
| rs7933521 | 0.00826 | 0.00149 | 3037499 | T | C | 0.839452 | 2.85E-08 | 1.01E-05 | 30.73 |
| rs7520927 | -0.00684 | 0.00105 | 3037499 | A | G | 0.478226 | 6.38E-11 | 1.40E-05 | 42.44 |
| rs11157931 | -0.01152 | 0.00107 | 3037499 | A | C | 0.396628 | 5.98E-27 | 3.82E-05 | 115.91 |
| rs117520198 | 0.0107 | 0.00133 | 3037499 | A | G | 0.1747 | 1.01E-15 | 2.13E-05 | 64.72 |
| rs6028084 | 0.01514 | 0.00107 | 3037499 | T | C | 0.610764 | 3.44E-45 | 6.59E-05 | 200.21 |
| rs7515450 | 0.00802 | 0.00107 | 3037499 | A | T | 0.410718 | 7.28E-14 | 1.85E-05 | 56.18 |
| rs116068530 | 0.01945 | 0.00353 | 3037499 | A | G | 0.019603 | 3.71E-08 | 9.99E-06 | 30.36 |
| rs213014 | -0.00673 | 0.00105 | 3037499 | T | C | 0.548352 | 1.49E-10 | 1.35E-05 | 41.08 |
| rs13161488 | -0.00869 | 0.0011 | 3037499 | T | C | 0.659578 | 2.45E-15 | 2.05E-05 | 62.41 |
| rs3731507 | 0.02758 | 0.00259 | 3037499 | T | C | 0.05211 | 1.71E-26 | 3.73E-05 | 113.39 |
| rs13030436 | 0.00787 | 0.00106 | 3037499 | T | C | 0.408777 | 8.77E-14 | 1.81E-05 | 55.12 |
| rs76799289 | -0.02828 | 0.00449 | 3037499 | T | G | 0.009763 | 2.96E-10 | 1.31E-05 | 39.67 |
| rs35226705 | 0.00614 | 0.00105 | 3037499 | A | C | 0.543686 | 4.86E-09 | 1.13E-05 | 34.19 |
| rs2238483 | 0.01009 | 0.0014 | 3037499 | A | G | 0.814721 | 5.31E-13 | 1.71E-05 | 51.94 |
| rs62156740 | -0.02875 | 0.00387 | 3037499 | A | T | 0.985063 | 1.17E-13 | 1.82E-05 | 55.19 |
| rs912609 | 0.01235 | 0.00125 | 3037499 | T | G | 0.240437 | 6.57E-23 | 3.21E-05 | 97.61 |
| rs9359939 | -0.01085 | 0.00122 | 3037499 | A | C | 0.237558 | 6.65E-19 | 2.60E-05 | 79.09 |
| rs3747631 | 0.02043 | 0.00127 | 3037499 | C | G | 0.201217 | 7.17E-58 | 8.52E-05 | 258.78 |
| rs9411336 | -0.01285 | 0.00112 | 3037499 | T | C | 0.337296 | 1.56E-30 | 4.33E-05 | 131.63 |
| rs4882141 | 0.00662 | 0.0011 | 3037499 | T | C | 0.649091 | 1.53E-09 | 1.19E-05 | 36.22 |
| rs975303 | -0.01477 | 0.00138 | 3037499 | A | G | 0.839082 | 1.17E-26 | 3.77E-05 | 114.55 |
| rs117520996 | 0.02196 | 0.0025 | 3037499 | T | G | 0.041638 | 1.47E-18 | 2.54E-05 | 77.16 |
| rs1888765 | 0.00901 | 0.00126 | 3037499 | C | G | 0.767 | 7.09E-13 | 1.68E-05 | 51.13 |
| rs4447403 | 0.00695 | 0.0012 | 3037499 | T | C | 0.742947 | 6.98E-09 | 1.10E-05 | 33.54 |
| rs76878669 | 0.01212 | 0.00123 | 3037499 | C | G | 0.766677 | 6.48E-23 | 3.20E-05 | 97.09 |
| rs17248751 | -0.01375 | 0.00127 | 3037499 | A | G | 0.798676 | 3.42E-27 | 3.86E-05 | 117.22 |
| rs2764684 | 0.01501 | 0.00139 | 3037499 | T | C | 0.847028 | 2.64E-27 | 3.84E-05 | 116.61 |
| rs28458909 | -0.01902 | 0.00164 | 3037499 | T | C | 0.126255 | 5.98E-31 | 4.43E-05 | 134.5 |
| rs6088618 | 0.00813 | 0.00105 | 3037499 | A | G | 0.438405 | 1.08E-14 | 1.97E-05 | 59.95 |
| rs10283803 | -0.00575 | 0.00105 | 3037499 | T | C | 0.531306 | 3.91E-08 | 9.87E-06 | 29.99 |
| rs7151326 | -0.0087 | 0.00107 | 3037499 | T | G | 0.412242 | 5.69E-16 | 2.18E-05 | 66.11 |
| rs8110747 | -0.00659 | 0.00106 | 3037499 | T | C | 0.389051 | 6.03E-10 | 1.27E-05 | 38.65 |
| rs56378598 | 0.00826 | 0.00119 | 3037499 | T | C | 0.290607 | 4.27E-12 | 1.59E-05 | 48.18 |
| rs13278836 | 0.01373 | 0.00164 | 3037499 | A | G | 0.110748 | 5.10E-17 | 2.31E-05 | 70.09 |
| rs9899056 | 0.00698 | 0.00107 | 3037499 | A | G | 0.397151 | 6.71E-11 | 1.40E-05 | 42.55 |
| rs273028 | -0.01353 | 0.00199 | 3037499 | A | T | 0.085756 | 9.32E-12 | 1.52E-05 | 46.23 |
| rs7650602 | -0.00998 | 0.00106 | 3037499 | T | C | 0.567601 | 3.19E-21 | 2.92E-05 | 88.64 |
| rs78280128 | -0.01989 | 0.00187 | 3037499 | A | G | 0.920234 | 1.98E-26 | 3.72E-05 | 113.13 |
| rs12981405 | -0.01106 | 0.00141 | 3037499 | T | C | 0.158577 | 4.09E-15 | 2.03E-05 | 61.53 |
| rs55897719 | -0.01267 | 0.00115 | 3037499 | A | C | 0.286973 | 2.04E-28 | 4.00E-05 | 121.38 |
| rs3108680 | 0.0102 | 0.0011 | 3037499 | T | C | 0.668032 | 1.89E-20 | 2.83E-05 | 85.98 |
| rs717997 | 0.01297 | 0.00106 | 3037499 | A | G | 0.421312 | 2.49E-34 | 4.93E-05 | 149.72 |
| rs10189857 | 0.01378 | 0.00106 | 3037499 | A | G | 0.562889 | 7.74E-39 | 5.56E-05 | 169 |
| rs35733856 | -0.01188 | 0.00107 | 3037499 | A | G | 0.559732 | 8.26E-29 | 4.06E-05 | 123.27 |
| rs62543169 | -0.01165 | 0.00106 | 3037499 | T | C | 0.404327 | 3.33E-28 | 3.98E-05 | 120.79 |
| rs11245450 | 0.00686 | 0.00106 | 3037499 | A | G | 0.420157 | 1.06E-10 | 1.38E-05 | 41.88 |
| rs7626224 | 0.00784 | 0.00122 | 3037499 | T | G | 0.76215 | 1.36E-10 | 1.36E-05 | 41.3 |
| rs6956241 | 0.01341 | 0.00151 | 3037499 | T | G | 0.144995 | 5.88E-19 | 2.60E-05 | 78.87 |
| rs192436652 | -0.02765 | 0.00333 | 3037499 | T | C | 0.027302 | 9.90E-17 | 2.27E-05 | 68.94 |
| rs12435895 | -0.00798 | 0.00125 | 3037499 | A | G | 0.773961 | 1.55E-10 | 1.34E-05 | 40.76 |
| rs853199 | 0.00859 | 0.00109 | 3037499 | T | C | 0.332476 | 3.84E-15 | 2.04E-05 | 62.11 |
| rs4358081 | -0.0097 | 0.00105 | 3037499 | A | C | 0.533554 | 2.14E-20 | 2.81E-05 | 85.34 |
| rs12273435 | -0.01409 | 0.00131 | 3037499 | A | G | 0.187866 | 6.11E-27 | 3.81E-05 | 115.69 |
| rs11218422 | 0.00879 | 0.00106 | 3037499 | T | C | 0.565907 | 1.10E-16 | 2.26E-05 | 68.76 |
| rs17126938 | -0.01264 | 0.00151 | 3037499 | T | C | 0.858285 | 5.79E-17 | 2.31E-05 | 70.07 |
| rs4557790 | 0.02049 | 0.00105 | 3037499 | A | C | 0.567154 | 4.88E-84 | 1.25E-04 | 380.81 |
| rs75624576 | 0.01536 | 0.00175 | 3037499 | T | G | 0.096274 | 1.36E-18 | 2.54E-05 | 77.04 |
| rs61747226 | -0.0189 | 0.0026 | 3037499 | T | C | 0.040946 | 3.41E-13 | 1.74E-05 | 52.84 |
| rs56059718 | -0.01216 | 0.00132 | 3037499 | A | C | 0.196951 | 3.81E-20 | 2.79E-05 | 84.86 |
| rs11774212 | 0.01567 | 0.00105 | 3037499 | T | C | 0.496458 | 1.70E-50 | 7.33E-05 | 222.72 |
| rs10401329 | 0.01094 | 0.00161 | 3037499 | A | G | 0.136865 | 1.22E-11 | 1.52E-05 | 46.17 |
| rs4798783 | -0.01152 | 0.00115 | 3037499 | A | G | 0.705405 | 1.26E-23 | 3.30E-05 | 100.35 |
| rs17257579 | -0.0131 | 0.00159 | 3037499 | T | C | 0.878703 | 1.40E-16 | 2.23E-05 | 67.88 |
| rs863006 | -0.00984 | 0.00106 | 3037499 | A | G | 0.558223 | 1.31E-20 | 2.84E-05 | 86.17 |
| rs489408 | 0.00766 | 0.00105 | 3037499 | A | G | 0.436326 | 3.25E-13 | 1.75E-05 | 53.22 |
| rs12709690 | -0.01858 | 0.00109 | 3037499 | T | C | 0.372621 | 9.28E-65 | 9.56E-05 | 290.56 |
| rs11196397 | 0.00897 | 0.00117 | 3037499 | A | C | 0.26158 | 2.22E-14 | 1.94E-05 | 58.78 |
| rs952062 | 0.01023 | 0.00169 | 3037499 | A | T | 0.127333 | 1.30E-09 | 1.21E-05 | 36.64 |
| rs7097348 | -0.00905 | 0.00116 | 3037499 | T | C | 0.724669 | 6.44E-15 | 2.00E-05 | 60.87 |
| rs2668196 | -0.01186 | 0.00132 | 3037499 | A | T | 0.207961 | 3.28E-19 | 2.66E-05 | 80.73 |
| rs28807201 | -0.01139 | 0.00114 | 3037499 | T | C | 0.684416 | 1.97E-23 | 3.29E-05 | 99.82 |
| rs7184863 | 0.00803 | 0.00136 | 3037499 | T | G | 0.802371 | 3.72E-09 | 1.15E-05 | 34.86 |
| rs738988 | -0.01605 | 0.00116 | 3037499 | A | G | 0.737681 | 1.36E-43 | 6.30E-05 | 191.44 |
| rs6713695 | -0.0106 | 0.00105 | 3037499 | A | G | 0.457699 | 5.18E-24 | 3.36E-05 | 101.91 |
| rs62439683 | -0.01207 | 0.00121 | 3037499 | A | C | 0.244071 | 1.55E-23 | 3.28E-05 | 99.5 |
| rs149285517 | -0.01187 | 0.00164 | 3037499 | T | C | 0.122698 | 4.61E-13 | 1.72E-05 | 52.39 |
| rs34743418 | -0.00999 | 0.00106 | 3037499 | T | C | 0.463998 | 5.01E-21 | 2.92E-05 | 88.82 |
| rs16983844 | 0.01074 | 0.00129 | 3037499 | A | G | 0.188266 | 7.86E-17 | 2.28E-05 | 69.32 |
| rs1043595 | 0.01663 | 0.00118 | 3037499 | A | G | 0.261241 | 2.36E-45 | 6.54E-05 | 198.62 |
| rs7578247 | -0.00798 | 0.00109 | 3037499 | A | G | 0.608978 | 2.25E-13 | 1.76E-05 | 53.6 |
| rs73219806 | 0.0124 | 0.00141 | 3037499 | A | C | 0.172267 | 1.34E-18 | 2.55E-05 | 77.34 |
| rs6907381 | -0.01185 | 0.00159 | 3037499 | A | G | 0.130644 | 9.09E-14 | 1.83E-05 | 55.54 |
| rs1193240 | 0.00847 | 0.00106 | 3037499 | A | C | 0.423252 | 1.73E-15 | 2.10E-05 | 63.85 |
| rs336433 | 0.00775 | 0.00105 | 3037499 | A | G | 0.421805 | 2.05E-13 | 1.79E-05 | 54.48 |
| rs3095075 | -0.0136 | 0.00105 | 3037499 | A | G | 0.550108 | 3.81E-38 | 5.52E-05 | 167.76 |
| rs62247449 | 0.00955 | 0.00106 | 3037499 | C | G | 0.411072 | 1.50E-19 | 2.67E-05 | 81.17 |
| rs2177083 | -0.00741 | 0.00105 | 3037499 | A | G | 0.50214 | 1.49E-12 | 1.64E-05 | 49.8 |
| rs13133213 | 0.00873 | 0.00105 | 3037499 | A | G | 0.502233 | 7.24E-17 | 2.28E-05 | 69.13 |
| rs3098650 | 0.01181 | 0.00108 | 3037499 | C | G | 0.602464 | 7.71E-28 | 3.94E-05 | 119.58 |
| rs10507085 | -0.01224 | 0.002 | 3037499 | T | C | 0.068063 | 9.14E-10 | 1.23E-05 | 37.45 |

**SNP**, Single nucleotide polymorphism; **Pos**, position for SNP; **Chr**, chromosome; **SE**, standard error; **EAF**, effect allele frequency; **P-value**, the value for the genetic association.

**Supplementary Table S4.** Characteristics of Townsend Deprivation Index-associated SNPs for IVs to atrial fibrillation in Nielsen et al.

| SNP | Chr | Pos | Beta | SE | Sample  size | Effect allele | Other allele | EAF | P_value | R2 | F |
| --- | --- | --- | --- | --- | --- | --- | --- | --- | --- | --- | --- |
| rs1947083 | 1 | 67004801 | 0.011866 | 0.002111 | 462464 | A | G | 0.641804 | 1.90E-08 | 6.84E-05 | 31.61 |
| rs56142341 | 1 | 208145995 | -0.0259 | 0.004433 | 462464 | C | G | 0.062465 | 5.20E-09 | 7.38E-05 | 34.12 |
| rs989532 | 2 | 173933824 | 0.013856 | 0.002128 | 462464 | G | A | 0.353176 | 7.40E-11 | 9.17E-05 | 42.41 |
| rs1483246 | 2 | 186050380 | -0.01211 | 0.002044 | 462464 | C | T | 0.555268 | 3.10E-09 | 7.59E-05 | 35.1 |
| rs78257128 | 5 | 164545854 | -0.01674 | 0.002807 | 462464 | T | C | 0.154162 | 2.40E-09 | 7.69E-05 | 35.58 |
| rs2403326 | 5 | 103791044 | 0.011547 | 0.002027 | 462464 | G | A | 0.486226 | 1.20E-08 | 7.02E-05 | 32.45 |
| rs253125 | 5 | 106645685 | 0.013174 | 0.002343 | 462464 | C | T | 0.749502 | 1.90E-08 | 6.83E-05 | 31.61 |
| rs7740440 | 6 | 142972917 | 0.011847 | 0.002034 | 462464 | A | G | 0.487004 | 5.70E-09 | 7.34E-05 | 33.93 |
| rs12203592 | 6 | 396321 | 0.017001 | 0.00241 | 462464 | T | C | 0.219642 | 1.70E-12 | 1.08E-04 | 49.77 |
| rs6931604 | 6 | 98578215 | -0.01219 | 0.002073 | 462464 | T | C | 0.600998 | 4.10E-09 | 7.48E-05 | 34.58 |
| rs62477310 | 7 | 114951541 | -0.01187 | 0.002035 | 462464 | C | T | 0.486627 | 5.40E-09 | 7.36E-05 | 34.02 |
| rs990706 | 11 | 78135704 | 0.015587 | 0.002701 | 462464 | T | C | 0.171143 | 7.90E-09 | 7.20E-05 | 33.29 |
| rs113345285 | 14 | 104246362 | 0.016313 | 0.002951 | 462464 | C | T | 0.136874 | 3.20E-08 | 6.61E-05 | 30.56 |
| rs4785187 | 16 | 49766772 | 0.0138 | 0.002437 | 462464 | A | G | 0.223024 | 1.50E-08 | 6.93E-05 | 32.06 |

**SNP**, Single nucleotide polymorphism; **Pos**, position for SNP; **Chr**, chromosome; **SE**, standard error; **EAF**, effect allele frequency; **P-value**, the value for the genetic association.

**Supplementary Table S5.** Characteristics of house income-associated SNPs for IVs to atrial fibrillation in Nielsen et al.

| SNP | Chr | Pos | Beta | SE | Sample  size | Effect allele | Other allele | EAF | P_value | R2 | F |
| --- | --- | --- | --- | --- | --- | --- | --- | --- | --- | --- | --- |
| rs11588857 | 1 | 2.05E+08 | 0.02134 | 0.003246 | 397751 | A | G | 0.209469 | 4.90E-11 | 1.09E-04 | 43.22 |
| rs488786 | 1 | 20888207 | 0.020688 | 0.003568 | 397751 | T | C | 0.164058 | 6.70E-09 | 8.45E-05 | 33.63 |
| rs6429636 | 1 | 44183540 | 0.019286 | 0.002935 | 397751 | T | G | 0.719691 | 5.00E-11 | 1.09E-04 | 43.17 |
| rs387780 | 2 | 32502495 | 0.01673 | 0.002827 | 397751 | C | T | 0.674498 | 3.30E-09 | 8.80E-05 | 35.01 |
| rs11678501 | 2 | 1.89E+08 | -0.05179 | 0.008516 | 397751 | C | T | 0.024727 | 1.20E-09 | 9.30E-05 | 36.98 |
| rs62183028 | 2 | 2.13E+08 | -0.01898 | 0.002861 | 397751 | T | G | 0.310562 | 3.20E-11 | 1.11E-04 | 44.02 |
| rs2332719 | 3 | 1.24E+08 | -0.0183 | 0.002953 | 397751 | G | A | 0.278917 | 5.70E-10 | 9.66E-05 | 38.42 |
| rs32940 | 5 | 1.41E+08 | 0.021143 | 0.002896 | 397751 | C | T | 0.700979 | 2.90E-13 | 1.34E-04 | 53.28 |
| rs6868457 | 5 | 60550041 | 0.02098 | 0.00266 | 397751 | C | T | 0.478238 | 3.10E-15 | 1.56E-04 | 62.2 |
| rs73015322 | 6 | 1.64E+08 | -0.02719 | 0.004954 | 397751 | T | G | 0.076734 | 4.10E-08 | 7.57E-05 | 30.12 |
| rs12531825 | 7 | 8005174 | -0.02582 | 0.004062 | 397751 | A | G | 0.122596 | 2.10E-10 | 1.02E-04 | 40.4 |
| rs77126132 | 7 | 54966738 | 0.026748 | 0.004579 | 397751 | A | G | 0.093289 | 5.20E-09 | 8.58E-05 | 34.12 |
| rs71576284 | 7 | 1.26E+08 | -0.08389 | 0.015177 | 397751 | A | C | 0.008961 | 3.30E-08 | 7.68E-05 | 30.55 |
| rs10761035 | 9 | 99236092 | 0.01864 | 0.003385 | 397751 | A | G | 0.188004 | 3.70E-08 | 7.62E-05 | 30.32 |
| rs2068428 | 9 | 1792147 | 0.016972 | 0.003095 | 397751 | T | C | 0.239041 | 4.20E-08 | 7.56E-05 | 30.07 |
| rs10429582 | 9 | 23346850 | 0.027026 | 0.002684 | 397751 | C | T | 0.416388 | 7.60E-24 | 2.55E-04 | 101.39 |
| rs34473884 | 10 | 1.34E+08 | 0.017225 | 0.003056 | 397751 | A | G | 0.248977 | 1.70E-08 | 7.99E-05 | 31.77 |
| rs589914 | 11 | 57661032 | 0.01561 | 0.002851 | 397751 | G | A | 0.684776 | 4.40E-08 | 7.54E-05 | 29.98 |
| rs9596313 | 13 | 51150873 | 0.015287 | 0.002648 | 397751 | A | G | 0.505975 | 7.80E-09 | 8.38E-05 | 33.34 |
| rs9556958 | 13 | 99100046 | -0.01534 | 0.002664 | 397751 | T | C | 0.524062 | 8.50E-09 | 8.34E-05 | 33.16 |
| rs11877758 | 18 | 35138110 | -0.02036 | 0.002867 | 397751 | G | T | 0.312055 | 1.20E-12 | 1.27E-04 | 50.43 |
| rs7227402 | 18 | 50895766 | -0.01696 | 0.002678 | 397751 | T | G | 0.423669 | 2.40E-10 | 1.01E-04 | 40.11 |
| rs75413320 | 19 | 13149854 | -0.02611 | 0.004268 | 397751 | C | T | 0.108161 | 9.50E-10 | 9.41E-05 | 37.43 |
| rs2362523 | 19 | 5002301 | 0.015867 | 0.002798 | 397751 | G | A | 0.339926 | 1.40E-08 | 8.08E-05 | 32.15 |
| rs6035877 | 20 | 21512532 | -0.01451 | 0.00265 | 397751 | C | A | 0.469244 | 4.30E-08 | 7.54E-05 | 29.99 |
| rs2422859 | 20 | 3132828 | 0.016371 | 0.002647 | 397751 | G | T | 0.471922 | 6.20E-10 | 9.61E-05 | 38.25 |
| rs5754738 | 22 | 34280249 | -0.01625 | 0.00292 | 397751 | G | A | 0.709551 | 2.70E-08 | 7.78E-05 | 30.95 |

**SNP**, Single nucleotide polymorphism; **Pos**, position for SNP; **Chr**, chromosome; **SE**, standard error; **EAF**, effect allele frequency; **P-value**, the value for the genetic association.

**Supplementary Table S6.** Characteristics of educational attainment-associated SNPs for IVs to atrial fibrillation in Nielsen et al.

| SNP | Beta | SE | Sample  size | Effect allele | Other allele | EAF | P_value | R2 | F |
| --- | --- | --- | --- | --- | --- | --- | --- | --- | --- |
| rs114440021 | 0.01233 | 0.00165 | 3037499 | T | C | 0.111056 | 7.00E-14 | 1.84E-05 | 55.84 |
| rs12589471 | -0.00798 | 0.00108 | 3037499 | A | T | 0.382014 | 1.19E-13 | 1.80E-05 | 54.6 |
| rs899223 | 0.01332 | 0.00193 | 3037499 | C | G | 0.078673 | 5.07E-12 | 1.57E-05 | 47.63 |
| rs421154 | 0.01098 | 0.00125 | 3037499 | C | G | 0.781367 | 2.06E-18 | 2.54E-05 | 77.16 |
| rs4766975 | -0.00871 | 0.00107 | 3037499 | A | G | 0.61526 | 4.40E-16 | 2.18E-05 | 66.26 |
| rs6539284 | -0.00721 | 0.00107 | 3037499 | T | C | 0.612997 | 1.38E-11 | 1.49E-05 | 45.4 |
| rs6563363 | -0.00921 | 0.00108 | 3037499 | T | G | 0.357053 | 1.70E-17 | 2.39E-05 | 72.72 |
| rs74740938 | 0.01208 | 0.00162 | 3037499 | A | T | 0.132445 | 8.30E-14 | 1.83E-05 | 55.6 |
| rs7301232 | -0.00773 | 0.0014 | 3037499 | A | T | 0.167863 | 3.80E-08 | 1.00E-05 | 30.49 |
| rs9551887 | -0.008 | 0.00105 | 3037499 | T | C | 0.531029 | 2.88E-14 | 1.91E-05 | 58.05 |
| rs11746390 | -0.0086 | 0.00118 | 3037499 | T | C | 0.742439 | 3.28E-13 | 1.75E-05 | 53.12 |
| rs211283 | -0.00922 | 0.00123 | 3037499 | C | G | 0.233123 | 5.18E-14 | 1.85E-05 | 56.19 |
| rs11633934 | 0.00803 | 0.00105 | 3037499 | A | C | 0.549938 | 2.10E-14 | 1.93E-05 | 58.49 |
| rs57214198 | -0.0155 | 0.00236 | 3037499 | T | G | 0.055159 | 5.47E-11 | 1.42E-05 | 43.14 |
| rs16919774 | -0.00935 | 0.00128 | 3037499 | A | T | 0.794657 | 2.67E-13 | 1.76E-05 | 53.36 |
| rs12524795 | 0.00851 | 0.00105 | 3037499 | T | C | 0.438482 | 6.32E-16 | 2.16E-05 | 65.69 |
| rs10180485 | 0.01188 | 0.0016 | 3037499 | T | G | 0.882753 | 1.29E-13 | 1.81E-05 | 55.13 |
| rs7070545 | -0.00624 | 0.00108 | 3037499 | A | G | 0.639067 | 8.89E-09 | 1.10E-05 | 33.38 |
| rs7924465 | 0.01448 | 0.0018 | 3037499 | T | C | 0.909409 | 8.50E-16 | 2.13E-05 | 64.71 |
| rs2055940 | 0.00703 | 0.00112 | 3037499 | A | G | 0.307207 | 3.32E-10 | 1.30E-05 | 39.4 |
| rs11942352 | -0.00894 | 0.00132 | 3037499 | A | G | 0.814075 | 1.40E-11 | 1.51E-05 | 45.87 |
| rs12065238 | 0.01009 | 0.00139 | 3037499 | A | G | 0.179812 | 4.57E-13 | 1.73E-05 | 52.69 |
| rs12142570 | 0.00999 | 0.0014 | 3037499 | A | T | 0.818525 | 1.08E-12 | 1.68E-05 | 50.92 |
| rs12974657 | 0.00917 | 0.00113 | 3037499 | T | C | 0.677318 | 3.71E-16 | 2.17E-05 | 65.85 |
| rs1866823 | 0.01058 | 0.00105 | 3037499 | A | G | 0.54247 | 8.10E-24 | 3.34E-05 | 101.53 |
| rs4741600 | 0.0158 | 0.0011 | 3037499 | T | C | 0.344949 | 2.09E-46 | 6.79E-05 | 206.31 |
| rs11614957 | 0.01206 | 0.00105 | 3037499 | A | G | 0.450693 | 2.82E-30 | 4.34E-05 | 131.92 |
| rs2222760 | -0.00794 | 0.00116 | 3037499 | A | G | 0.27219 | 8.24E-12 | 1.54E-05 | 46.85 |
| rs12240387 | -0.00599 | 0.00109 | 3037499 | A | G | 0.356391 | 3.59E-08 | 9.94E-06 | 30.2 |
| rs252991 | 0.01026 | 0.00108 | 3037499 | A | G | 0.369464 | 2.33E-21 | 2.97E-05 | 90.25 |
| rs13214394 | -0.00674 | 0.00121 | 3037499 | A | T | 0.767231 | 2.81E-08 | 1.02E-05 | 31.03 |
| rs2025258 | 0.00668 | 0.0011 | 3037499 | T | C | 0.657299 | 1.23E-09 | 1.21E-05 | 36.88 |
| rs8058137 | 0.01466 | 0.00127 | 3037499 | A | G | 0.772806 | 8.61E-31 | 4.39E-05 | 133.25 |
| rs1581168 | -0.01011 | 0.00116 | 3037499 | T | C | 0.295457 | 2.22E-18 | 2.50E-05 | 75.96 |
| rs660010 | 0.01863 | 0.0015 | 3037499 | C | G | 0.123945 | 3.24E-35 | 5.08E-05 | 154.26 |
| rs6728111 | 0.00609 | 0.00105 | 3037499 | A | G | 0.460148 | 6.47E-09 | 1.11E-05 | 33.64 |
| rs13332638 | -0.01304 | 0.00187 | 3037499 | A | G | 0.091777 | 3.12E-12 | 1.60E-05 | 48.63 |
| rs10752262 | 0.00932 | 0.00106 | 3037499 | T | C | 0.408516 | 2.08E-18 | 2.55E-05 | 77.31 |
| rs11158200 | 0.00861 | 0.00105 | 3037499 | A | G | 0.540468 | 2.53E-16 | 2.21E-05 | 67.24 |
| rs8072494 | -0.0116 | 0.00128 | 3037499 | A | G | 0.788728 | 1.65E-19 | 2.70E-05 | 82.13 |
| rs72906124 | 0.01836 | 0.00226 | 3037499 | T | C | 0.948337 | 4.94E-16 | 2.17E-05 | 66 |
| rs7027899 | 0.01137 | 0.00193 | 3037499 | A | G | 0.80907 | 3.80E-09 | 1.14E-05 | 34.71 |
| rs14184 | -0.01343 | 0.00114 | 3037499 | C | G | 0.702756 | 6.96E-32 | 4.57E-05 | 138.78 |
| rs2195041 | 0.00659 | 0.00107 | 3037499 | A | G | 0.392886 | 6.16E-10 | 1.25E-05 | 37.93 |
| rs4391653 | -0.00816 | 0.00119 | 3037499 | T | C | 0.254173 | 6.89E-12 | 1.55E-05 | 47.02 |
| rs12569163 | -0.01233 | 0.00183 | 3037499 | T | G | 0.800092 | 1.69E-11 | 1.49E-05 | 45.4 |
| rs7603132 | 0.01729 | 0.00134 | 3037499 | A | G | 0.183585 | 2.70E-38 | 5.48E-05 | 166.49 |
| rs4751360 | 0.0091 | 0.00105 | 3037499 | T | C | 0.468987 | 4.01E-18 | 2.47E-05 | 75.11 |
| rs12609965 | 0.00706 | 0.00122 | 3037499 | A | C | 0.245504 | 8.05E-09 | 1.10E-05 | 33.49 |
| rs6534704 | -0.015 | 0.00189 | 3037499 | A | T | 0.073221 | 1.96E-15 | 2.07E-05 | 62.99 |
| rs10772644 | 0.01397 | 0.00168 | 3037499 | C | G | 0.886665 | 1.05E-16 | 2.28E-05 | 69.15 |
| rs11081529 | 0.01228 | 0.00117 | 3037499 | T | C | 0.7247 | 7.10E-26 | 3.63E-05 | 110.16 |
| rs705240 | -0.01301 | 0.00135 | 3037499 | T | C | 0.181183 | 4.56E-22 | 3.06E-05 | 92.87 |
| rs4551987 | 0.01248 | 0.00142 | 3037499 | A | G | 0.839436 | 1.62E-18 | 2.54E-05 | 77.24 |
| rs4685448 | -0.00679 | 0.00105 | 3037499 | A | G | 0.421497 | 1.20E-10 | 1.38E-05 | 41.82 |
| rs13284474 | 0.00635 | 0.00105 | 3037499 | T | C | 0.427502 | 1.72E-09 | 1.20E-05 | 36.57 |
| rs10016110 | 0.00947 | 0.00108 | 3037499 | A | G | 0.369587 | 1.44E-18 | 2.53E-05 | 76.89 |
| rs56188374 | -0.01411 | 0.00193 | 3037499 | A | G | 0.070142 | 2.72E-13 | 1.76E-05 | 53.45 |
| rs2289769 | -0.00838 | 0.00151 | 3037499 | A | G | 0.869002 | 2.83E-08 | 1.01E-05 | 30.8 |
| rs41741 | 0.00737 | 0.00111 | 3037499 | T | G | 0.663074 | 3.35E-11 | 1.45E-05 | 44.08 |
| rs6044142 | -0.00739 | 0.00106 | 3037499 | A | G | 0.584339 | 2.65E-12 | 1.60E-05 | 48.6 |
| rs5759002 | 0.00832 | 0.00105 | 3037499 | A | G | 0.507638 | 1.98E-15 | 2.07E-05 | 62.79 |
| rs35565533 | 0.00842 | 0.00119 | 3037499 | A | C | 0.744225 | 1.22E-12 | 1.65E-05 | 50.06 |
| rs67829508 | -0.0092 | 0.00136 | 3037499 | A | G | 0.175978 | 1.16E-11 | 1.51E-05 | 45.76 |
| rs111821073 | 0.01533 | 0.00145 | 3037499 | T | C | 0.149061 | 4.70E-26 | 3.68E-05 | 111.78 |
| rs62262312 | 0.01889 | 0.00142 | 3037499 | C | G | 0.150724 | 2.36E-40 | 5.83E-05 | 176.96 |
| rs1337731 | 0.00863 | 0.00111 | 3037499 | A | G | 0.340345 | 7.07E-15 | 1.99E-05 | 60.45 |
| rs28617748 | 0.00909 | 0.00128 | 3037499 | A | T | 0.996905 | 1.06E-12 | 1.66E-05 | 50.43 |
| rs2301015 | -0.01065 | 0.00112 | 3037499 | T | C | 0.341084 | 1.37E-21 | 2.98E-05 | 90.42 |
| rs4772268 | 0.00992 | 0.00111 | 3037499 | A | G | 0.332799 | 4.03E-19 | 2.63E-05 | 79.87 |
| rs7632819 | -0.01301 | 0.00122 | 3037499 | A | G | 0.76138 | 1.13E-26 | 3.74E-05 | 113.72 |
| rs13252156 | 0.00899 | 0.00107 | 3037499 | A | C | 0.416954 | 4.69E-17 | 2.32E-05 | 70.59 |
| rs1182532 | 0.01107 | 0.00146 | 3037499 | A | C | 0.145642 | 4.00E-14 | 1.89E-05 | 57.49 |
| rs11172371 | -0.0107 | 0.00112 | 3037499 | T | C | 0.313705 | 1.23E-21 | 3.00E-05 | 91.27 |
| rs10167909 | 0.00897 | 0.00127 | 3037499 | T | C | 0.197244 | 1.98E-12 | 1.64E-05 | 49.89 |
| rs118134876 | -0.03109 | 0.00219 | 3037499 | T | C | 0.053449 | 6.97E-46 | 6.63E-05 | 201.54 |
| rs9374194 | -0.00881 | 0.00133 | 3037499 | T | C | 0.808593 | 4.06E-11 | 1.44E-05 | 43.88 |
| rs567775 | 0.00697 | 0.00105 | 3037499 | T | C | 0.476794 | 2.73E-11 | 1.45E-05 | 44.06 |
| rs115124024 | 0.01783 | 0.00241 | 3037499 | A | C | 0.952649 | 1.47E-13 | 1.80E-05 | 54.74 |
| rs7109373 | 0.01041 | 0.00132 | 3037499 | A | T | 0.805929 | 2.90E-15 | 2.05E-05 | 62.19 |
| rs12699131 | 0.01382 | 0.00105 | 3037499 | A | G | 0.471158 | 1.05E-39 | 5.70E-05 | 173.24 |
| rs11861256 | 0.01004 | 0.00133 | 3037499 | A | C | 0.191839 | 4.73E-14 | 1.88E-05 | 56.99 |
| rs8020023 | 0.01445 | 0.00123 | 3037499 | A | G | 0.247182 | 6.34E-32 | 4.54E-05 | 138.01 |
| rs4141463 | -0.00864 | 0.00106 | 3037499 | T | C | 0.429104 | 3.90E-16 | 2.19E-05 | 66.44 |
| rs7296742 | -0.00855 | 0.00116 | 3037499 | A | G | 0.274145 | 2.06E-13 | 1.79E-05 | 54.33 |
| rs77584294 | 0.02591 | 0.00268 | 3037499 | A | C | 0.03725 | 3.86E-22 | 3.08E-05 | 93.47 |
| rs12908446 | 0.00726 | 0.00128 | 3037499 | T | C | 0.780828 | 1.59E-08 | 1.06E-05 | 32.17 |
| rs6747129 | -0.01568 | 0.00173 | 3037499 | A | G | 0.119495 | 1.14E-19 | 2.70E-05 | 82.15 |
| rs12339610 | 0.00904 | 0.0012 | 3037499 | T | C | 0.745827 | 4.08E-14 | 1.87E-05 | 56.75 |
| rs78410329 | 0.01783 | 0.00126 | 3037499 | T | C | 0.237404 | 1.63E-45 | 6.59E-05 | 200.24 |
| rs2303907 | 0.00852 | 0.00105 | 3037499 | T | C | 0.517847 | 4.15E-16 | 2.17E-05 | 65.84 |
| rs9540920 | 0.00584 | 0.00106 | 3037499 | A | G | 0.578703 | 3.38E-08 | 9.99E-06 | 30.35 |
| rs2974337 | 0.0118 | 0.00105 | 3037499 | T | C | 0.478134 | 2.42E-29 | 4.16E-05 | 126.29 |
| rs7977614 | -0.01115 | 0.00118 | 3037499 | A | G | 0.725085 | 2.53E-21 | 2.94E-05 | 89.29 |
| rs1464018 | -0.01361 | 0.00128 | 3037499 | T | C | 0.245288 | 2.81E-26 | 3.72E-05 | 113.06 |
| rs6721505 | 0.00736 | 0.00112 | 3037499 | A | G | 0.324669 | 4.09E-11 | 1.42E-05 | 43.18 |
| rs75756477 | 0.01406 | 0.00255 | 3037499 | T | C | 0.961287 | 3.49E-08 | 1.00E-05 | 30.4 |
| rs10745307 | -0.01069 | 0.00105 | 3037499 | A | G | 0.564829 | 2.85E-24 | 3.41E-05 | 103.65 |
| rs78116078 | 0.0093 | 0.00116 | 3037499 | C | G | 0.726332 | 1.28E-15 | 2.12E-05 | 64.28 |
| rs73210523 | 0.01184 | 0.00138 | 3037499 | A | G | 0.164906 | 9.72E-18 | 2.42E-05 | 73.61 |
| rs12828220 | -0.00975 | 0.00109 | 3037499 | T | C | 0.598352 | 2.79E-19 | 2.63E-05 | 80.01 |
| rs6547396 | 0.00772 | 0.00107 | 3037499 | T | C | 0.579674 | 4.49E-13 | 1.71E-05 | 52.06 |
| rs2371001 | -0.00929 | 0.00105 | 3037499 | A | G | 0.500601 | 7.15E-19 | 2.58E-05 | 78.28 |
| rs561655 | -0.00891 | 0.0011 | 3037499 | A | G | 0.653403 | 5.69E-16 | 2.16E-05 | 65.61 |
| rs7788620 | 0.0167 | 0.00126 | 3037499 | A | G | 0.785541 | 8.27E-40 | 5.78E-05 | 175.67 |
| rs4846724 | 0.00767 | 0.00105 | 3037499 | A | G | 0.531722 | 2.62E-13 | 1.76E-05 | 53.36 |
| rs17266097 | 0.00879 | 0.00107 | 3037499 | T | C | 0.385679 | 2.00E-16 | 2.22E-05 | 67.49 |
| rs1585524 | 0.00576 | 0.00106 | 3037499 | A | G | 0.473314 | 4.66E-08 | 9.72E-06 | 29.53 |
| rs80171383 | 0.01462 | 0.00153 | 3037499 | A | G | 0.125085 | 1.18E-21 | 3.01E-05 | 91.31 |
| rs36048136 | 0.01179 | 0.00125 | 3037499 | A | G | 0.207607 | 4.70E-21 | 2.93E-05 | 88.96 |
| rs2364972 | 0.00719 | 0.00105 | 3037499 | A | G | 0.548476 | 7.72E-12 | 1.54E-05 | 46.89 |
| rs1387148 | -0.00946 | 0.00131 | 3037499 | A | C | 0.192809 | 5.94E-13 | 1.72E-05 | 52.15 |
| rs2964252 | 0.01024 | 0.00113 | 3037499 | A | G | 0.321605 | 1.30E-19 | 2.70E-05 | 82.12 |
| rs7150195 | 0.01365 | 0.00108 | 3037499 | T | G | 0.365306 | 1.44E-36 | 5.26E-05 | 159.74 |
| rs10481106 | -0.01237 | 0.00144 | 3037499 | A | G | 0.156283 | 8.90E-18 | 2.43E-05 | 73.79 |
| rs3013170 | 0.00707 | 0.00107 | 3037499 | T | C | 0.596427 | 4.53E-11 | 1.44E-05 | 43.66 |
| rs10887465 | 0.01276 | 0.00118 | 3037499 | A | C | 0.274546 | 4.91E-27 | 3.85E-05 | 116.93 |
| rs1039617 | -0.00785 | 0.00106 | 3037499 | T | C | 0.40907 | 1.41E-13 | 1.81E-05 | 54.84 |
| rs192230289 | -0.02665 | 0.00406 | 3037499 | T | C | 0.016307 | 5.24E-11 | 1.42E-05 | 43.09 |
| rs72748123 | -0.0118 | 0.00142 | 3037499 | A | G | 0.15967 | 7.74E-17 | 2.27E-05 | 69.05 |
| rs12893970 | -0.01272 | 0.00176 | 3037499 | A | G | 0.087219 | 4.76E-13 | 1.72E-05 | 52.23 |
| rs4879832 | -0.00637 | 0.00108 | 3037499 | T | C | 0.391423 | 3.56E-09 | 1.15E-05 | 34.79 |
| rs13138842 | 0.00834 | 0.00132 | 3037499 | A | G | 0.193224 | 2.70E-10 | 1.31E-05 | 39.92 |
| rs2295230 | 0.01399 | 0.00115 | 3037499 | A | C | 0.687111 | 5.63E-34 | 4.87E-05 | 147.99 |
| rs2154209 | -0.00779 | 0.00131 | 3037499 | T | C | 0.800832 | 2.51E-09 | 1.16E-05 | 35.36 |
| rs71366589 | 0.02255 | 0.00373 | 3037499 | T | C | 0.983862 | 1.46E-09 | 1.20E-05 | 36.55 |
| rs623200 | 0.00704 | 0.00108 | 3037499 | T | C | 0.398322 | 5.79E-11 | 1.40E-05 | 42.49 |
| rs6839051 | -0.0069 | 0.00108 | 3037499 | A | C | 0.383262 | 1.44E-10 | 1.34E-05 | 40.82 |
| rs55640000 | -0.00774 | 0.00109 | 3037499 | T | G | 0.633985 | 1.38E-12 | 1.66E-05 | 50.42 |
| rs4812818 | -0.00739 | 0.00132 | 3037499 | T | G | 0.184108 | 2.23E-08 | 1.03E-05 | 31.34 |
| rs13212041 | 0.01123 | 0.00131 | 3037499 | T | C | 0.799707 | 9.06E-18 | 2.42E-05 | 73.49 |
| rs56409354 | -0.01599 | 0.00127 | 3037499 | A | G | 0.218232 | 1.64E-36 | 5.22E-05 | 158.52 |
| rs7009856 | 0.00671 | 0.00107 | 3037499 | C | G | 0.403711 | 4.12E-10 | 1.29E-05 | 39.33 |
| rs145481578 | 0.02354 | 0.00386 | 3037499 | T | C | 0.019341 | 1.09E-09 | 1.22E-05 | 37.19 |
| rs2498018 | -0.00893 | 0.00133 | 3037499 | C | G | 0.186464 | 1.92E-11 | 1.48E-05 | 45.08 |
| rs13159124 | -0.01127 | 0.00112 | 3037499 | A | G | 0.692224 | 1.01E-23 | 3.33E-05 | 101.25 |
| rs5021426 | -0.01116 | 0.00117 | 3037499 | T | C | 0.716215 | 1.66E-21 | 3.00E-05 | 90.98 |
| rs7032484 | 0.01301 | 0.00106 | 3037499 | A | G | 0.56854 | 6.76E-35 | 4.96E-05 | 150.64 |
| rs42210 | -0.01177 | 0.00116 | 3037499 | C | G | 0.691685 | 3.13E-24 | 3.39E-05 | 102.95 |
| rs6731373 | -0.00872 | 0.0011 | 3037499 | A | G | 0.320296 | 2.64E-15 | 2.07E-05 | 62.84 |
| rs10739569 | -0.00723 | 0.00114 | 3037499 | C | G | 0.696458 | 2.49E-10 | 1.32E-05 | 40.22 |
| rs28381527 | -0.01999 | 0.00195 | 3037499 | A | G | 0.076948 | 9.74E-25 | 3.46E-05 | 105.09 |
| rs137121 | 0.01266 | 0.00157 | 3037499 | T | C | 0.126809 | 8.12E-16 | 2.14E-05 | 65.02 |
| rs7338804 | -0.0084 | 0.0015 | 3037499 | A | G | 0.143363 | 2.27E-08 | 1.03E-05 | 31.36 |
| rs4876775 | -0.01126 | 0.00113 | 3037499 | A | G | 0.337943 | 1.65E-23 | 3.27E-05 | 99.29 |
| rs7983020 | -0.00893 | 0.00116 | 3037499 | T | C | 0.703157 | 1.34E-14 | 1.95E-05 | 59.26 |
| rs989996 | -0.00942 | 0.00105 | 3037499 | T | C | 0.439205 | 2.98E-19 | 2.65E-05 | 80.49 |
| rs6596534 | 0.00622 | 0.00113 | 3037499 | A | G | 0.316646 | 4.11E-08 | 9.97E-06 | 30.3 |
| rs4864881 | -0.00698 | 0.00107 | 3037499 | T | C | 0.596397 | 7.74E-11 | 1.40E-05 | 42.55 |
| rs56194430 | -0.01861 | 0.00141 | 3037499 | T | C | 0.16081 | 5.72E-40 | 5.73E-05 | 174.2 |
| rs586829 | 0.00992 | 0.00108 | 3037499 | A | G | 0.634047 | 5.49E-20 | 2.78E-05 | 84.37 |
| rs7762296 | -0.00835 | 0.00114 | 3037499 | T | C | 0.312165 | 2.02E-13 | 1.77E-05 | 53.65 |
| rs73117392 | -0.02312 | 0.00228 | 3037499 | T | C | 0.948168 | 3.56E-24 | 3.39E-05 | 102.83 |
| rs11925699 | -0.00766 | 0.00105 | 3037499 | A | G | 0.463366 | 2.73E-13 | 1.75E-05 | 53.22 |
| rs10874759 | 0.01289 | 0.0011 | 3037499 | A | G | 0.652741 | 1.08E-31 | 4.52E-05 | 137.32 |
| rs7469569 | 0.00953 | 0.0011 | 3037499 | T | C | 0.346058 | 3.57E-18 | 2.47E-05 | 75.06 |
| rs17881016 | -0.01314 | 0.0012 | 3037499 | A | G | 0.742408 | 5.27E-28 | 3.95E-05 | 119.9 |
| rs10819974 | -0.00815 | 0.00123 | 3037499 | T | C | 0.222836 | 3.95E-11 | 1.45E-05 | 43.9 |
| rs62079997 | -0.00677 | 0.00106 | 3037499 | T | C | 0.543717 | 1.50E-10 | 1.34E-05 | 40.79 |
| rs11019128 | -0.00871 | 0.00108 | 3037499 | T | C | 0.359116 | 6.35E-16 | 2.14E-05 | 65.04 |
| rs9317202 | 0.00979 | 0.00114 | 3037499 | T | C | 0.698845 | 1.27E-17 | 2.43E-05 | 73.75 |
| rs12055782 | -0.01178 | 0.00116 | 3037499 | A | G | 0.725069 | 4.15E-24 | 3.40E-05 | 103.13 |
| rs57349798 | 0.0095 | 0.00106 | 3037499 | A | G | 0.39886 | 4.32E-19 | 2.64E-05 | 80.32 |
| rs7312511 | -0.00978 | 0.00176 | 3037499 | T | C | 0.104697 | 2.55E-08 | 1.02E-05 | 30.88 |
| rs419438 | -0.0071 | 0.00105 | 3037499 | T | G | 0.539021 | 1.41E-11 | 1.51E-05 | 45.72 |
| rs6534338 | 0.01047 | 0.00114 | 3037499 | T | C | 0.297644 | 4.14E-20 | 2.78E-05 | 84.35 |
| rs139872934 | 0.0138 | 0.00182 | 3037499 | T | C | 0.09421 | 3.17E-14 | 1.89E-05 | 57.49 |
| rs795540 | -0.00798 | 0.00106 | 3037499 | T | C | 0.590622 | 5.95E-14 | 1.87E-05 | 56.68 |
| rs11724690 | 0.00949 | 0.00115 | 3037499 | T | G | 0.282861 | 1.45E-16 | 2.24E-05 | 68.1 |
| rs8044562 | -0.01193 | 0.00116 | 3037499 | A | G | 0.276794 | 8.34E-25 | 3.48E-05 | 105.77 |
| rs16962221 | 0.00748 | 0.0011 | 3037499 | A | G | 0.350955 | 1.08E-11 | 1.52E-05 | 46.24 |
| rs184654 | 0.0165 | 0.00143 | 3037499 | C | G | 0.838451 | 1.13E-30 | 4.38E-05 | 133.14 |
| rs2160514 | -0.01111 | 0.00105 | 3037499 | A | C | 0.567508 | 5.90E-26 | 3.69E-05 | 111.96 |
| rs424387 | -0.00617 | 0.00107 | 3037499 | C | G | 0.598583 | 7.09E-09 | 1.09E-05 | 33.25 |
| rs13010288 | 0.0201 | 0.00157 | 3037499 | T | G | 0.123314 | 1.30E-37 | 5.40E-05 | 163.91 |
| rs9454264 | 0.00777 | 0.00105 | 3037499 | T | C | 0.468155 | 1.42E-13 | 1.80E-05 | 54.76 |
| rs13164530 | -0.01008 | 0.00155 | 3037499 | T | G | 0.134155 | 8.20E-11 | 1.39E-05 | 42.29 |
| rs114468556 | 0.02949 | 0.00261 | 3037499 | A | T | 0.03708 | 1.22E-29 | 4.20E-05 | 127.66 |
| rs11159067 | 0.00948 | 0.00111 | 3037499 | A | G | 0.657545 | 1.84E-17 | 2.40E-05 | 72.94 |
| rs72915557 | -0.02057 | 0.00221 | 3037499 | A | G | 0.051971 | 1.16E-20 | 2.85E-05 | 86.63 |
| rs34719425 | 0.00951 | 0.00122 | 3037499 | T | C | 0.25097 | 8.07E-15 | 2.00E-05 | 60.76 |
| rs4390219 | -0.00748 | 0.00113 | 3037499 | T | C | 0.31409 | 3.37E-11 | 1.44E-05 | 43.82 |
| rs150121450 | 0.01966 | 0.00304 | 3037499 | T | G | 0.028519 | 1.02E-10 | 1.38E-05 | 41.82 |
| rs1156541 | 0.00705 | 0.00125 | 3037499 | T | C | 0.763335 | 1.51E-08 | 1.05E-05 | 31.81 |
| rs12362273 | -0.00708 | 0.00123 | 3037499 | A | C | 0.771743 | 7.89E-09 | 1.09E-05 | 33.13 |
| rs1392816 | 0.00959 | 0.00108 | 3037499 | T | C | 0.371528 | 5.42E-19 | 2.60E-05 | 78.85 |
| rs10193498 | 0.01127 | 0.0012 | 3037499 | A | T | 0.730536 | 7.89E-21 | 2.90E-05 | 88.2 |
| rs117005905 | 0.01558 | 0.00162 | 3037499 | T | C | 0.115491 | 5.63E-22 | 3.04E-05 | 92.49 |
| rs11752345 | 0.00885 | 0.00128 | 3037499 | T | C | 0.205236 | 4.33E-12 | 1.57E-05 | 47.8 |
| rs62367470 | 0.00799 | 0.00137 | 3037499 | T | C | 0.1838 | 5.83E-09 | 1.12E-05 | 34.01 |
| rs9537509 | -0.00701 | 0.0012 | 3037499 | T | C | 0.744256 | 5.32E-09 | 1.12E-05 | 34.13 |
| rs10887592 | 0.00652 | 0.00118 | 3037499 | A | G | 0.256452 | 3.34E-08 | 1.01E-05 | 30.53 |
| rs4869579 | 0.00634 | 0.00105 | 3037499 | T | C | 0.515645 | 1.44E-09 | 1.20E-05 | 36.46 |
| rs1125854 | -0.00773 | 0.00108 | 3037499 | T | C | 0.361334 | 8.96E-13 | 1.69E-05 | 51.23 |
| rs9635366 | 0.0161 | 0.00136 | 3037499 | A | G | 0.211041 | 1.98E-32 | 4.61E-05 | 140.14 |
| rs3863241 | -0.00859 | 0.00105 | 3037499 | T | C | 0.536187 | 2.86E-16 | 2.20E-05 | 66.93 |
| rs6020560 | -0.00957 | 0.00105 | 3037499 | T | C | 0.51389 | 6.94E-20 | 2.73E-05 | 83.07 |
| rs590013 | 0.01107 | 0.00111 | 3037499 | T | C | 0.650293 | 2.96E-23 | 3.27E-05 | 99.46 |
| rs3796432 | 0.00684 | 0.00108 | 3037499 | T | G | 0.365938 | 2.67E-10 | 1.32E-05 | 40.11 |
| rs2063569 | -0.00962 | 0.00108 | 3037499 | C | G | 0.373745 | 6.08E-19 | 2.61E-05 | 79.34 |
| rs903981 | -0.00715 | 0.00106 | 3037499 | T | G | 0.431414 | 1.73E-11 | 1.50E-05 | 45.5 |
| rs56306348 | 0.00748 | 0.00108 | 3037499 | A | G | 0.624761 | 3.78E-12 | 1.58E-05 | 47.97 |
| rs7172133 | 0.00742 | 0.00113 | 3037499 | A | C | 0.688605 | 4.92E-11 | 1.42E-05 | 43.12 |
| rs4467547 | 0.01322 | 0.00107 | 3037499 | T | G | 0.384416 | 3.44E-35 | 5.03E-05 | 152.65 |
| rs2795022 | 0.00581 | 0.00107 | 3037499 | T | C | 0.391176 | 4.97E-08 | 9.71E-06 | 29.48 |
| rs356903 | -0.00719 | 0.00121 | 3037499 | T | C | 0.266615 | 2.46E-09 | 1.16E-05 | 35.31 |
| rs35414043 | -0.01539 | 0.00197 | 3037499 | A | G | 0.077348 | 5.58E-15 | 2.01E-05 | 61.03 |
| rs78462816 | -0.01037 | 0.00162 | 3037499 | A | G | 0.887558 | 1.42E-10 | 1.35E-05 | 40.98 |
| rs1483148 | 0.01209 | 0.00118 | 3037499 | T | G | 0.719048 | 1.28E-24 | 3.46E-05 | 104.98 |
| rs7027019 | -0.00618 | 0.00112 | 3037499 | T | C | 0.677271 | 3.26E-08 | 1.00E-05 | 30.45 |
| rs9871964 | -0.00951 | 0.00138 | 3037499 | T | C | 0.181013 | 5.42E-12 | 1.56E-05 | 47.49 |
| rs382196 | -0.00703 | 0.00108 | 3037499 | T | G | 0.360841 | 7.53E-11 | 1.39E-05 | 42.37 |
| rs28588750 | 0.01146 | 0.00109 | 3037499 | A | G | 0.654881 | 7.58E-26 | 3.64E-05 | 110.54 |
| rs12113634 | 0.00908 | 0.00109 | 3037499 | T | C | 0.65 | 9.23E-17 | 2.28E-05 | 69.39 |
| rs112780312 | -0.0096 | 0.00116 | 3037499 | A | G | 0.283508 | 1.10E-16 | 2.25E-05 | 68.49 |
| rs77187837 | 0.01293 | 0.0016 | 3037499 | T | C | 0.122713 | 5.83E-16 | 2.15E-05 | 65.31 |
| rs1128687 | -0.00813 | 0.00106 | 3037499 | T | C | 0.59247 | 1.75E-14 | 1.94E-05 | 58.83 |
| rs4883624 | -0.00689 | 0.00105 | 3037499 | T | C | 0.465953 | 5.98E-11 | 1.42E-05 | 43.06 |
| rs1866710 | -0.01449 | 0.00116 | 3037499 | A | G | 0.285987 | 1.07E-35 | 5.14E-05 | 156.03 |
| rs6898748 | -0.00661 | 0.00105 | 3037499 | T | C | 0.56024 | 3.58E-10 | 1.30E-05 | 39.63 |
| rs1291865 | -0.01031 | 0.00105 | 3037499 | T | G | 0.53785 | 6.83E-23 | 3.17E-05 | 96.41 |
| rs6428152 | -0.00805 | 0.00117 | 3037499 | A | G | 0.290037 | 5.49E-12 | 1.56E-05 | 47.34 |
| rs7668960 | 0.01587 | 0.00145 | 3037499 | T | C | 0.17028 | 5.42E-28 | 3.94E-05 | 119.79 |
| rs1006749 | 0.0063 | 0.00105 | 3037499 | A | G | 0.536064 | 2.04E-09 | 1.19E-05 | 36 |
| rs72695265 | -0.00962 | 0.00124 | 3037499 | T | C | 0.225778 | 1.13E-14 | 1.98E-05 | 60.19 |
| rs80049472 | -0.00963 | 0.00169 | 3037499 | A | G | 0.103942 | 1.17E-08 | 1.07E-05 | 32.47 |
| rs28834466 | 0.01325 | 0.00203 | 3037499 | A | G | 0.919526 | 6.56E-11 | 1.40E-05 | 42.6 |
| rs10732635 | 0.00697 | 0.00105 | 3037499 | T | G | 0.551293 | 3.77E-11 | 1.45E-05 | 44.06 |
| rs1278537 | -0.00674 | 0.00105 | 3037499 | T | G | 0.478318 | 1.24E-10 | 1.36E-05 | 41.2 |
| rs72625841 | 0.00865 | 0.00119 | 3037499 | T | G | 0.714937 | 3.78E-13 | 1.74E-05 | 52.84 |
| rs7254263 | -0.00993 | 0.00115 | 3037499 | T | C | 0.29498 | 7.90E-18 | 2.45E-05 | 74.56 |
| rs11772108 | -0.0082 | 0.00117 | 3037499 | A | T | 0.7172 | 2.44E-12 | 1.62E-05 | 49.12 |
| rs79925479 | 0.01354 | 0.00229 | 3037499 | T | G | 0.052079 | 3.64E-09 | 1.15E-05 | 34.96 |
| rs12897763 | 0.01968 | 0.00133 | 3037499 | A | G | 0.189159 | 1.73E-49 | 7.21E-05 | 218.95 |
| rs67033024 | 0.00934 | 0.00153 | 3037499 | A | G | 0.847598 | 9.77E-10 | 1.23E-05 | 37.27 |
| rs2631535 | 0.0123 | 0.00111 | 3037499 | A | G | 0.336141 | 2.44E-28 | 4.04E-05 | 122.79 |
| rs12150824 | 0.00718 | 0.00119 | 3037499 | A | G | 0.255205 | 1.62E-09 | 1.20E-05 | 36.4 |
| rs1329125 | -0.01243 | 0.00112 | 3037499 | T | C | 0.308208 | 1.06E-28 | 4.05E-05 | 123.17 |
| rs2630767 | 0.013 | 0.00105 | 3037499 | T | C | 0.474376 | 6.56E-35 | 5.05E-05 | 153.29 |
| rs2614463 | -0.0099 | 0.00106 | 3037499 | A | T | 0.611441 | 1.32E-20 | 2.87E-05 | 87.23 |
| rs12632133 | 0.01129 | 0.002 | 3037499 | A | G | 0.077903 | 1.57E-08 | 1.05E-05 | 31.87 |
| rs7657329 | -0.00856 | 0.00112 | 3037499 | A | C | 0.304512 | 2.59E-14 | 1.92E-05 | 58.41 |
| rs80352808 | -0.00751 | 0.00116 | 3037499 | A | G | 0.286803 | 1.15E-10 | 1.38E-05 | 41.91 |
| rs4667878 | 0.00757 | 0.00134 | 3037499 | A | T | 0.186126 | 1.66E-08 | 1.05E-05 | 31.91 |
| rs6466819 | -0.01031 | 0.00109 | 3037499 | A | G | 0.370696 | 2.49E-21 | 2.95E-05 | 89.47 |
| rs17503473 | 0.01194 | 0.00114 | 3037499 | T | C | 0.705266 | 1.13E-25 | 3.61E-05 | 109.7 |
| rs9922788 | 0.01028 | 0.00106 | 3037499 | A | G | 0.408331 | 2.12E-22 | 3.10E-05 | 94.05 |
| rs10865834 | -0.01049 | 0.00121 | 3037499 | A | G | 0.253896 | 3.58E-18 | 2.47E-05 | 75.16 |
| rs2884364 | -0.01595 | 0.00108 | 3037499 | A | G | 0.374299 | 1.29E-49 | 7.18E-05 | 218.11 |
| rs12155540 | 0.01172 | 0.00111 | 3037499 | T | C | 0.329889 | 6.83E-26 | 3.67E-05 | 111.48 |
| rs62114178 | 0.00685 | 0.00108 | 3037499 | A | G | 0.360333 | 2.49E-10 | 1.32E-05 | 40.23 |
| rs11664320 | 0.01759 | 0.00106 | 3037499 | T | C | 0.591346 | 2.21E-62 | 9.06E-05 | 275.37 |
| rs7356921 | -0.01482 | 0.00122 | 3037499 | T | C | 0.24595 | 9.09E-34 | 4.86E-05 | 147.56 |
| rs7905026 | -0.00748 | 0.00119 | 3037499 | A | C | 0.286203 | 3.62E-10 | 1.30E-05 | 39.51 |
| rs2026037 | 0.01545 | 0.00143 | 3037499 | T | C | 0.848753 | 4.56E-27 | 3.84E-05 | 116.73 |
| rs171697 | 0.01517 | 0.00111 | 3037499 | C | G | 0.687604 | 2.37E-42 | 6.15E-05 | 186.78 |
| rs35583726 | 0.01185 | 0.0014 | 3037499 | A | G | 0.836372 | 3.13E-17 | 2.36E-05 | 71.64 |
| rs1861786 | -0.00764 | 0.00108 | 3037499 | A | G | 0.376948 | 1.31E-12 | 1.65E-05 | 50.04 |
| rs11181124 | -0.00825 | 0.00128 | 3037499 | T | C | 0.797351 | 1.06E-10 | 1.37E-05 | 41.54 |
| rs10763979 | 0.0092 | 0.00108 | 3037499 | A | G | 0.61546 | 1.48E-17 | 2.39E-05 | 72.57 |
| rs1058790 | -0.01396 | 0.00136 | 3037499 | A | G | 0.806021 | 1.37E-24 | 3.47E-05 | 105.36 |
| rs1786313 | 0.00771 | 0.00113 | 3037499 | T | C | 0.308885 | 8.15E-12 | 1.53E-05 | 46.55 |
| rs2841425 | -0.00853 | 0.00137 | 3037499 | A | C | 0.180413 | 4.43E-10 | 1.28E-05 | 38.77 |
| rs7936656 | -0.00833 | 0.00112 | 3037499 | A | G | 0.313813 | 1.13E-13 | 1.82E-05 | 55.32 |
| rs9914251 | 0.0077 | 0.00122 | 3037499 | A | G | 0.750616 | 2.97E-10 | 1.31E-05 | 39.83 |
| rs6499084 | 0.00721 | 0.0011 | 3037499 | A | G | 0.353911 | 5.56E-11 | 1.41E-05 | 42.96 |
| rs6911986 | -0.00828 | 0.00118 | 3037499 | T | C | 0.268833 | 2.46E-12 | 1.62E-05 | 49.24 |
| rs9993133 | 0.00761 | 0.00138 | 3037499 | T | C | 0.810132 | 3.50E-08 | 1.00E-05 | 30.41 |
| rs77882218 | -0.02563 | 0.00315 | 3037499 | T | C | 0.969603 | 4.06E-16 | 2.18E-05 | 66.2 |
| rs1485272 | -0.00718 | 0.0011 | 3037499 | T | C | 0.644888 | 7.17E-11 | 1.40E-05 | 42.61 |
| rs17563464 | -0.01268 | 0.00127 | 3037499 | A | C | 0.202525 | 2.35E-23 | 3.28E-05 | 99.69 |
| rs60053512 | 0.01233 | 0.00144 | 3037499 | A | G | 0.167447 | 1.28E-17 | 2.41E-05 | 73.32 |
| rs1971218 | 0.00853 | 0.00105 | 3037499 | A | G | 0.51911 | 5.79E-16 | 2.17E-05 | 66 |
| rs10014934 | 0.00913 | 0.00108 | 3037499 | T | C | 0.611318 | 3.09E-17 | 2.35E-05 | 71.47 |
| rs4650228 | -0.00847 | 0.00105 | 3037499 | A | G | 0.462196 | 6.69E-16 | 2.14E-05 | 65.07 |
| rs12768641 | 0.0094 | 0.00123 | 3037499 | A | G | 0.767447 | 1.99E-14 | 1.92E-05 | 58.4 |
| rs28561815 | 0.00676 | 0.00116 | 3037499 | T | G | 0.301432 | 4.89E-09 | 1.12E-05 | 33.96 |
| rs7236339 | -0.01531 | 0.00127 | 3037499 | A | G | 0.203419 | 1.57E-33 | 4.78E-05 | 145.33 |
| rs2570492 | 0.01299 | 0.00107 | 3037499 | A | G | 0.395719 | 5.95E-34 | 4.85E-05 | 147.38 |
| rs7026972 | -0.0115 | 0.00109 | 3037499 | T | C | 0.649461 | 7.08E-26 | 3.66E-05 | 111.31 |
| rs80257979 | -0.02623 | 0.00295 | 3037499 | T | G | 0.970157 | 5.77E-19 | 2.60E-05 | 79.06 |
| rs9937449 | 0.00777 | 0.00105 | 3037499 | T | C | 0.550693 | 1.58E-13 | 1.80E-05 | 54.76 |
| rs34984805 | 0.0074 | 0.00105 | 3037499 | T | C | 0.530382 | 1.54E-12 | 1.64E-05 | 49.67 |
| rs72890842 | 0.0126 | 0.00122 | 3037499 | T | G | 0.765152 | 4.76E-25 | 3.51E-05 | 106.66 |
| rs9985296 | -0.01031 | 0.00108 | 3037499 | T | C | 0.384232 | 9.86E-22 | 3.00E-05 | 91.13 |
| rs9555651 | 0.01106 | 0.0014 | 3037499 | T | C | 0.155266 | 3.03E-15 | 2.05E-05 | 62.41 |
| rs61867294 | -0.01651 | 0.00131 | 3037499 | A | G | 0.78688 | 2.05E-36 | 5.23E-05 | 158.84 |
| rs7131691 | -0.00748 | 0.00105 | 3037499 | A | G | 0.484247 | 1.09E-12 | 1.67E-05 | 50.75 |
| rs4663617 | 0.01035 | 0.00123 | 3037499 | A | T | 0.230413 | 3.17E-17 | 2.33E-05 | 70.81 |
| rs1632822 | 0.00588 | 0.00108 | 3037499 | C | G | 0.369125 | 4.91E-08 | 9.76E-06 | 29.64 |
| rs10180845 | -0.0096 | 0.00109 | 3037499 | T | C | 0.656098 | 1.02E-18 | 2.55E-05 | 77.57 |
| rs2504846 | -0.02579 | 0.00328 | 3037499 | T | C | 0.031675 | 3.74E-15 | 2.04E-05 | 61.82 |
| rs73625120 | 0.01532 | 0.00271 | 3037499 | A | G | 0.04033 | 1.51E-08 | 1.05E-05 | 31.96 |
| rs474000 | 0.00642 | 0.00105 | 3037499 | A | G | 0.430474 | 1.10E-09 | 1.23E-05 | 37.38 |
| rs9859719 | -0.01128 | 0.00126 | 3037499 | A | G | 0.211395 | 3.67E-19 | 2.64E-05 | 80.15 |
| rs116173579 | -0.01649 | 0.00293 | 3037499 | T | C | 0.034755 | 1.74E-08 | 1.04E-05 | 31.67 |
| rs2430813 | 0.01103 | 0.00125 | 3037499 | T | C | 0.764875 | 8.62E-19 | 2.56E-05 | 77.86 |
| rs9930253 | -0.00986 | 0.00117 | 3037499 | A | T | 0.280551 | 3.12E-17 | 2.34E-05 | 71.02 |
| rs62172117 | 0.01356 | 0.0011 | 3037499 | A | G | 0.343286 | 3.35E-35 | 5.00E-05 | 151.96 |
| rs422247 | 0.00753 | 0.0012 | 3037499 | T | C | 0.739452 | 3.56E-10 | 1.30E-05 | 39.38 |
| rs7328289 | -0.00703 | 0.00127 | 3037499 | T | C | 0.794041 | 3.29E-08 | 1.01E-05 | 30.64 |
| rs565960 | 0.00875 | 0.00116 | 3037499 | T | C | 0.702325 | 3.75E-14 | 1.87E-05 | 56.9 |
| rs113389222 | -0.0117 | 0.0016 | 3037499 | T | C | 0.11409 | 2.68E-13 | 1.76E-05 | 53.47 |
| rs1738050 | -0.011 | 0.00108 | 3037499 | C | G | 0.630459 | 1.49E-24 | 3.42E-05 | 103.74 |
| rs158839 | -0.00623 | 0.00107 | 3037499 | A | C | 0.617278 | 6.42E-09 | 1.12E-05 | 33.9 |
| rs6580068 | -0.00931 | 0.00127 | 3037499 | T | C | 0.224638 | 1.96E-13 | 1.77E-05 | 53.74 |
| rs6906818 | 0.00823 | 0.00107 | 3037499 | T | C | 0.599384 | 1.26E-14 | 1.95E-05 | 59.16 |
| rs7449561 | 0.00973 | 0.00125 | 3037499 | A | G | 0.234278 | 6.83E-15 | 1.99E-05 | 60.59 |
| rs7304399 | 0.01093 | 0.00105 | 3037499 | A | G | 0.4585 | 2.16E-25 | 3.57E-05 | 108.36 |
| rs9540731 | 0.01075 | 0.00105 | 3037499 | T | C | 0.500693 | 9.93E-25 | 3.45E-05 | 104.82 |
| rs1490834 | -0.00639 | 0.00105 | 3037499 | A | G | 0.549892 | 1.15E-09 | 1.22E-05 | 37.04 |
| rs792213 | 0.00733 | 0.00106 | 3037499 | A | C | 0.41178 | 5.31E-12 | 1.57E-05 | 47.82 |
| rs300145 | -0.00726 | 0.00111 | 3037499 | T | C | 0.663736 | 5.87E-11 | 1.41E-05 | 42.78 |
| rs61964892 | -0.00785 | 0.00128 | 3037499 | T | C | 0.781999 | 8.76E-10 | 1.24E-05 | 37.61 |
| rs6801153 | -0.00856 | 0.00122 | 3037499 | T | C | 0.247351 | 2.11E-12 | 1.62E-05 | 49.23 |
| rs141503359 | -0.02835 | 0.00478 | 3037499 | T | C | 0.014259 | 2.97E-09 | 1.16E-05 | 35.18 |
| rs2174752 | -0.00862 | 0.00105 | 3037499 | T | G | 0.435186 | 2.36E-16 | 2.22E-05 | 67.4 |
| rs73055556 | 0.01112 | 0.00152 | 3037499 | A | G | 0.135587 | 2.51E-13 | 1.76E-05 | 53.52 |
| rs3869097 | -0.009 | 0.00112 | 3037499 | T | C | 0.333061 | 1.10E-15 | 2.13E-05 | 64.57 |
| rs4805761 | -0.01395 | 0.00145 | 3037499 | A | G | 0.150277 | 5.97E-22 | 3.05E-05 | 92.56 |
| rs6545559 | -0.00767 | 0.00112 | 3037499 | T | C | 0.316554 | 8.22E-12 | 1.54E-05 | 46.9 |
| rs2210174 | 0.00613 | 0.00108 | 3037499 | T | C | 0.622498 | 1.33E-08 | 1.06E-05 | 32.22 |
| rs4679353 | 0.00717 | 0.00108 | 3037499 | A | G | 0.614844 | 3.00E-11 | 1.45E-05 | 44.07 |
| rs6502670 | -0.00746 | 0.00109 | 3037499 | T | G | 0.617416 | 7.56E-12 | 1.54E-05 | 46.84 |
| rs72778512 | -0.01223 | 0.00185 | 3037499 | C | G | 0.913335 | 3.81E-11 | 1.44E-05 | 43.7 |
| rs4409028 | 0.00596 | 0.00109 | 3037499 | A | C | 0.63611 | 4.87E-08 | 9.84E-06 | 29.9 |
| rs12506222 | -0.01423 | 0.00105 | 3037499 | T | C | 0.467354 | 1.50E-41 | 6.05E-05 | 183.67 |
| rs1835388 | -0.00815 | 0.00105 | 3037499 | T | C | 0.482738 | 7.69E-15 | 1.98E-05 | 60.25 |
| rs5765717 | -0.00724 | 0.00105 | 3037499 | T | G | 0.478164 | 4.66E-12 | 1.57E-05 | 47.54 |
| rs116750901 | 0.0131 | 0.00237 | 3037499 | A | G | 0.038266 | 3.10E-08 | 1.01E-05 | 30.55 |
| rs1483814 | 0.00734 | 0.00131 | 3037499 | T | G | 0.191407 | 2.01E-08 | 1.03E-05 | 31.39 |
| rs6093705 | 0.00954 | 0.00112 | 3037499 | A | C | 0.682984 | 1.89E-17 | 2.39E-05 | 72.55 |
| rs3110127 | -0.00625 | 0.00111 | 3037499 | T | C | 0.349877 | 1.92E-08 | 1.04E-05 | 31.7 |
| rs837080 | -0.00977 | 0.00105 | 3037499 | T | C | 0.535048 | 1.07E-20 | 2.85E-05 | 86.58 |
| rs10202640 | -0.01027 | 0.00132 | 3037499 | A | G | 0.195966 | 8.23E-15 | 1.99E-05 | 60.53 |
| rs7240432 | 0.01039 | 0.00109 | 3037499 | T | G | 0.360687 | 1.74E-21 | 2.99E-05 | 90.86 |
| rs72717150 | -0.01942 | 0.00199 | 3037499 | A | G | 0.069464 | 1.68E-22 | 3.14E-05 | 95.23 |
| rs1880692 | 0.00767 | 0.00105 | 3037499 | A | G | 0.541839 | 2.78E-13 | 1.76E-05 | 53.36 |
| rs181164682 | 0.02436 | 0.00375 | 3037499 | A | T | 0.014999 | 8.16E-11 | 1.39E-05 | 42.2 |
| rs2239736 | 0.00856 | 0.00105 | 3037499 | T | C | 0.510286 | 3.04E-16 | 2.19E-05 | 66.46 |
| rs17088142 | -0.00683 | 0.00105 | 3037499 | T | G | 0.452787 | 7.51E-11 | 1.39E-05 | 42.31 |
| rs17649841 | -0.008 | 0.00123 | 3037499 | A | C | 0.227348 | 8.45E-11 | 1.39E-05 | 42.3 |
| rs74677779 | -0.01561 | 0.00257 | 3037499 | C | G | 0.956083 | 1.23E-09 | 1.21E-05 | 36.89 |
| rs35567946 | -0.00986 | 0.00106 | 3037499 | C | G | 0.586757 | 1.97E-20 | 2.85E-05 | 86.53 |
| rs9652254 | 0.00649 | 0.00106 | 3037499 | A | G | 0.602541 | 1.03E-09 | 1.23E-05 | 37.49 |
| rs6704768 | -0.01533 | 0.00106 | 3037499 | A | G | 0.541839 | 9.67E-48 | 6.89E-05 | 209.16 |
| rs11028323 | -0.00984 | 0.00105 | 3037499 | A | C | 0.514675 | 6.20E-21 | 2.89E-05 | 87.82 |
| rs17224289 | -0.01317 | 0.00161 | 3037499 | T | C | 0.117632 | 3.10E-16 | 2.20E-05 | 66.91 |
| rs6960056 | 0.01286 | 0.00105 | 3037499 | A | G | 0.450616 | 3.50E-34 | 4.94E-05 | 150 |
| rs7979979 | -0.01165 | 0.00122 | 3037499 | T | C | 0.226548 | 1.20E-21 | 3.00E-05 | 91.19 |
| rs4726070 | 0.01021 | 0.00107 | 3037499 | A | G | 0.593086 | 1.05E-21 | 3.00E-05 | 91.05 |
| rs10186870 | -0.00602 | 0.00107 | 3037499 | T | G | 0.605759 | 1.91E-08 | 1.04E-05 | 31.65 |
| rs17565975 | -0.01211 | 0.00105 | 3037499 | A | G | 0.529504 | 1.25E-30 | 4.38E-05 | 133.02 |
| rs2214631 | 0.00706 | 0.00117 | 3037499 | A | T | 0.311287 | 1.43E-09 | 1.20E-05 | 36.41 |
| rs11773992 | -0.0138 | 0.00137 | 3037499 | C | G | 0.16178 | 5.84E-24 | 3.34E-05 | 101.47 |
| rs4715244 | 0.00716 | 0.00126 | 3037499 | T | G | 0.212119 | 1.28E-08 | 1.06E-05 | 32.29 |
| rs7677621 | -0.01029 | 0.00109 | 3037499 | T | C | 0.355328 | 5.54E-21 | 2.93E-05 | 89.12 |
| rs2021871 | 0.00654 | 0.00112 | 3037499 | T | C | 0.66854 | 4.87E-09 | 1.12E-05 | 34.1 |
| rs17096452 | 0.01138 | 0.00127 | 3037499 | A | T | 0.791284 | 3.18E-19 | 2.64E-05 | 80.29 |
| rs72779695 | -0.01397 | 0.00162 | 3037499 | T | C | 0.11466 | 7.03E-18 | 2.45E-05 | 74.36 |
| rs34316 | 0.01629 | 0.00106 | 3037499 | A | C | 0.434694 | 6.58E-53 | 7.77E-05 | 236.17 |
| rs76076331 | 0.02206 | 0.00156 | 3037499 | T | C | 0.122605 | 1.54E-45 | 6.58E-05 | 199.97 |
| rs10256396 | 0.00654 | 0.00111 | 3037499 | A | C | 0.35539 | 3.34E-09 | 1.14E-05 | 34.71 |
| rs12151015 | -0.00956 | 0.00164 | 3037499 | C | G | 0.888343 | 5.60E-09 | 1.12E-05 | 33.98 |
| rs2916490 | -0.01042 | 0.00113 | 3037499 | A | G | 0.304697 | 4.04E-20 | 2.80E-05 | 85.03 |
| rs62086577 | 0.00739 | 0.00128 | 3037499 | A | G | 0.210625 | 8.10E-09 | 1.10E-05 | 33.33 |
| rs10895719 | -0.01068 | 0.00128 | 3037499 | T | C | 0.777826 | 6.65E-17 | 2.29E-05 | 69.62 |
| rs77899607 | 0.01308 | 0.00157 | 3037499 | T | C | 0.108131 | 6.50E-17 | 2.29E-05 | 69.41 |
| rs1884368 | 0.00909 | 0.00122 | 3037499 | T | C | 0.232338 | 7.73E-14 | 1.83E-05 | 55.51 |
| rs13244634 | -0.00784 | 0.00123 | 3037499 | T | C | 0.238512 | 2.10E-10 | 1.34E-05 | 40.63 |
| rs2304282 | 0.011 | 0.00106 | 3037499 | A | G | 0.407407 | 4.04E-25 | 3.55E-05 | 107.69 |
| rs7039819 | 0.00919 | 0.00106 | 3037499 | A | G | 0.58146 | 4.39E-18 | 2.47E-05 | 75.17 |
| rs10496632 | 0.01114 | 0.00116 | 3037499 | C | G | 0.288112 | 8.30E-22 | 3.04E-05 | 92.23 |
| rs11138947 | 0.00958 | 0.00117 | 3037499 | T | C | 0.711718 | 2.22E-16 | 2.21E-05 | 67.04 |
| rs4989480 | -0.00887 | 0.00151 | 3037499 | A | G | 0.856683 | 4.02E-09 | 1.14E-05 | 34.51 |
| rs72838750 | -0.00877 | 0.00105 | 3037499 | T | G | 0.502418 | 8.35E-17 | 2.30E-05 | 69.76 |
| rs4911257 | 0.00979 | 0.00107 | 3037499 | T | C | 0.606729 | 7.96E-20 | 2.76E-05 | 83.71 |
| rs12962980 | 0.01682 | 0.00136 | 3037499 | C | G | 0.187743 | 2.40E-35 | 5.04E-05 | 152.96 |
| rs10765789 | 0.01078 | 0.00108 | 3037499 | A | G | 0.369233 | 1.35E-23 | 3.28E-05 | 99.63 |
| rs2352430 | -0.00783 | 0.00124 | 3037499 | T | C | 0.77048 | 3.11E-10 | 1.31E-05 | 39.87 |
| rs61160187 | -0.01963 | 0.00107 | 3037499 | A | G | 0.608362 | 1.32E-74 | 1.11E-04 | 336.57 |
| rs1407350 | 0.00784 | 0.0011 | 3037499 | T | G | 0.646705 | 9.13E-13 | 1.67E-05 | 50.8 |
| rs6930903 | 0.01587 | 0.00133 | 3037499 | A | G | 0.738374 | 4.73E-33 | 4.69E-05 | 142.38 |
| rs2867932 | 0.00715 | 0.00107 | 3037499 | A | G | 0.38956 | 2.65E-11 | 1.47E-05 | 44.65 |
| rs35414759 | -0.01094 | 0.00135 | 3037499 | T | C | 0.830274 | 4.69E-16 | 2.16E-05 | 65.67 |
| rs117494884 | -0.01456 | 0.00225 | 3037499 | T | C | 0.054512 | 1.04E-10 | 1.38E-05 | 41.88 |
| rs17669337 | -0.01008 | 0.00106 | 3037499 | T | C | 0.405174 | 2.46E-21 | 2.98E-05 | 90.43 |
| rs1452366 | -0.01191 | 0.0011 | 3037499 | T | G | 0.352618 | 1.58E-27 | 3.86E-05 | 117.23 |
| rs72672601 | -0.00953 | 0.00106 | 3037499 | A | G | 0.578873 | 2.02E-19 | 2.66E-05 | 80.83 |
| rs1378893 | 0.00908 | 0.00123 | 3037499 | A | G | 0.22627 | 1.52E-13 | 1.79E-05 | 54.5 |
| rs60307735 | 0.00923 | 0.00111 | 3037499 | T | C | 0.329489 | 6.83E-17 | 2.28E-05 | 69.14 |
| rs4850810 | -0.01315 | 0.00105 | 3037499 | T | G | 0.519957 | 6.68E-36 | 5.16E-05 | 156.85 |
| rs34305371 | 0.03143 | 0.00176 | 3037499 | A | G | 0.085787 | 1.77E-71 | 1.05E-04 | 318.91 |
| rs10468056 | 0.01137 | 0.00111 | 3037499 | T | G | 0.358608 | 9.50E-25 | 3.45E-05 | 104.92 |
| rs12126231 | 0.01174 | 0.00108 | 3037499 | A | G | 0.615984 | 9.70E-28 | 3.89E-05 | 118.16 |
| rs35951883 | -0.00805 | 0.00127 | 3037499 | A | G | 0.790345 | 2.33E-10 | 1.32E-05 | 40.18 |
| rs28438078 | -0.00964 | 0.00143 | 3037499 | A | G | 0.142393 | 1.72E-11 | 1.50E-05 | 45.44 |
| rs55975662 | 0.01532 | 0.00149 | 3037499 | A | G | 0.84612 | 1.09E-24 | 3.48E-05 | 105.72 |
| rs1871745 | 0.01019 | 0.00129 | 3037499 | A | G | 0.765938 | 2.19E-15 | 2.05E-05 | 62.4 |
| rs79708660 | -0.0087 | 0.00128 | 3037499 | A | G | 0.207191 | 1.09E-11 | 1.52E-05 | 46.2 |
| rs1516036 | 0.00707 | 0.00105 | 3037499 | A | G | 0.546905 | 1.57E-11 | 1.49E-05 | 45.34 |
| rs2216053 | -0.00727 | 0.00119 | 3037499 | T | G | 0.736742 | 1.06E-09 | 1.23E-05 | 37.32 |
| rs77128898 | -0.0242 | 0.00295 | 3037499 | T | C | 0.030952 | 2.09E-16 | 2.22E-05 | 67.3 |
| rs2555506 | 0.00926 | 0.00138 | 3037499 | A | G | 0.169187 | 1.94E-11 | 1.48E-05 | 45.03 |
| rs13122283 | 0.00695 | 0.00106 | 3037499 | A | G | 0.403203 | 5.08E-11 | 1.42E-05 | 42.99 |
| rs4127499 | 0.01096 | 0.0011 | 3037499 | A | G | 0.324962 | 2.90E-23 | 3.27E-05 | 99.27 |
| rs12431579 | -0.00609 | 0.00109 | 3037499 | A | G | 0.639837 | 2.25E-08 | 1.03E-05 | 31.22 |
| rs58694847 | -0.01174 | 0.00119 | 3037499 | C | G | 0.275593 | 7.84E-23 | 3.20E-05 | 97.33 |
| rs73874322 | -0.01431 | 0.00217 | 3037499 | C | G | 0.056637 | 4.21E-11 | 1.43E-05 | 43.49 |
| rs6782698 | 0.01383 | 0.00121 | 3037499 | A | G | 0.758007 | 4.81E-30 | 4.30E-05 | 130.64 |
| rs6715321 | -0.00979 | 0.00106 | 3037499 | T | C | 0.439205 | 2.20E-20 | 2.81E-05 | 85.3 |
| rs56355837 | -0.00842 | 0.00111 | 3037499 | A | C | 0.685864 | 2.94E-14 | 1.89E-05 | 57.54 |
| rs72993796 | 0.01485 | 0.00163 | 3037499 | T | C | 0.891946 | 8.58E-20 | 2.73E-05 | 83 |
| rs10063055 | -0.01249 | 0.00123 | 3037499 | T | C | 0.229689 | 3.26E-24 | 3.39E-05 | 103.11 |
| rs62199988 | -0.00862 | 0.00107 | 3037499 | C | G | 0.622174 | 7.87E-16 | 2.14E-05 | 64.9 |
| rs669952 | 0.0078 | 0.00134 | 3037499 | A | G | 0.181583 | 6.31E-09 | 1.12E-05 | 33.88 |
| rs708912 | 0.01037 | 0.00129 | 3037499 | T | C | 0.780074 | 9.00E-16 | 2.13E-05 | 64.62 |
| rs77926719 | 0.01097 | 0.0016 | 3037499 | A | G | 0.106606 | 7.25E-12 | 1.55E-05 | 47.01 |
| rs72725852 | 0.00663 | 0.00112 | 3037499 | T | C | 0.316184 | 3.28E-09 | 1.15E-05 | 35.04 |
| rs72829857 | -0.01705 | 0.00121 | 3037499 | A | G | 0.759871 | 7.31E-45 | 6.54E-05 | 198.55 |
| rs10853981 | -0.00868 | 0.00111 | 3037499 | A | G | 0.325146 | 6.64E-15 | 2.01E-05 | 61.15 |
| rs1462852 | -0.00729 | 0.00111 | 3037499 | A | T | 0.646951 | 5.27E-11 | 1.42E-05 | 43.13 |
| rs10740572 | -0.00844 | 0.00126 | 3037499 | A | T | 0.777795 | 1.92E-11 | 1.48E-05 | 44.87 |
| rs11643516 | -0.00812 | 0.00106 | 3037499 | T | C | 0.43166 | 2.22E-14 | 1.93E-05 | 58.68 |
| rs7933521 | 0.00826 | 0.00149 | 3037499 | T | C | 0.839452 | 2.85E-08 | 1.01E-05 | 30.73 |
| rs7520927 | -0.00684 | 0.00105 | 3037499 | A | G | 0.478226 | 6.38E-11 | 1.40E-05 | 42.44 |
| rs11157931 | -0.01152 | 0.00107 | 3037499 | A | C | 0.396628 | 5.98E-27 | 3.82E-05 | 115.91 |
| rs117520198 | 0.0107 | 0.00133 | 3037499 | A | G | 0.1747 | 1.01E-15 | 2.13E-05 | 64.72 |
| rs6028084 | 0.01514 | 0.00107 | 3037499 | T | C | 0.610764 | 3.44E-45 | 6.59E-05 | 200.21 |
| rs7515450 | 0.00802 | 0.00107 | 3037499 | A | T | 0.410718 | 7.28E-14 | 1.85E-05 | 56.18 |
| rs116068530 | 0.01945 | 0.00353 | 3037499 | A | G | 0.019603 | 3.71E-08 | 9.99E-06 | 30.36 |
| rs213014 | -0.00673 | 0.00105 | 3037499 | T | C | 0.548352 | 1.49E-10 | 1.35E-05 | 41.08 |
| rs13161488 | -0.00869 | 0.0011 | 3037499 | T | C | 0.659578 | 2.45E-15 | 2.05E-05 | 62.41 |
| rs3731507 | 0.02758 | 0.00259 | 3037499 | T | C | 0.05211 | 1.71E-26 | 3.73E-05 | 113.39 |
| rs13030436 | 0.00787 | 0.00106 | 3037499 | T | C | 0.408777 | 8.77E-14 | 1.81E-05 | 55.12 |
| rs76799289 | -0.02828 | 0.00449 | 3037499 | T | G | 0.009763 | 2.96E-10 | 1.31E-05 | 39.67 |
| rs35226705 | 0.00614 | 0.00105 | 3037499 | A | C | 0.543686 | 4.86E-09 | 1.13E-05 | 34.19 |
| rs2238483 | 0.01009 | 0.0014 | 3037499 | A | G | 0.814721 | 5.31E-13 | 1.71E-05 | 51.94 |
| rs62156740 | -0.02875 | 0.00387 | 3037499 | A | T | 0.985063 | 1.17E-13 | 1.82E-05 | 55.19 |
| rs912609 | 0.01235 | 0.00125 | 3037499 | T | G | 0.240437 | 6.57E-23 | 3.21E-05 | 97.61 |
| rs9359939 | -0.01085 | 0.00122 | 3037499 | A | C | 0.237558 | 6.65E-19 | 2.60E-05 | 79.09 |
| rs3747631 | 0.02043 | 0.00127 | 3037499 | C | G | 0.201217 | 7.17E-58 | 8.52E-05 | 258.78 |
| rs9411336 | -0.01285 | 0.00112 | 3037499 | T | C | 0.337296 | 1.56E-30 | 4.33E-05 | 131.63 |
| rs4882141 | 0.00662 | 0.0011 | 3037499 | T | C | 0.649091 | 1.53E-09 | 1.19E-05 | 36.22 |
| rs975303 | -0.01477 | 0.00138 | 3037499 | A | G | 0.839082 | 1.17E-26 | 3.77E-05 | 114.55 |
| rs117520996 | 0.02196 | 0.0025 | 3037499 | T | G | 0.041638 | 1.47E-18 | 2.54E-05 | 77.16 |
| rs1888765 | 0.00901 | 0.00126 | 3037499 | C | G | 0.767 | 7.09E-13 | 1.68E-05 | 51.13 |
| rs4447403 | 0.00695 | 0.0012 | 3037499 | T | C | 0.742947 | 6.98E-09 | 1.10E-05 | 33.54 |
| rs76878669 | 0.01212 | 0.00123 | 3037499 | C | G | 0.766677 | 6.48E-23 | 3.20E-05 | 97.09 |
| rs17248751 | -0.01375 | 0.00127 | 3037499 | A | G | 0.798676 | 3.42E-27 | 3.86E-05 | 117.22 |
| rs2764684 | 0.01501 | 0.00139 | 3037499 | T | C | 0.847028 | 2.64E-27 | 3.84E-05 | 116.61 |
| rs28458909 | -0.01902 | 0.00164 | 3037499 | T | C | 0.126255 | 5.98E-31 | 4.43E-05 | 134.5 |
| rs6088618 | 0.00813 | 0.00105 | 3037499 | A | G | 0.438405 | 1.08E-14 | 1.97E-05 | 59.95 |
| rs10283803 | -0.00575 | 0.00105 | 3037499 | T | C | 0.531306 | 3.91E-08 | 9.87E-06 | 29.99 |
| rs7151326 | -0.0087 | 0.00107 | 3037499 | T | G | 0.412242 | 5.69E-16 | 2.18E-05 | 66.11 |
| rs8110747 | -0.00659 | 0.00106 | 3037499 | T | C | 0.389051 | 6.03E-10 | 1.27E-05 | 38.65 |
| rs56378598 | 0.00826 | 0.00119 | 3037499 | T | C | 0.290607 | 4.27E-12 | 1.59E-05 | 48.18 |
| rs13278836 | 0.01373 | 0.00164 | 3037499 | A | G | 0.110748 | 5.10E-17 | 2.31E-05 | 70.09 |
| rs9899056 | 0.00698 | 0.00107 | 3037499 | A | G | 0.397151 | 6.71E-11 | 1.40E-05 | 42.55 |
| rs273028 | -0.01353 | 0.00199 | 3037499 | A | T | 0.085756 | 9.32E-12 | 1.52E-05 | 46.23 |
| rs7650602 | -0.00998 | 0.00106 | 3037499 | T | C | 0.567601 | 3.19E-21 | 2.92E-05 | 88.64 |
| rs78280128 | -0.01989 | 0.00187 | 3037499 | A | G | 0.920234 | 1.98E-26 | 3.72E-05 | 113.13 |
| rs12981405 | -0.01106 | 0.00141 | 3037499 | T | C | 0.158577 | 4.09E-15 | 2.03E-05 | 61.53 |
| rs55897719 | -0.01267 | 0.00115 | 3037499 | A | C | 0.286973 | 2.04E-28 | 4.00E-05 | 121.38 |
| rs3108680 | 0.0102 | 0.0011 | 3037499 | T | C | 0.668032 | 1.89E-20 | 2.83E-05 | 85.98 |
| rs717997 | 0.01297 | 0.00106 | 3037499 | A | G | 0.421312 | 2.49E-34 | 4.93E-05 | 149.72 |
| rs10189857 | 0.01378 | 0.00106 | 3037499 | A | G | 0.562889 | 7.74E-39 | 5.56E-05 | 169 |
| rs35733856 | -0.01188 | 0.00107 | 3037499 | A | G | 0.559732 | 8.26E-29 | 4.06E-05 | 123.27 |
| rs62543169 | -0.01165 | 0.00106 | 3037499 | T | C | 0.404327 | 3.33E-28 | 3.98E-05 | 120.79 |
| rs11245450 | 0.00686 | 0.00106 | 3037499 | A | G | 0.420157 | 1.06E-10 | 1.38E-05 | 41.88 |
| rs7626224 | 0.00784 | 0.00122 | 3037499 | T | G | 0.76215 | 1.36E-10 | 1.36E-05 | 41.3 |
| rs6956241 | 0.01341 | 0.00151 | 3037499 | T | G | 0.144995 | 5.88E-19 | 2.60E-05 | 78.87 |
| rs192436652 | -0.02765 | 0.00333 | 3037499 | T | C | 0.027302 | 9.90E-17 | 2.27E-05 | 68.94 |
| rs12435895 | -0.00798 | 0.00125 | 3037499 | A | G | 0.773961 | 1.55E-10 | 1.34E-05 | 40.76 |
| rs853199 | 0.00859 | 0.00109 | 3037499 | T | C | 0.332476 | 3.84E-15 | 2.04E-05 | 62.11 |
| rs4358081 | -0.0097 | 0.00105 | 3037499 | A | C | 0.533554 | 2.14E-20 | 2.81E-05 | 85.34 |
| rs12273435 | -0.01409 | 0.00131 | 3037499 | A | G | 0.187866 | 6.11E-27 | 3.81E-05 | 115.69 |
| rs11218422 | 0.00879 | 0.00106 | 3037499 | T | C | 0.565907 | 1.10E-16 | 2.26E-05 | 68.76 |
| rs17126938 | -0.01264 | 0.00151 | 3037499 | T | C | 0.858285 | 5.79E-17 | 2.31E-05 | 70.07 |
| rs4557790 | 0.02049 | 0.00105 | 3037499 | A | C | 0.567154 | 4.88E-84 | 1.25E-04 | 380.81 |
| rs75624576 | 0.01536 | 0.00175 | 3037499 | T | G | 0.096274 | 1.36E-18 | 2.54E-05 | 77.04 |
| rs61747226 | -0.0189 | 0.0026 | 3037499 | T | C | 0.040946 | 3.41E-13 | 1.74E-05 | 52.84 |
| rs56059718 | -0.01216 | 0.00132 | 3037499 | A | C | 0.196951 | 3.81E-20 | 2.79E-05 | 84.86 |
| rs11774212 | 0.01567 | 0.00105 | 3037499 | T | C | 0.496458 | 1.70E-50 | 7.33E-05 | 222.72 |
| rs10401329 | 0.01094 | 0.00161 | 3037499 | A | G | 0.136865 | 1.22E-11 | 1.52E-05 | 46.17 |
| rs4798783 | -0.01152 | 0.00115 | 3037499 | A | G | 0.705405 | 1.26E-23 | 3.30E-05 | 100.35 |
| rs17257579 | -0.0131 | 0.00159 | 3037499 | T | C | 0.878703 | 1.40E-16 | 2.23E-05 | 67.88 |
| rs863006 | -0.00984 | 0.00106 | 3037499 | A | G | 0.558223 | 1.31E-20 | 2.84E-05 | 86.17 |
| rs489408 | 0.00766 | 0.00105 | 3037499 | A | G | 0.436326 | 3.25E-13 | 1.75E-05 | 53.22 |
| rs12709690 | -0.01858 | 0.00109 | 3037499 | T | C | 0.372621 | 9.28E-65 | 9.56E-05 | 290.56 |
| rs11196397 | 0.00897 | 0.00117 | 3037499 | A | C | 0.26158 | 2.22E-14 | 1.94E-05 | 58.78 |
| rs72801843 | 0.01519 | 0.00114 | 3037499 | A | T | 0.292331 | 1.78E-40 | 5.84E-05 | 177.54 |
| rs952062 | 0.01023 | 0.00169 | 3037499 | A | T | 0.127333 | 1.30E-09 | 1.21E-05 | 36.64 |
| rs7097348 | -0.00905 | 0.00116 | 3037499 | T | C | 0.724669 | 6.44E-15 | 2.00E-05 | 60.87 |
| rs2668196 | -0.01186 | 0.00132 | 3037499 | A | T | 0.207961 | 3.28E-19 | 2.66E-05 | 80.73 |
| rs28807201 | -0.01139 | 0.00114 | 3037499 | T | C | 0.684416 | 1.97E-23 | 3.29E-05 | 99.82 |
| rs7184863 | 0.00803 | 0.00136 | 3037499 | T | G | 0.802371 | 3.72E-09 | 1.15E-05 | 34.86 |
| rs738988 | -0.01605 | 0.00116 | 3037499 | A | G | 0.737681 | 1.36E-43 | 6.30E-05 | 191.44 |
| rs6713695 | -0.0106 | 0.00105 | 3037499 | A | G | 0.457699 | 5.18E-24 | 3.36E-05 | 101.91 |
| rs62439683 | -0.01207 | 0.00121 | 3037499 | A | C | 0.244071 | 1.55E-23 | 3.28E-05 | 99.5 |
| rs149285517 | -0.01187 | 0.00164 | 3037499 | T | C | 0.122698 | 4.61E-13 | 1.72E-05 | 52.39 |
| rs7095620 | -0.00746 | 0.00113 | 3037499 | A | G | 0.325516 | 4.32E-11 | 1.43E-05 | 43.58 |
| rs34743418 | -0.00999 | 0.00106 | 3037499 | T | C | 0.463998 | 5.01E-21 | 2.92E-05 | 88.82 |
| rs16983844 | 0.01074 | 0.00129 | 3037499 | A | G | 0.188266 | 7.86E-17 | 2.28E-05 | 69.32 |
| rs1043595 | 0.01663 | 0.00118 | 3037499 | A | G | 0.261241 | 2.36E-45 | 6.54E-05 | 198.62 |
| rs57985238 | -0.00909 | 0.00107 | 3037499 | C | G | 0.612873 | 1.99E-17 | 2.38E-05 | 72.17 |
| rs7578247 | -0.00798 | 0.00109 | 3037499 | A | G | 0.608978 | 2.25E-13 | 1.76E-05 | 53.6 |
| rs73219806 | 0.0124 | 0.00141 | 3037499 | A | C | 0.172267 | 1.34E-18 | 2.55E-05 | 77.34 |
| rs10929474 | 0.01145 | 0.00128 | 3037499 | A | T | 0.224669 | 5.12E-19 | 2.63E-05 | 80.02 |
| rs6907381 | -0.01185 | 0.00159 | 3037499 | A | G | 0.130644 | 9.09E-14 | 1.83E-05 | 55.54 |
| rs1193240 | 0.00847 | 0.00106 | 3037499 | A | C | 0.423252 | 1.73E-15 | 2.10E-05 | 63.85 |
| rs336433 | 0.00775 | 0.00105 | 3037499 | A | G | 0.421805 | 2.05E-13 | 1.79E-05 | 54.48 |
| rs3095075 | -0.0136 | 0.00105 | 3037499 | A | G | 0.550108 | 3.81E-38 | 5.52E-05 | 167.76 |
| rs2177083 | -0.00741 | 0.00105 | 3037499 | A | G | 0.50214 | 1.49E-12 | 1.64E-05 | 49.8 |
| rs13133213 | 0.00873 | 0.00105 | 3037499 | A | G | 0.502233 | 7.24E-17 | 2.28E-05 | 69.13 |
| rs3098650 | 0.01181 | 0.00108 | 3037499 | C | G | 0.602464 | 7.71E-28 | 3.94E-05 | 119.58 |
| rs10507085 | -0.01224 | 0.002 | 3037499 | T | C | 0.068063 | 9.14E-10 | 1.23E-05 | 37.45 |

**SNP**, Single nucleotide polymorphism; **Pos**, position for SNP; **Chr**, chromosome; **SE**, standard error; **EAF**, effect allele frequency; **P-value**, the value for the genetic association.
